# An optimized background regimen design to evaluate the contribution of levofloxacin to multidrug-resistant tuberculosis treatment regimens: study protocol for a randomized controlled trial

**DOI:** 10.1186/s13063-017-2292-x

**Published:** 2017-11-25

**Authors:** Tara C. Bouton, Patrick P. J. Phillips, Carole D. Mitnick, Charles A. Peloquin, Kathleen Eisenach, Ramonde F. Patientia, Leonid Lecca, Eduardo Gotuzzo, Neel R. Gandhi, Donna Butler, Andreas H. Diacon, Bruno Martel, Juan Santillan, Kathleen Robergeau Hunt, Dante Vargas, Florian von Groote-Bidlingmaier, Carlos Seas, Nancy Dianis, Antonio Moreno-Martinez, C. Robert Horsburgh

**Affiliations:** 1 Brown University Alpert School of Medicine, Providence, RI USA; 2 Medical Research Council Clinical Trials Unit at University College London, London, UK; 3 Department of Global Health & Social Medicine, Harvard Medical School, Boston, MA USA; 4 Infectious Disease Pharmacokinetics Lab, University of Florida, Gainesville, FL USA; 5 University of Arkansas for Medical Sciences, Little Rock, AR USA; 6 Stellenbosch University and Task Applied Science, Cape Town, South Africa; 7 Socios en Salud Sucursal Peru, Lima, Peru; 8 Universidad Peruana Cayetano Heredia, Lima, Peru; 9 Departments of Epidemiology, Global Health & Infectious Diseases, Rollins School of Public Health and Emory School of Medicine, Emory University, Atlanta, GA USA; 10 Westat, Bethesda, MA USA; 11 TB Investigation Unit of Barcelona, Barcelona, Spain; 12 CIBER de Epidemiología y Salud Pública (CIBERESP), Barcelona, Spain; 13 Department of Medicine, Boston University School of Medicine, Boston, MA USA; 14 Departments of Epidemiology, Biostatistics and Global Health, Boston University School of Public Health, 715 Albany Street, T3E, Boston, MA 02118 USA

**Keywords:** Multidrug resistant tuberculosis, Optimized background regimen, Fluoroquinolones, Levofloxacin

## Abstract

**Background:**

Current guidelines for treatment of multidrug-resistant tuberculosis (MDR-TB) are largely based on expert opinion and observational data. Fluoroquinolones remain an essential part of MDR-TB treatment, but the optimal dose of fluoroquinolones as part of the regimen has not been defined.

**Methods/design:**

We designed a randomized, blinded, phase II trial in MDR-TB patients comparing across levofloxacin doses of 11, 14, 17 and 20 mg/kg/day, all within an optimized background regimen. We assess pharmacokinetics, efficacy, safety and tolerability of regimens containing each of these doses. The primary efficacy outcome is time to culture conversion over the first 6 months of treatment. The study aims to determine the area under the curve (AUC) of the levofloxacin serum concentration in the 24 hours after dosing divided by the minimal inhibitory concentration of the patient’s *Mycobacterium tuberculosis* isolate that inhibits > 90% of organisms (AUC/MIC) that maximizes efficacy and the AUC that maximizes safety and tolerability in the context of an MDR-TB treatment regimen.

**Discussion:**

Fluoroquinolones are an integral part of recommended MDR-TB regimens. Little is known about how to optimize dosing for efficacy while maintaining acceptable toxicity. This study will provide evidence to support revised dosing guidelines for the use of levofloxacin as part of combination regimens for treatment of MDR-TB. The novel methodology can be adapted to elucidate the effect of other single agents in multidrug antibiotic treatment regimens.

**Trial registration:**

ClinicalTrials.gov, NCT01918397. Registered on 5 August 2013.

**Electronic supplementary material:**

The online version of this article (doi:10.1186/s13063-017-2292-x) contains supplementary material, which is available to authorized users.

## Background

Multidrug-resistant tuberculosis (MDR-TB), defined by resistance to isoniazid and rifampicin, is increasingly common. In 2015, there were an estimated 580,000 new cases eligible for MDR-TB treatment globally. Fewer than 100,000 of these patients were started on an MDR-TB treatment regimen. Of these, only 52% are likely to have a successful outcome, while 41% may die, be lost to follow up or experience treatment failure [1]. Despite increasing world-wide prevalence of drug-resistant tuberculosis, guidelines on regimen recommendations are largely based on expert opinion and observational studies [2]. Third generation fluoroquinolones (FQ) are an integral part of MDR-TB regimens, based on several observational studies that have shown improved treatment outcomes with the use of FQs [3]. In addition, all regimens recommended in international guidelines or being studied in ongoing clinical trials include an FQ, showing its importance in current and future MDR-TB regimens.

Fluoroquinolones target the DNA gyrase of bacteria, including that of *Mycobacterium tuberculosis*. Newer members of the FQ class, levofloxacin, moxifloxacin and gatifloxacin, have greater anti-tuberculosis activity in vitro than ciprofloxacin or ofloxacin [4]. Improved pharmacokinetics and pharmacodynamics have also been observed with the later-generation fluoroquinolones [5]. Though there has been concern about increased risk of fatal arrhythmia with other FQs, QT studies of levofloxacin have found no QT prolongation at daily doses of up to 1500 mg [6].

The two newest agents for treatment of MDR-TB, bedaquiline and delamanid, have both been associated with QT prolongation [7, 8]. At the end of 2015, 70 countries had reported using bedaquiline in regimens to treat MDR-TB and 39 had reported using delamanid [9]. As access to these newer agents continues to increase globally, so does the need for further information on optimal levofloxacin dosing for both efficacy and tolerability. Because of its minimal QT prolongation compared to moxifloxacin and the general unavailability of gatifloxacin, levofloxacin remains the preferred drug for administration in combination with either bedaquiline or delamanid [6, 10, 11]. We therefore designed and implemented a study to determine the target area under the curve (AUC) for this agent that was associated with optimal efficacy and acceptable tolerability.

Heterogeneous patterns of drug resistance in MDR-TB, variability in background regimens and complexity of the disease have led to challenges in clinical trial design. The optimized background regimen design, first used to evaluate regimens for drug-resistant human immunodeficiency virus (HIV), has been increasingly accepted for evaluation of individual drugs in multidrug regimens [7, 8, 12]. Although this design is not appropriate for developing regimens for MDR-TB, it is useful for identifying the optimal dose of single drugs. Using this design, patients are randomized to either receive a multi-drug standard-of-care regimen (the optimized background regimen, OBR) plus placebo or the OBR plus the investigational agent.

Levofloxacin is currently recommended as a fixed dose of 750 − 1000 mg/day for adult patients [2]. This results in substantial variability in the serum levels of levofloxacin, and in activity and tolerability, since both are concentration dependent [13, 14]. In a disease such as MDR-TB, where there are not many effective drugs, optimal dosing of available agents is essential. The pharmacokinetic-pharmacodynamic marker that best predicts the efficacy of levofloxacin is the ratio of the AUC to the MIC (AUC/MIC) [13]. It is paramount that patients with MDR-TB receive enough drug to achieve AUC/MIC to provide reasonable certainty of the optimal antibacterial effect. We therefore designed a placebo-controlled study in which patients are randomized to one of four levofloxacin doses in addition to an OBR, and all study participants receive at least the currently accepted standard levofloxacin dose (750 mg/day).

### Study goal and objectives

The goal of the Opti-Q study is to determine the levofloxacin AUC/MIC that is associated with the greatest reduction in mycobacterial burden with acceptable safety and tolerability in patients with MDR-TB, and to facilitate development of a dosing algorithm to achieve that AUC in as many patients as possible.

The primary objectives are to:

1. Determine the levofloxacin AUC/MIC that provides the shortest time to sputum culture conversion on solid medium
2. Determine the highest levofloxacin AUC that is both safe and associated with fewer than 25% of patients discontinuing or reducing the dose of levofloxacin

## Methods

### Site selection

Clinical recruitment sites were first identified from within the CDC Tuberculosis Trials Consortium (TBTC). Sites in Cape Town, South Africa and Lima, Peru were selected. Since the TBTC did not have access to sufficient numbers of patients with MDR-TB at other sites, a third non-TBTC site in Lima, Peru was also selected and supported by a grant from the Division of Microbiology and Infectious Disease at the National Institute of Allergy and Infectious Disease at the US National Institutes of Health (NIH Grant AI100805). All three clinical sites (two in Lima and one in Cape Town) have a successful history of clinical trials funded either through the NIH or TBTC.

### Study population and eligibility

Potentially eligible patients include adults with smear-positive pulmonary MDR-TB, who are willing to attend follow up visits and undergo study assessments, and able to provide informed consent (see Table 1). Opti-Q uses the results of MTBDRplus and MTBDRsl tests to define study eligibility criteria. Those showing isoniazid and rifampin resistance and FQ susceptibility on these two molecular tests are eligible. Other criteria for inclusion are known HIV status regardless of result and therapy, weight > =40 kg, and Karnofsky score ≥60.

**Table 1:** Opti-Q study participant inclusion and exclusion criteria

Randomization inclusion criteria
1. Patients with pulmonary TB
2. Sputum that is isoniazid and rifampin-resistant by MTBDRplus and fluoroquinolone = susceptible by MTBDRsl
3. HIV seropositive or seronegative but not unknown HIV serostatus. If the last documented negative HIV test was more than 3 months prior to randomization the current serostatus must be assessed
4. Age ≥18 years
5. Weight ≥40 kg
6. Karnofsky score ≥60 at screening and randomization
7. Willingness by the patient to attend scheduled follow up visits and undergo study assessments.
8. Women with child-bearing potential must agree to practice adequate birth control or to abstain from heterosexual intercourse during study regimen
9. Laboratory parameters (performed within 14 days prior to randomization):
• Estimated Serum creatinine clearance should be ≥ 50, using nomogram
• Hemoglobin concentration ≥ 9.0 g/dL
• Platelet count ≥80,000/mm3
• Absolute neutrophil count (ANC) >1000/mm3
• Negative pregnancy test (for women of childbearing potential) during randomization/baseline
• CD4 count if HIV infected (within 6 months)
• Serum ALT and total bilirubin <3 times upper limit of normal
10. Able to provide informed consent
Randomization exclusion criteria
1. Currently breast-feeding or pregnant
2. Known allergy or intolerance to or toxicity from fluoroquinolones or other medications utilized in this study
3. In the judgment of the physician the patient is not expected to survive for 6 months
4. Anticipated surgical intervention for the treatment of pulmonary TB
5. Participation in another investigational drug trial within the past 30 days
6. Concurrent use of known QT-prolonging drugs: a list of such medications can be found at https://crediblemeds.org/
7. Poorly controlled diabetes mellitus, defined as HgB A1c >9%
8. Known glucose-6-phosphate dehydrogenase (G6PD) deficiency
9. Use of quinolone for 7 days within past 30 days
10. QT_c_ interval (Fridericia corrected) >450 msec for men and women at screening and randomization

*TB* tuberculosis, *ALT* alanine transaminase

Although pregnancy is not a contraindication to levofloxacin treatment for MDR-TB, the safety of levofloxacin use at investigational doses in pregnancy is unclear. Female study participants with child-bearing potential must therefore have a negative pregnancy test and agree to practice adequate birth control. Study participants who become pregnant during the trial period will have study treatment discontinued and replaced with the local standard for treatment of MDR-TB during pregnancy.

**Fig. 1:**
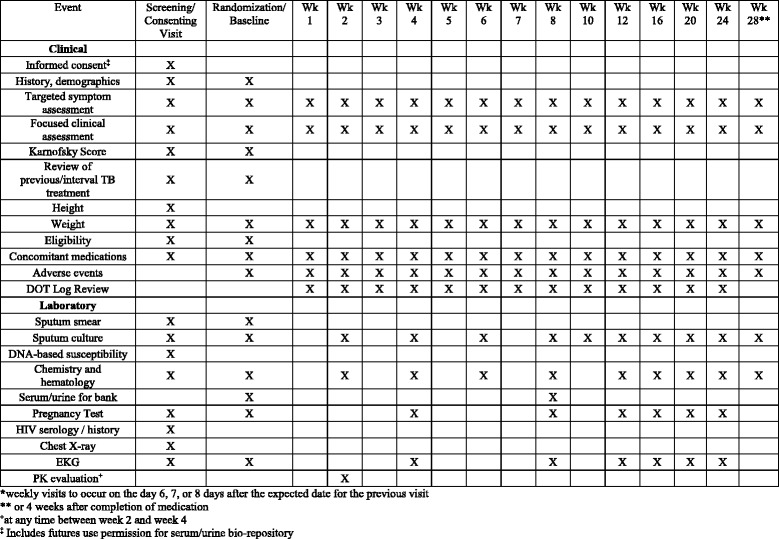
Standard Protocol Items: Recommendation For Interventional Trials (SPIRIT) figure

Laboratory parameters for inclusion are: creatinine clearance >50, hemoglobin >9.0 g/dL, platelet count >80,000/mm3, absolute neutrophil count >1000/mm3, known CD4 count within 6 months, serum alanine transaminase (ALT) and total bilirubin less than three times the upper limit of normal. Participants will later be excluded if they have one or more of the following: negative culture at screening and baseline, phenotypic susceptibility to isoniazid and rifampin; or phenotypic resistance to ofloxacin. The study procedures and assessments are outlined in the Standard Protocol Items: Recommendation For Interventional Trials (SPIRIT) checklist (see Additional file 1) and SPIRIT Figure (Fig. 1).

### Recruitment process

Potentially eligible patients are invited to participate. The risks and benefits of study participation are explained in detail and informed consent is obtained. At the initial screening, eligibility is assessed and information is collected on duration of tuberculosis, known duration of MDR-TB, number and duration of previous episodes of tuberculosis treatment, extent of disease (radiographic), height, weight, age, sex, HIV status, CD4 count if HIV-infected, comorbid conditions, prior drug susceptibility results, blood urea nitrogen (BUN), creatinine, ALT, bilirubin, Karnofsky score and concurrent medications.

A randomization eligibility form is then filled out and submitted to the study data center, which then certifies that the patient is eligible for randomization. The patient is assigned a study treatment arm if eligible or referred to the local source of TB care if not.

### Treatment allocation

Participants are randomized to one of the four treatment arms in the ratio 1:1:1:1. Patients are randomized using pre-prepared lists and blocks of varying sizes, with separate lists prepared for each site. Randomization is stratified by site (three sites) and, in South Africa, by HIV status to control for the difference in incidence of HIV co-infection in South Africa compared to Peru, 63% to 1.7%, respectively [15]. The randomization list and corresponding envelopes containing randomization assignments are prepared by a statistician independent to the study. The envelopes are stored in the study pharmacy in each country (one pharmacy for two sites in Lima) and opened by the pharmacy teams. Assignment is emailed to the study statistician who verifies each allocation.

### Study regimens

**Fig. 2:**
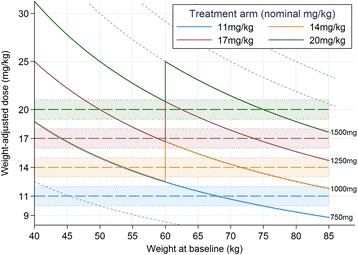
Relationship between weight-banded dosing and expected mg/kg effective dose

Patients are randomized to the study with MDR-TB to OBR plus levofloxacin at one of four doses: 11 mg/kg/day, 14 mg/kg/day, 17 mg/kg/day or 20 mg/kg/day. Weight banding results in corresponding doses of 750 mg, 1000 mg, 1250 mg and 1500 mg daily, respectively (Table 2) for patients who weigh at least 60 kg. The relationship between these weight-banded doses and the target doses in milligrams/kilogram is shown in Fig. 2. Therefore, all participants receive at least the World Health Organization (WHO) high-dose recommendation of 750 mg/day [2].

**Table 2:** Dosing of levofloxacin (LFX) by treatment arm and weight at randomization

Weight band	11 mg/kg		14 mg/kg		17 mg/kg		20 mg/kg	
L = 250 mg LFX P = 250 mg placebo	# LFX + placebo	Total LFX (mg)	# LFX + placebo	Total LFX(mg)	# LFX + placebo	Total LFX (mg)	# LFX + placebo	Total LFX (mg)
<60 kg	3 L + 3P	750	3 L + 3P	750	4 L + 2P	1000	5 L + 1P	1250
> = 60 kg	3 L + 3P	750	4 L + 2P	1000	5 L + 1P	1250	6 L + 0P	1500

This is a placebo-controlled study; all participants receive the same number of pills with varying proportions of active drug and placebo to ensure that patients and clinicians are not aware of the allocated treatment arm. Only the pharmacist and the study statistician are aware of the arm to which a participant has been allocated. Their unblinding permits correct treatment dispensation and oversight of the randomization process, respectively.

The OBR is selected at the discretion of the local investigators, in order to conform to local standards of care and guidelines. The OBR drugs are procured through the site’s routine procurement mechanism. At the start of the study, the empiric MDR-TB regimen in Lima included an FQ plus kanamycin, pyrazinamide, ethambutol and cycloserine. In Cape Town terizidone was regularly used, while ethambutol and cycloserine were not. In both sites, if sensitivity cannot be confirmed or there has been prior exposure to drugs included in the empiric regimen, additional drugs are added to the levofloxacin to ensure at least four likely effective drugs, plus pyrazinamide (PZA), per WHO recommendations [2].

Experimental doses are delivered daily for 6 months. After the end of the study treatment, MDR therapy is continued per the locally used MDR-TB regimen for approximately 18 additional months.

### Blinding to treatment assignment

All study staff (except the pharmacist and statistician) and study participants are blinded to the assigned treatment. The treatment allocation of all participants will not be unblinded until all randomized participants have finished study therapy (minimum 168 doses) plus 4 weeks of follow up after the last dose of study therapy. Individual participant allocation may be unblinded, if, in the opinion of the study investigator, and concurrence with the protocol team, the definitive attribution of the adverse event to the study drug will benefit the management of the event.

### Pharmacokinetic sampling

Pharmacokinetic sampling is performed in one 24-hour period between the 14th day through the 28th day from the start of the study regimen. Study drugs are swallowed in the morning with 200 cc of water after having nothing by mouth (NPO) for 8 hours and no food is ingested for the next 2 hours. Venous blood (5 ml) is collected for determination of levofloxacin plasma concentrations before dosing (time 0) and at 1, 2, 4, 8, 12 and 24 hours after administration of the morning dose of study drug treatment on one day between days 14 and 28 of treatment. Participants will have received a minimum of three consecutive daily doses of study drugs prior to the blood collections.

Participants are then interviewed to obtain additional information about medical and social history, recent weight loss or weight gain, concomitant medications on the day prior to and on the days of pharmacokinetic sampling, gastrointestinal symptoms, and timing of meals, snacks and medications relative to study drug dosing.

### Assessment of study endpoints and duration of follow-up

The primary efficacy endpoint is time to negative culture on solid medium. Specifically, this is defined as the time from initiation of study treatment to the first of two successive negative cultures (without an intervening positive culture) that are not followed by a culture-positive specimen within 28 weeks of treatment initiation. In determining time to sputum culture conversion, unevaluable cultures will be ignored. Sputum cultures are collected bi-weekly for 12 weeks, then every 4 weeks through 24 weeks of treatment. Participants unable to produce a specimen for sputum culture despite repeated attempts are considered to have had a negative culture on that date.

The primary safety endpoint is the number of adverse events (AEs) of grade 3 or higher occurring up to and including the time on study drug plus 4 weeks post study drug completion. Grading is performed in accordance with the Common Terminology Criteria for Adverse Events v3.0 (CTCAE) published on 9 August 2006 or the most updated version. Adverse events are classified by investigators as related or not related to levofloxacin. The endpoint, however, is the absolute number of events, irrespective of relatedness. The primary endpoint for the analysis of tolerability will be the ability to complete 24 weeks of treatment with the assigned levofloxacin dose, defined as the receipt of 168 daily doses of assigned study drug dose within 200 days of initiation of study regimen.

### Sample size assumptions

For the primary efficacy endpoint, response estimate was based on a study of patients with MDR-TB in Latvia, which found that 50% of patients with MDR-TB experienced sputum-culture conversion, measured on solid medium, by 2 months and 75% by 3 months [16]. Since these patients had received earlier-generation FQ, we assumed that the response in the control arm (that containing OBR plus 750 mg of levofloxacin) would be equivalent to that observed in the Latvian study. Using the Fisher transformation of the correlation coefficient, we estimated that 62 evaluable patients would allow detection of moderate correlation (-0.40) between AUC/MIC and time to sputum-culture conversion with 90% power and two-sided significance of 5%.

The power to assess tolerability was estimated using the normal approximation formulas given in Hsieh et al. [17]. A second sample size calculation was completed based on the logistic regression of a binary response variable (whether the participant has completed 6 months of treatment with the assigned dose or not) on a continuous, normally distributed variable (AUC). The estimated mean AUC will be 140 μg-h/ml, with standard deviation of about 65 μg-h/ml, [14] giving a total of 79 evaluable patients needed to detect an OR of 0.412 between the proportion completing treatment at the mean AUC and the proportion completing treatment at the mean + 1 SD AUC (205 μg-h/ml).

For the toxicity endpoint, we estimated the power to detect a relationship between the AUC and frequency of grade 3, 4 or 5 AEs, occurring up to and including the time on study drug plus 4 weeks post study drug completion. With 79 evaluable participants, there will be 80% power to detect an association if the proportion with AEs grade 3 or higher at mean AUC + 1 SD is 46% (p2) and 90% power if the proportion is 49%.

### Analysis populations

The primary analyses will be conducted on the as-allocated, intention to treat (ITT) and modified intention to treat (MITT) analysis populations. The ITT analysis population includes all randomized participants who received at least one dose of trial medication. The MITT analysis population is the same with the exclusion of participants determined to have had a negative culture at screening and baseline, phenotypic susceptibility to isoniazid or rifampin, or phenotypic resistance to ofloxacin that was not detected by baseline molecular susceptibility testing. Participants who complete study drug treatment in the defined window of 200 days from initiation will be considered to have completed the study regimen per protocol.

### Analysis plan

#### Objective 1: determine the levofloxacin AUC/MIC that provides the shortest time to sputum culture conversion on solid medium

It is expected that time to culture conversion follows a log-normal distribution and therefore times will be log-transformed [16]. The participant’s levofloxacin (AUC) will be assessed by pharmacokinetic measurements; this, divided by the MIC of levofloxacin required to kill 90% of the participant’s isolate (AUC/MIC_90_) will be the predictor variable. The results will be adjusted for creatinine and HIV status, presented both as unadjusted and adjusted analyses. Linear regression will be used to calculate the Pearson correlation coefficient between AUC/MIC and log-transformed time to culture conversion. The relationship will be plotted on a scatter plot of AUC/MIC against time to culture-conversion curve on the log scale. The primary objective is to determine whether there is sufficient evidence to reject the null hypothesis that the Pearson correlation coefficient is equal to zero.

#### Objective 2: determine the highest levofloxacin AUC that is both safe and associated with fewer than 25% of patients discontinuing or reducing their dose of levofloxacin

The number of adverse events grade 3 or higher will be plotted against the AUC of the individual in whom those events occurred. Linear regression will be used to calculate the Pearson correlation coefficient between AUC and number and severity of events. If the distribution of the AUC is skewed, then the AUC will be log transformed. Other transformations will be explored as appropriate. The resulting relationship will be used to identify an AUC at which more than 25% of participants would be expected to have grade 3, 4, or 5 adverse events. Time-to-event methods will be used to compare the incidence of AEs between dosing groups and to evaluate whether AEs tend to accumulate earlier when higher doses of levofloxacin are taken. The safety analysis will be repeated, considering only the number of grade 3, 4 and 5 adverse events that were considered to be possibly, probably or definitely related to study medication.

For tolerability, logistic regression will be used to model the association between these variables with the AUC included as a continuous covariate. If the distribution of the AUC is skewed, then the AUC will be log transformed. Other transformations will be explored as appropriate. The primary objective is to determine whether there is sufficient evidence to reject the null hypothesis that the odds ratio of discontinuation for a unit increase in the AUC is equal to 1. Model parameters will also be used to estimate the AUC associated with 25% intolerability. This primary analysis will exclude participants withdrawn from treatment where the reason was not definitely, probably or possibly related to study medication.

### Dissemination of trial findings

Following completion of the study, the investigators plan to publish the results of this research in a scientific journal. The International Committee of Medical Journal Editors (ICMJE) member journals have adopted a trials-registration policy as a condition for publication. This trial was registered at ClinicalTrials.gov before patient enrollment was initiated (ClinicalTrials.gov identifier, NCT01918397). Once primary analysis is complete the data and de-identified dataset will be provided for public use on the Tuberculosis Trials Consortium website.

## Discussion

Opti-Q is a novel, multisite study to optimize dosing of FQ, one of the cornerstones of MDR-TB treatment. The study design and implementation incorporate multiple innovations. First, it is the first study conducted through collaboration between two US publicly funded trial networks. This hybrid serves as a blueprint for other studies now being undertaken jointly by the US TBTC and AIDS Clinical Trials Group. This feature is critical for the continued improvement of treatment for MDR-TB, which will require a sustained and robust series of investigations.

Second, Opti-Q uses a variation of the stepwise approach to dose-escalation evaluation. Studies of increasing drug dose to evaluate safety of higher doses are often performed in a stepwise fashion, such that one dose is studied and evaluated in a single group of patients before making a decision to enroll patients in the next-highest dose. We designed Opti-Q to be significantly more efficient than a dose-escalation study, which would have required roughly 200 evaluable patients and would have likely needed 5–6 years to complete. By concurrently testing doses of 11, 14, 17, and 20 mg/kg/day, we were able to reduce the sample size by more than half. Since doses of levofloxacin up to 20 mg/kg/day have been previously used and well-tolerated, there was equipoise about the risk and benefits of doses up to 20 mg/kg. The smaller number of patients exposed to higher doses, reduced time to urgently needed guidance on levofloxacin dose optimization, and decreased cost all contribute to the balance in favor of this design.

Third, studying the AUC and AUC/MIC rather than dose is also more efficient. Comparing each single dose with the next requires a large sample size for each dose group, because there is substantial variability in the AUC/MIC achieved. On the other hand, comparing one continuous variable (AUC/MIC) with a continuous outcome (time to sputum culture conversion) takes advantage of the AUC/MIC variability resulting from a given dose to provide increased precision in establishing the relationship. As a result, the target sample size is somewhat smaller.

Fourth, by identifying target AUC and AUC/MIC ratios, and by increasing our understanding of their relationship to levofloxacin dose, this study will allow physicians to treat patients with MDR-TB with the highest doses of levofloxacin that are associated with acceptable tolerability. This knowledge will facilitate the levofloxacin dosing that is likely to lead to the target AUC (and AUC/MIC of the expected MIC) for individual patients. Complementary use of therapeutic drug monitoring could validate these results and provide further refinement of the covariates that predict target AUC in different populations. Moving the field of tuberculosis treatment to an outcome-based dosing paradigm could potentially greatly increase the efficacy of current and future tuberculosis treatment regimens. This is especially important in treating MDR-TB, where current regimens are suboptimal.

Finally, the OBR methodology applied in this study provides a pragmatic solution to lingering questions about the dose of a single drug within a multidrug regimen. The design permits broad inclusion, resulting in more heterogeneous study populations and consequently more confidence about the generalizability of the results. The variability in the background measure reflects differences in participant characteristics that are expected to be relatively evenly distributed among the treatment groups. We recognize that identification of the optimal target AUC and AUC/MIC for levofloxacin will not translate directly into practice in settings that do not have access to pharmacokinetic monitoring. Therefore, we also plan to model levofloxacin dosing using our data in order to provide dosing guidance to clinicians to facilitate optimal dosing. Thus, clinicians without access to levofloxacin concentrations will be able to make a reasonable estimate of the dose that is likely to maximize levofloxacin efficacy while minimizing levofloxacin toxicity.

In summary, this study will provide essential information to guide dosing of levofloxacin for the treatment of MDR-TB; such dose optimization has not previously been performed, due largely to the fact that FQs were developed for treatment of other infectious diseases and only later applied to TB treatment regimens. Second, this study will identify the optimal doses for use in combination with other anti-TB agents. Optimization of TB treatment agents has previously been performed only with single agents, not with agents in the context of other companion drugs. Since this is how TB drugs are used in practice, this investigative strategy should have wide applicability to future studies of TB drug dosing. For more information on the study protocol see Additional file 2.

### Trial status

Recruitment began in December 2014 and it is anticipated that Opt-Q completed enrollment in January of 2017.

## Additional files

Additional file 1: SPIRIT checklist. This document provides an outline of the research and methods of the study protocol per SPIRIT guidelines. (DOC 122 kb)

Additional file 2: OPTI-Q Study protocol version 1.6. This document provides the full protocol for the OPTI-Q Study as of 21 January 2014. (DOCX 323 kb)

Additional file 3: Ethical approval reference numbers. This document contains a list of the specific names and reference numbers for all ethical bodies that approved the study in the various participating and recruiting centers involved. (PDF 207 kb)

Additional file 4: OPTIQ Informed Consent Form. This document is the approved consent form used to enroll eligible patients into the OPTI-Q study. (PDF 260 kb)

## Abbreviations

AE: Adverse events
ALT: Alanine transaminase
AUC: Area under the curve
AUC/MIC: Area under the curve to minimum inhibitory concentration ratio
FQ: Fluoroquinolone
HIV: Human immunodeficiency virus
ITT: Intention to treat
MDR-TB: Multidrug-resistant tuberculosis
MIC: Minimum inhibitory concentration
MITT: Modified intention to treat
NIH: National Institutes of Health
OBR: Optimized background regimen
SAEs: Serious adverse events (grades 3, 4 and 5)
TBTC: Tuberculosis Trials Consortium
WHO: World Health Organization
