# Supplementary material for: An optimized background regimen design to evaluate the contribution of levofloxacin to multidrug-resistant tuberculosis treatment regimens: study protocol for a randomized controlled trial
Source: Trials. 2017 Nov 25;18:563. doi: 10.1186/s13063-017-2292-x (PMC5702225; doi:10.1186/s13063-017-2292-x)
Supplement: Supplementary file 2 — OPTI-Q Study protocol version 1.6. This document provides the full protocol for the OPTI-Q Study as of 21 January 2014. (DOCX 323 kb) [file 13063_2017_2292_MOESM2_ESM.docx]

Additional file 2

Prospective, Randomized, Blinded Phase 2 Pharmacokinetic/Pharmacodynamic Study of the Efficacy and Tolerability of Levofloxacin in Combination with Optimized Background Regimen (OBR) for the treatment of MDR-TB

Acronym: “Opti-Q Study”

DMID Funding Mechanism: U01

Pharmaceutical Support Provided by: MacLeods Pharmaceuticals, Mumbai, India

Other Identifying Numbers: CDC TBTC Study 32

IND Sponsor: MacLeods Pharmaceuticals, Mumbai, India

(IND#115872)

Principal Investigator: C. Robert Horsburgh, Jr, MD, Boston University, Boston MA

DMID Representative: Mamodikoe Makhene, MD, MPH

Version Number: 1.6

Date: 21 January 2014

Statement of Compliance

This study will be carried out in accordance with Good Clinical Practice (GCP) as required by:

- United States (US) Code of Federal Regulations (CFR) applicable to clinical studies (45 CFR Part 46; 21 CFR Part 50, 21 CFR Part 56, and 21 CFR Part 312)
- ICH E6; 62 Federal Register 25691 (1997)
- NIH Clinical Terms of Award

All key personnel (all individuals responsible for the design and conduct of this study) have completed Human Subjects Protection Training.

Signature Page

The signature below constitutes the approval of this protocol and the attachments, and provides the necessary assurances that this trial will be conducted according to all stipulations of the protocol, including all statements regarding confidentiality, and according to local legal and regulatory requirements and applicable US federal regulations and ICH guidelines.

| Site Investigator:* | | | |
| --- | --- | --- | --- |
| Signed: |  | Date: |  |
|  | *Name*  *Title* |  |  |

** The protocol should be signed by the local investigator who is responsible for the study implementation at his/her specific site; i.e., if Investigational New Drug study, the individual who signs the Form FDA 1572.*

Table of Contents

Page

[Statement of Compliance ii](#_Toc375315839)

[Signature Page iii](#_Toc375315840)

[Table of Contents iv](#_Toc375315841)

[List of Abbreviations vii](#_Toc375315842)

[Protocol Summary ix](#_Toc375315843)

[1 Key Roles 1](#_Toc375315844)

[2 Background Information and Scientific Rationale 6](#_Toc375315845)

[2.1 Background Information 6](#_Toc375315846)

[2.1.1. MDR-TB Epidemiology 6](#_Toc375315847)

[2.1.2. State of MDR-TB treatment 6](#_Toc375315848)

[2.1.3. Efficacy of Fluoroquinolones for TB treatment 7](#_Toc375315849)

[2.1.4. Other new drugs for treatment of MDR-TB 8](#_Toc375315850)

[2.1.5. Why levofloxacin will be the drug of choice for treatment of MDR-TB 8](#_Toc375315851)

[2.1.6. Currently used levofloxacin doses are suboptimal 9](#_Toc375315852)

[2.1.7. Emergence of fluoroquinolone resistance 9](#_Toc375315853)

[2.1.8. Pharmacokinetic and pharmacodynamic parameters of levofloxacin 10](#_Toc375315854)

[2.1.9. Assessing Response to MDR-TB treatment 10](#_Toc375315855)

[2.1.10. Alternative Study Designs 11](#_Toc375315856)

[2.2 Scientific Rationale 11](#_Toc375315857)

[2.3 Potential Risks and Benefits 13](#_Toc375315858)

[2.3.1 Potential Risks 13](#_Toc375315859)

[2.3.2 Known Potential Benefits 18](#_Toc375315860)

[3 Objectives 20](#_Toc375315861)

[3.1 Study Objectives 20](#_Toc375315862)

[3.2 Study Outcome Measures 20](#_Toc375315863)

[3.2.1 Primary Outcome Measures 20](#_Toc375315864)

[3.2.2 Secondary Outcome Measures 21](#_Toc375315865)

[4 Study Design 22](#_Toc375315866)

[4.1 Study Population 22](#_Toc375315867)

[4.2 Treatment delivery and duration 22](#_Toc375315868)

[4.3 Assessment of study objectives and measurement of endpoints 23](#_Toc375315869)

[4.4 Optimized Background Regimen (OBR) 23](#_Toc375315870)

[5 Study Enrollment and Withdrawal 25](#_Toc375315871)

[5.1 Randomization Inclusion Criteria 25](#_Toc375315872)

[5.2 Randomization Exclusion Criteria 26](#_Toc375315873)

[5.3 Treatment Assignment Procedures 26](#_Toc375315874)

[5.3.1 Randomization Procedures 26](#_Toc375315875)

[5.3.2 Masking Procedures 26](#_Toc375315876)

[5.3.3 Reasons for Withdrawal of Study Drug 27](#_Toc375315877)

[5.3.4 Handling of Patients whose Study Drug is Withdrawn 27](#_Toc375315878)

[5.3.5 Termination of Study 28](#_Toc375315879)

[6 Study Intervention/Investigational Product 29](#_Toc375315880)

[6.1 Study Product Description 29](#_Toc375315881)

[6.1.1 Acquisition 30](#_Toc375315882)

[6.1.2 Formulation, Packaging, and Labeling 30](#_Toc375315883)

[6.1.3 Product Storage and Stability 30](#_Toc375315884)

[6.2 Dosage, Preparation and Administration of Study Intervention/Investigational Product 30](#_Toc375315885)

[6.3 Modification of Study Intervention/Investigational Product for a Participant 31](#_Toc375315886)

[6.4 Accountability Procedures for the Study Intervention/Investigational Product(s) 32](#_Toc375315887)

[6.5 Assessment of Subject Compliance with Study Intervention/Investigational Product 32](#_Toc375315888)

[6.6 Concomitant Medications/Treatments 32](#_Toc375315889)

[6.7 OBR (Companion Drugs) 32](#_Toc375315890)

[7 Study Schedule 34](#_Toc375315891)

[7.1 Screening / Consenting Visit 34](#_Toc375315892)

[7.2 Randomization/Baseline 34](#_Toc375315893)

[7.3 Follow-up 35](#_Toc375315894)

[7.4 Final Study Visit 40](#_Toc375315895)

[7.5 Early Termination Visit 40](#_Toc375315896)

[7.6 Unscheduled Visit 40](#_Toc375315897)

[8 Study Procedures/Evaluations 41](#_Toc375315898)

[8.1 Clinical Evaluations 41](#_Toc375315899)

[8.2 Laboratory Evaluations 42](#_Toc375315900)

[8.2.1 Clinical Laboratory Evaluations 42](#_Toc375315901)

[8.2.2 Special Assays or Procedures 43](#_Toc375315902)

[8.2.3 Specimen Preparation, Handling, and Shipping 44](#_Toc375315903)

[9 Assessment of Safety 45](#_Toc375315904)

[9.1 Specification of Safety Parameters 45](#_Toc375315905)

[9.2 Methods and Timing for Assessing, Recording, and Analyzing Safety Parameters 45](#_Toc375315906)

[9.2.1 Adverse Events 45](#_Toc375315907)

[9.2.2 Procedures to be Followed in the Event of Abnormal Laboratory Test Values or Abnormal Clinical Findings 48](#_Toc375315908)

[9.3 Reporting Procedures 48](#_Toc375315909)

[9.3.1 Serious Adverse Events 49](#_Toc375315910)

[9.3.2 Regulatory Reporting for Studies Not Conducted Under DMID‑Sponsored IND 49](#_Toc375315911)

[9.3.3 Reporting of Pregnancy 50](#_Toc375315912)

[9.4 Type and Duration of Follow-up of Subjects after Adverse Events 50](#_Toc375315913)

[9.5 Guidance for Temporary Discontinuation of Study Drug 50](#_Toc375315914)

[9.6 Safety Oversight 50](#_Toc375315915)

[9.6.1 Data and Safety Monitoring Board (DSMB) 50](#_Toc375315916)

[9.6.2 Independent Safety Monitor (ISM) 50](#_Toc375315917)

[9.6.3 Halting Rules 51](#_Toc375315918)

[10 Clinical Monitoring 52](#_Toc375315919)

[10.1 Site Monitoring Plan 52](#_Toc375315920)

[11 Statistical Considerations 53](#_Toc375315921)

[11.1 Study Hypotheses 53](#_Toc375315922)

[11.2 Sample Size Considerations 53](#_Toc375315923)

[11.3 Planned Interim Analyses (if applicable) 57](#_Toc375315924)

[11.4 Final Analysis Plan 57](#_Toc375315925)

[12 Source Documents and Access to Source Data/Documents 61](#_Toc375315926)

[13 Quality Control and Quality Assurance 62](#_Toc375315927)

[14 Ethics/Protection of Human Subjects 63](#_Toc375315928)

[14.1 Ethical Standard 63](#_Toc375315929)

[14.2 Institutional Review Board 63](#_Toc375315930)

[14.3 Informed Consent Process 63](#_Toc375315931)

[14.4 Informed Consent/Assent Process (in Case of a Minor) 64](#_Toc375315932)

[14.5 Exclusion of Women, Minorities, and Children (Special Populations) 64](#_Toc375315933)

[14.6 Subject Confidentiality 65](#_Toc375315934)

[14.7 Study Discontinuation 65](#_Toc375315935)

[14.8 Future Use of Stored Specimens 65](#_Toc375315936)

[15 Data Handling and Record Keeping 67](#_Toc375315937)

[15.1 Data Management Responsibilities 67](#_Toc375315938)

[15.2 Data Capture Methods 68](#_Toc375315939)

[15.3 Types of Data 68](#_Toc375315940)

[15.4 Timing/Reports 68](#_Toc375315941)

[15.5 Study Records Retention 68](#_Toc375315942)

[15.6 Protocol Deviations 68](#_Toc375315943)

[16 Publication Policy 70](#_Toc375315944)

[17 Literature References 71](#_Toc375315945)

[APPENDIX A: SCHEDULE OF EVENTS* 76](#_Toc375315946)

[APPENDIX B: KARNOFSKY SCALE 77](#_Toc375315947)

[APPENDIX C: TOXICITY MANAGEMENT ALGORITHM 79](#_Toc375315948)

List of Abbreviations

| AE | Adverse Event/Adverse Experience |
| --- | --- |
| ALT | Alanine Aminotransferase |
| ANC | Absolute neutrophil count |
| AUC | Area Under the Curve (24 hours) |
| CDC | Centers for Disease Control and Prevention |
| CFR | Code of Federal Regulations |
| CIOMS | Council for International Organizations of Medical Sciences |
| CONSORT | Consolidated Standards of Reporting Trials |
| CPX | Ciprofloxacin |
| CRF  DCC | Case Report Form  Data Coordinating Center |
| DMID | Division of Microbiology and Infectious Diseases, NIAID, NIH, DHHS |
| DOT | Directly Observed Therapy |
| DSMB | Data and Safety Monitoring Board |
| eCRF | Electronic Case Report Form |
| EBA | Early Bactericidal Activity |
| FDA | Food and Drug Administration |
| FQ | Fluoroquinolone |
| FTP | File Transfer Protocol |
| GFX | Gatifloxacin |
| IB | Investigator’s Brochure |
| ICF | Informed Consent Form |
| IND | Investigational New Drug Application |
| IRB | Institutional Review Board |
| ISM | Independent Safety Monitor |
| ITT | Intention To Treat |
| LFX | Levofloxacin |
| MDR-TB | Multidrug-resistant tuberculosis |
| MIC | Minimum Inhibitory Concentration |
| MFX | Moxifloxacin |
| MOP | Manual of Procedures |
| MPC | Mutant Prevention Concentration |
| N | Number (typically refers to subjects) |
| NIH | National Institutes of Health |
| OAT | Organic Ion Transport |
| OBR | Optimized Background Regimen |
| OCRA | Office of Clinical Research Affairs, DMID, NIAID, NIH, DHHS |
| OCT | Organic Cation Transport |
| OFX | Ofloxacin |
| OHRP | Office for Human Research Protections |
| OHSR | Office for Human Subjects Research |
| ORA | Office of Regulatory Affairs, DMID, NIAID, NIH, DHHS |
| PI | Principal Investigator |
| PK | Pharmacokinetics |
| PZA | Pyrazinamide |
| SAE | Serious Adverse Event/ Serious Adverse Experience |
| SD | Standard Deviation |
| SOPSSCC | Standard Operating Procedure Serial Sputum Colony Counts |
| TB | Tuberculosis |
| TTD | Time to Detection |
| US | United States |
| WHO | World Health Organization |
| XDR-TB | Extensively Drug Resistant Tuberculosis |

Protocol Summary

| **Title:** | Prospective, Randomized, Blinded Phase II Pharmacokinetic/Pharmacodynamic Study of the Efficacy and Tolerability of Levofloxacin in Combination with Optimized Background Regimen for the Treatment of MDR-TB |
| --- | --- |
| **Phase:** | II |
| **Population:** | The proposed study will enroll adults with pulmonary MDR-TB with sputum that is isoniazid and rifampin resistant by MTBDRplus and fluoroquinolone susceptible by MTBDRsl at sites in Peru and South Africa. |
| **Number of Sites:** | Three |
| **Study Duration:** | Four years. |
| **Subject Participation Duration:** | Enrolled patients will have directly observed therapy (DOT) every day for 24 weeks (168 doses). Final study visit will occur at 28 weeks. |
| **Description of Agent or Intervention:** | Randomize patients with MDR-TB to 11, 14, 17 or 20 mg/Kg daily of levofloxacin in combination with optimized background regimen (OBR). OBR in this protocol is defined as an **optimized regimen to accompany, but not including, a quinolone.** |
| **Objectives:** | Primary:   1. Determine the levofloxacin AUC/MIC that provides the shortest time to sputum culture conversion on solid medium. 2. Determine the highest levofloxacin AUC that is both safe and associated with fewer than 25% of patients discontinuing or reducing their dose of levofloxacin. 3. Develop a dosing algorithm to achieve the AUC associated with maximal efficacy and acceptable safety and tolerability.   Secondary:   1. Determine the levofloxacin AUC/MIC that provides the shortest time to sputum culture conversion in liquid medium. 2. Determine if baseline PZA susceptibility is associated with shorter time to sputum culture conversion after controlling for levofloxacin AUC 3. Describe the differences in safety and efficacy between assigned study dose groups. |
| **Description of Study Design:** | - - Subjects: Adult patients with pulmonary TB, whose sputum contains isoniazid and rifampin resistant, ofloxacin susceptible *M. tuberculosis*, HIV positive (with or without antiretroviral therapy) or negative (but not unknown), weight >40kg, age > 18 years, Karnofsky score of > 60 - 100 patients - Information to collect at enrollment: duration of TB, known duration of MDR-TB, number and duration of previous episodes of TB treatment, extent of disease (radiographic), height, weight, age, sex, HIV status, CD4 count if HIV-infected, co-morbid conditions, prior drug susceptibility results, BUN, creatinine, ALT, bilirubin, concurrent medications. - Randomized 1:1:1:1 to treatment with levofloxacin , 4 doses (all plus OBR):   Dose 1 - 11 mg/kg (<60 kg= 750 mg [3x 250 mg tablets]; 60+ kg= 750 mg [3x 250 mg tablets])  Dose 2 - 14 mg/kg (<60 kg= 750 mg [3x 250 mg tablets]; 60+ kg= 1000 mg [4x 250 mg tablets])  Dose 3 - 17 mg/kg (<60 kg= 1000 mg [4x 250 mg tablets]; 60+ kg= 1250 mg [5x 250 mg tablets])  Dose 4 - 20 mg/kg (<60 kg= 1250 mg [5 250 mg tablets]; 60+ kg= 1500 mg [6x 250 mg tablets])   - Blinded, controlled (all subjects take 6 tablets; single daily dose) - All doses directly administered and observed (levofloxacin and companion drugs) - Bi-weekly sputum cultures on 7H11 and in MGIT for 12 weeks, then every 4 weeks up to 24 weeks of treatment - Intensive PK (6 samples), conducted during a 24 hour period between day 14 through day 28 - Primary exposure variable for efficacy: AUC/MIC - Primary exposure variable for tolerability: AUC - Primary exposure for toxicity: Number of Grade 3,4,or 5 AEs - Primary exposure variable for development of algorithm: Dose of levofloxacin |
| **Estimated Time to Complete Enrollment:** | 24 months from start of enrollment |

**Schematic of Study Design**:

Screen subjects, ensure eligibility and obtain informed consent.

Enrollment and Randomization (1:1:1:1)

Day 0

Supervised spot sputum collection; Assess for safety and tolerability; serum and urine biomarker

**Study phase**

Day 7

Assess for safety and tolerability

Day 14

Early morning sputum collection; PK*; Assess for safety and tolerability

Day 168

Day 140

Day 112

Day 84

Day 56

Day 49

Day 42

Day 35

Day 28

Day 21

* PK once between days 14 and 28

**Assessment of Final Study Outcome Measures**

Early morning Sputum collection; Assess for safety and tolerability

Early morning Sputum collection; Assess for safety and tolerability

Early morning Sputum collection; Assess for safety and tolerability

Assess for safety and tolerability

Early morning Sputum collection; Assess for safety and tolerability; serum and urine biomarker

Assess for safety and tolerability

Early morning sputum collection; Assess for safety and tolerability

Assess for safety and tolerability

Early morning sputum collection; Assess for safety and tolerability

Assess for safety and tolerability

# Key Roles

| **Individuals:** |  |
| --- | --- |
|  | **Principal Investigator:**  C. Robert Horsburgh, Jr., MD, MUS  Department of Epidemiology  Boston University School of Public Health  715 Albany Street, T3E  Boston MA USA 02118  Email: rhorsbu@bu.edu  **Co-Investigators:**  Andreas Diacon, MD  Task Applied Science  Brooklyn Chest Hospital Cape Town, South Africa E-mail*:* ahd@sun.ac.za  Nancy Dianis, MS  Westat, Inc.  1600 Research Boulevard  Rockville, MD 20850  Tel: 301.738.8379  Email: [nancydianis@westat.com](mailto:nancydianis@westat.com)  Kathleen Eisenach, PhD  University of Arkansas for Medical Sciences  4301 W. Markham, Slot 845  Biomedical Research Center II, Rm 607-2  Little Rock, AR 72205  Tele (501) 526-6966  FAX  (501) 526-6886  E-mail: EisenachKathleenD@uams.edu  Eduardo Gotuzzo, MD  UniversityofCayetanaHeredia  Lima, Peru E-mail: eduardo.gotuzzo@upch.pe  Leonid Lecca, MD  SociosEnSalud Sucursal Peru/ Partners In Health Avenida Merino, Reyna 575, Carabayllo, Lima 6, Peru Llecca_SES@pih.org  William Mac Kenzie, MD  Centers for Disease Control and Prevention  Division of TB Elimination 1600 Clifton Road, MS E-10  E-mail: william.mackenzie@cdc.hhs.gov  Beverly Metchock, PhD  Centers for Disease Control and Prevention  Division of TB Eliminati*on* 1600 Clifton Road, MS F-08  E-mail: bem1@cdc.gov  Carole Mitnick, DSc  Harvard Medical School 641 Huntington Avenue, Boston, MA 02115  +1-617-432-6018 +1-617-432-2565 carole_mitnick@hms.harvard.edu  Charles Peloquin, Pharm. D  University of Florida  1600 SW Archer Road, Rm P4-33  Gainesville, FL 32610  Tel.: 352.273.6266  Email: [peloquin@cop.ufl.edu](mailto:peloquin@cop.ufl.edu)  Patrick Phillips, PhD  MRC Clinical Trials Unit  125 Kingsway  London WC2B 6NH  Tel: +44.0.20.7670.4637  Email: [ppp@ctu.mrc.ac.uk](mailto:ppp@ctu.mrc.ac.uk) |
|  |  |
| **Institutions:** | Clinical enrollment sites:  1. Task Biosciences at Brooklyn Chest Hospital, Cape Town, South Africa  2. Socios en Salud, Lima, Peru  3. University of Cayetana Heredia Hospital, Lima, Peru  Central Microbiology Laboratory:  Mycobacteriology Reference Laboratory  Centers for Disease Control and Prevention  1600 Clifton Road, Atlanta GA USA  30333  Central Clinical Pharmacology Laboratory: University of Florida, College of Pharmacy, 1600 SW Archer Rd. Room P4-33, Gainesville, FL USA 32610-0486  Tel: 352-273-6266  Fax: 352-273-6804  **Data Coordinating Center:**  Westat, Inc.  1600 Research Boulevard  Rockville, MD USA 20850-3129  **Industry Collaborator:**  Macleods Pharmaceuticals, Ltd, 3^rd^ floor, Atlanta Arcade, Church Road, Near Leela Hotel, Andheri-Kurla Road, Andheri (E), Mumbai – 400 059, India |
|  | **DMID Representative:**  Mamodikoe Makhene, MD, MPH  6610 Rockledge Drive, Room 3304  Bethesda MD 20892-6604  Tel 301-402-8544  Fax 301-496-8030  Email [Mamodikoe.Makhene@nih.hhs.gov](mailto:Mamodikoe.Makhene@nih.hhs.gov)  Mohamed (Mo) Elsafy, MD  DMID Medical Monitor  Tel: 301-451-3026  Cell: 301-204-3735  Email: [mo.elsafy@nih.gov](mailto:mo.elsafy@nih.gov)  Tena Knudsen, RN  DMID Clinical Project Manager  Tel: 301-451-3747  Fax: 301-496-8030  Email: knudsent@mail.nih.gov |
| **Institutional Review Boards** |  |

Institution Name: Boston University

Address: 560 Harrison Ave, Suite 300, Boston, MA 02118, USA

FWA Number: 00005790

Name of site PI: Charles Robert (Bob) Horsburgh, MD

Note: IRB approval will be obtained at this site

Institution Name: University of Florida

Address: PO Box 100173, Gainesville, FL 32610, USA

FWA Number: 00005790

Name of site PI: Charles Peloquin, PharmD

Note: Site is “not engaged”

Institution Name: Harvard University

Address: 90 Smith St, 3^rd^ Fl Rm 336, Boston, MA 02120, USA

FWA Number: FWA00007071

Name of site PI: Carole Mitnick, ScD

Note: Site is “not engaged”

Institution Name: University of Arkansas

Address: 4301 W. Markham St., Slot 636, Little Rock, AR 72205

FWA Number: 00001119

Name of site PI: Kathleen Eisenach, PhD

Note: Site is “not engaged”

Institution Name: University of California, San Francisco

Address: 3333 California Street, Suite 16, San Francisco, CA 94143, USA

FWA Number: 00000068

Name of site PI: Payam Nahid, MD

Note: Site is “not engaged”

Institution Name: Westat, Inc.

Address: 1600 Research Boulevard, Rockville, MD 20850-3129

FWA Number: 00005551

Name of site PI: Nancy Dianis, RN

Note: Site is “not engaged”

Institution Name: University College of London / MRC Clinical Trials

Address: University College London, Gower Street, London WC1E 6BT, UK

FWA Number: 00003159

Name of site PI: Patrick Phillips, PhD

Note: Site is “not engaged”

Institution Name: Centers for Disease Control and Prevention

Address: 1600 Clifton Rd, NE MS E-10, Atlanta, GA 30333, USA

FWA Number: 00001413

Name of site PI: William Mac Kenzie, MD

Note: Site is “not engaged”

Institution Name: Pharma Ethics Independent Research Ethics Committee

Address: 123 Amcor Road, Lyttelton Manor, Pretoria, South Africa

FWA Number: 000012241

Name of site PI: Andreas Diacon, MD

Note: IRB approval will be obtained at this site

Institution Name: ERB Universidad Peruana Cayetano Heredia

Address: Av. Honorio Delgado 430, Urb. Ingeniería, S.M.P. Lima, Peru.

FWA Number: 00000525

Name of site PI: Eduardo Gotuzzo, MD

Note: IRB approval will be obtained at this site

Institution Name: Comite Instrucional de Etica en Investigacion del Hospital Nacional Hipolito Unanue

Address: Av. Cesar Vallejo 1390, El Agustino, Lima, Peru

FWA Number: 00015265

Name of site PI: Leonid Lecca, MD

Note: IRB approval will be obtained at this site

**See Section 14.2 for definition of “engaged” and “not engaged”**

# Background Information and Scientific Rationale

## Background Information

### 2.1.1. MDR-TB Epidemiology

Approximately 5% of the 9 million new tuberculosis (TB) cases globally each year are caused by organisms that are resistant to isoniazid and rifampin, the two most important first-line anti-TB drugs, resulting in multidrug-resistant (MDR) TB.^1^MDR-TB is not distributed equally around the globe; rather it occurs in areas defined by WHO as “hot spots”. South Africa is estimated to have 10,000 new cases of MDR-TB each year, while Peru has 2,300.^1^

The situation is even worse when additional drug resistance is present, such as is seen in patients with *M. tuberculosis* isolates also resistant to fluoroquinolones and second-line injectable agents, known as XDR-TB. XDR-TB is estimated to occur in 5% of persons with MDR-TB and has been detected in 58 countries.^1^ Among patients with XDR-TB, only 44% have satisfactory outcomes, and 21% die..^2^ Patients with XDR-TB and HIV infection have even worse outcomes.^3^ In light of the potential severe implications of the existence and spread of MDR- and XDR-TB, particularly in high HIV-prevalence settings, improved outcomes among patients with MDR- are essential.

### 2.1.2. State of MDR-TB treatment

A recent review summarized the results of observational studies of MDR-TB and found that success is less common as a final treatment outcome than in the treatment of drug-susceptible TB, which results in 85-95% successful outcomes.^4^ Statistically significant risk factors in multivariate analysis, in addition to fluoroquinolone use, include HIV infection,^5-6^the number of drugs to which the patient’s pretreatment isolate is resistant,^7-9^quinolone resistance,^10-11^male sex,^12^previous treatment for MDR-TB^8, 11^younger age,^13^low BMI,^11, 14^cavitation^8, 15^and poor adherence to the regimen.^15^

Despite the use of specialized regimens for treatment, MDR-TB cure rates are substantially lower than those expected when treating drug-susceptible TB. Prior to the advent of the FQs, MDR-TB treatment was characterized by a cure rate of only 56%, with 37% five year mortality.^16^ Including an FQ in the regimen has led to improved overall cure rates to 61%-75% and ~20% mortality.^4, 10-11, 13-14^ Although there have been no controlled clinical trials of FQ use in treatment of MDR-TB, a number of observational studies have linked FQ use with good treatment outcomes.^15, 17-21^ Thus, FQ are an essential part of MDR-TB treatment regimens.

### 2.1.3. Efficacy of Fluoroquinolones for TB treatment

Fluoroquinolones target the DNA gyrase of bacteria, including that of Mycobacterium tuberculosis. The in vivo activity of the fluoroquinolones is concentration-dependent. Early members of this class, ofloxacin and ciprofloxacin, have demonstrable anti-tuberculosis activity and were essential to good outcomes in several observational studies of the treatment of MDR-TB.^15, 19-25^ Newer members of the fluoroquinolone class, levofloxacin (LFX), moxifloxacin (MFX) and gatifloxacin (GFX), have demonstrably increased anti-tuberculosis activity in vitro compared to ciprofloxacin (CPX) and ofloxacin (OFX). Improved pharmacokinetics and pharmacodynamics have also been observed with the later-generation fluoroquinolones (Table 1).^26^

**Table 1. Comparative PK/PD of fluoroquinolones after single oral dose in humans**

| Drug | Pharmacokinetics | | | Pharmacodynamics | | |
| --- | --- | --- | --- | --- | --- | --- |
|  | Dose  (mg) | C_max_  (µg/ml) | AUC  (µg-h/ml) | MIC_90_  (µg/ml) | C_Max_/MIC_90_ | AUC/MIC_90_ |
| Ciprofloxacin | 500 | 2.4 | 11.6 | 1.0 | 2.4 | 11.6 |
| Ofloxacin | 400 | 3 | 24 | 2.0 | 1.5 | 12 |
| Levofloxacin | 500 | 6.2 | 45 | 1.0 | 6.2 | 45 |
| Moxifloxacin | 400 | 4.3 | 39 | 0.5 | 8.6 | 78 |

Murine models of TB show clearly increasing anti-TB activity with increasing doses of ofloxacin, sparfloxacin and levofloxacin to doses greater than those usually employed.^27-28^ In humans, early studies of ciprofloxacin 500 mg daily and ofloxacin 400 mg daily for treatment of drug-susceptible TB indicated that these agents were well tolerated but did not improve upon standard treatment.^15, 29^ Subsequent reports demonstrated that increasing the dose led to more robust responses.^21^ When levofloxacin (which is twice as potent as ofloxacin on a per-gram basis) became available, it was used at a dose of 750 mg daily^30^ and resulted in improved outcomes.^31^ In patients with MDR-TB, it has been been observed that patients with isolates with in vitro ofloxacin resistance may still respond to regimens containing FQs that achieve increased serum concentrations.^32^A recent meta-analysis of 13 observational studies of XDR-TB treatment found that studies in which a higher proportion of patients received a later generation FQ (levofloxacin, moxifloxacin or non-marketed sparfloxacin) reported a higher proportion of favorable outcomes, suggesting that the pharmacodynamic advantage of more potent FQs can overcome resistance to ofloxacin.^2^ This phenomenon may be explained by the results of MIC testing of TB isolates showing that many isolates resistant to ofloxacin have MICs just above the clinical cutoff, indicating that increased FQ dosing might render them susceptible.^33-34^

Fluoroquinolones are the most active class of antimycobacterial agents currently available for use in patients with MDR-TB. Although there have been no controlled clinical trials of quinolone use in treatment of MDR-TB, a number of observational studies have linked fluoroquinolone use with good treatment outcomes.^15, 17-21^ Prior to the advent of the quinolones, MDR-TB treatment was characterized by a cure rate of only 56%, with 37% five year mortality.^16^ Including a quinolone in the regimen has led to improved overall cure rates to 61%-75% and ~20% mortality. In the absence of prospective clinical trials to determine the optimal dose of FQ for treatment of TB, doses which had been optimized for treatment of serious bacterial infections were simply carried over to TB treatment. Rapid emergence of resistance to FQ in patients being treated for MDR-TB suggests that these doses are suboptimal, and may even promote acquisition of antimycobacterial resistance, particularly when combined with suboptimal companion drugs.^21, 35^

### 2.1.4. Other new drugs for treatment of MDR-TB

Substantial improvement in treatment outcomes for patients with MDR-TB will require both optimization of fluoroquinolone dosing and addition to the regimen of new antimycobacterial agents that are more efficacious than current second-line anti-TB agents. Two promising compounds, bedaquiline and delamanid, have recently completed Phase 2 clinical trials in patients with MDR-TB. Bedaquiline, when added to an optimized background regimen (OBR) in a study of 159 patients, resulted in more rapid sputum culture conversion in liquid media than OBR plus placebo (12 weeks versus 18 weeks) and a higher proportion of cures at 24 months.^36-37^ A phase 2 study of delamanid using a similar design was recently reported.^37a^. While new FQ agents are being developed for use and anti-TB agents (TBK-613, KRQ-10018), none is yet in stage 1 trials.^38-39^

### 2.1.5. Why levofloxacin will be the drug of choice for treatment of MDR-TB

Ciprofloxacin is clearly inferior to the later generation FQ, and most experts recommend against its use for treatment of TB.^40^Ofloxacin is the same active moiety as levofloxacin but also contains 50% of the inactive isomer, so that 800 mg of ofloxacin is equivalent to 400 mg of levofloxacin. Thus, levofloxacin is preferable to ofloxacin for treatment of TB. Gatifloxacin and sparfloxacin have been taken off the market because of toxicities (dysglycemia and hypersensitivity, respectively). Therefore either levofloxacin or moxifloxacin will be the optimal FQ for TB treatment.

Moxifloxacin is relatively well tolerated at 400 mg daily, but in vitro studies have suggested that this dose is sub-optimal.^31^  However, there are few data on tolerability of higher doses, because the 400 mg dose is associated with substantial QT interval prolongation.^41^ Despite the fact that higher doses of moxifloxacin might be more efficacious,no reports of tolerability of higher doses have been published, and the sponsor will not support clinical studies using higher doses. Levofloxacin has no QT interval prolongation at doses up to 20 mg/kg daily.^42^ Unfortunately, both TMC-207 and OPC-67683 have the potential to cause QT-prolongation. For this reason, the current clinical studies of TMC-207 and OPC-67683 both require that moxifloxacin not be used in combination with the investigational product. Therefore, it is likely that, when these drugs are licensed for use in treating MDR-TB, as anticipated, the label will state that moxifloxacin should not be used concurrently. Therefore, levofloxacin will be the FQ of choice for treatment of MDR-TB for at least the next decade

### 2.1.6. Currently used levofloxacin doses are suboptimal

Evidence that levofloxacin, and more generally other late-generation fluoroquinolones, dose and activity against *M. tuberculosis* could be increased without sacrificing tolerance is emerging.^43^ Accumulated safety and tolerability data permit recommended LFX doses as high as 1000 mg.^44-45^ A recent early bactericidal activity (EBA) study demonstrated that levofloxacin 1000 mg per day in adults has activity against *M. tuberculosis* equivalent to currently employed dosage of 400 mg QD of moxifloxacin.^46^ Furthermore, this dose achieves comparable AUC/MIC and C_max_/MIC values to those achieved by GFX and MFX, each at 400 mg/day. In animal studies, increasing doses to the equivalent of 20 mg/kg resulted in greater decreases in *M. tuberculosis* colony counts.

The current study proposes to study levofloxacin at 4 doses (11 mg/kg, 14 mg/kg, 17 mg/kg and 20 mg/kg; these are roughly equivalent to 750 mg, 1000 mg, 1250 mg and 1500 mg per day for a 70-kg person. These doses will be given in combination with an optimized background regimen (OBR), for efficacy and tolerability in treatment of patients with MDR-TB. Patients will be followed intensively for the first six months of treatment, with the primary endpoint being time to culture conversion; subjects who have not experienced culture conversion at 6 months will be classified as being at increased risk for a poor treatment outcome. This efficacy endpoint will be combined with intensive pharmacokinetic sampling so that the optimal target AUC/MIC ratio for levofloxacin can be established. Based on murine studies, the target AUC/MIC will be 100.^47^

Levofloxacin at a dose of 11 mg/kg is well tolerated by patients with TB.^15, 30, 48^ Levofloxacin at 14 mg/kg/day has recently been adopted by some TB programs, and also appears to be well-tolerated.^44-45^ Levofloxacin has been used for treatment of TB meningitis at doses of 20 mg/kg/day in 15 adults with TB meningitis for 60 days.^49^No side effects attributable to the levofloxacin were seen.^50^ This dose was also studied in children with recurrent or persistent otitis media; 786 children ages <1 to 5 years received levofloxacin 20 mg/kg day for an average of 10.2 days.^51^ There were no significant differences in adverse effects between levofloxacin and the comparator regimen, amoxicillin/clavulanate 90 mg/kg/day. In a larger study of 2523 children treated with levofloxacin 20 mg/kg/day, a small but significant increase in musculoskeletal disorders, largely arthralgias, was seen in patients receiving this dose of levofloxacin (2.1% vs. 0.9% of subjects).^52^ It is possible that children excrete levofloxacin more rapidly than adults, so that the levofloxacin AUC achieved in patients in the proposed study could be higher than those well-tolerated in children. However, we believe that the frequency of intolerance to the highest study dose (20 mg/kg) among patients with MDR-TB will be low.

### 2.1.7. Emergence of fluoroquinolone resistance

Rates of quinolone resistance seen in *M. tuberculosis* isolates are increasing.^53^ In some cases, drug resistance to FQ among TB patients is associated with use of FQ for treatment of community acquired pneumonia.^54-55^ More worrisome has been the recent report of emergence of resistance to FQ in patients receiving FQ for treatment of MDR-TB; a rate of 20% acquisition of FQ resistance per year has been reported.^35^ In another study, 6 % of patients developed both FQ resistance and resistance to a second-line injectable agent.^56^ Such FQ resistance is usually attributable to with mutations in gyrA and gyrB.^57-58^ Disease in patients whose *M. tuberculosis* develops FQ resistance while on treatment for MDR-TB are called “Pre-XDR-TB” and have a high risk of progressing to XDR-TB. Studies with other fluoroquinolones have demonstrated that higher AUC must be achieved to prevent emergence of resistance than to kill *M. tuberculosis*.^57, 59^Thus, there may be additional benefit to increasing the AUC achieved with levofloxacin over and above that needed to maximize killing of *M. tuberculosis.*

### 2.1.8. Pharmacokinetic and pharmacodynamic parameters of levofloxacin

The PK/PD concentration of parameter that corresponds to maximal bactericidal (or sterilizing activity) for fluoroquinolones in the treatment of TB has not been identified.

Evidence from other serious bacterial infections suggests that this would be AUC/MIC ratio.^60^ This parameter is the best predictor in the mouse model of tuberculosis for the activity of fluoroquinolones; this model suggests that better responses will be seen when the AUC/MIC ratio is > 100.^47^ The relationship between antimycobacterial activity and AUC/MIC in patients with MDR-TB will be evaluated in the current proposal.

### 2.1.9. Assessing Response to MDR-TB treatment

Interim indicators of treatment response have been infrequently reported for MDR-TB. Time to culture conversion, however, has been associated with final outcomes of MDR-TB treatment.^61^ Even for drug-susceptible TB, the only partially validated surrogate endpoint for cure is dichotomous evaluation of sputum culture conversion at a fixed point in time.^62^ This endpoint is commonly assessed as the proportion culture negative rate at 2 months of regimen. This endpoint however, has serious limitations including limited specificity and sensitivity. Moreover, it requires sample sizes of 100-250 per arm for Phase 2b trials. This makes Phase 2b one of the key bottlenecks in TB drug development. Recently, an elegant analysis demonstrated the increased precision provided by measuring culture conversion as a continuous endpoint and calculating the outcome as time to culture conversion with survival analysis. Validation of such a continuous marker of a binary endpoint could lead to reduced sample sizes in future MDR-TB trials.^63-64^ Consequently, the present study will also assess rate of change in time to positivity in liquid medium as a surrogate marker of culture conversion in the first 6 months.

Serial Sputum Colony Counts (SSCC) have also been carefully studied as a surrogate endpoint for clinical trials of tuberculosis treatment. Plots of these counts over time show a biphasic decline in SSCC that varies by study arm. This work has confirmed that the decline has two phases, an early “fast” phase and a prolonged “slow” phase. Non-linear mixed effects regression analysis has demonstrated that the rate of decline of the slow phase predicts sterilizing activity of antituberculosis regimens.^64-65^ Use of SSCC as an endpoint in clinical trials is hampered by the expense and intensity of labor required for accurate assessment of this endpoint. Since sputum colony counts are highly correlated with Time-To-Detection (TTD) of *M. tuberculosis* from sputum in liquid medium,^66-68^Weiner et al. analyzed the results of TTD as a predictor of treatment failure and found that risk factors commonly associated with treatment failure were more strongly associated with TTD than with culture conversion at 2 months.^63^Hessling, et al also found that baseline TTD predicted both 2-month culture conversion and relapse in drug-susceptible TB.^69^ The present study will explore the relationship between TTD and outcomes in MDR-TB.

### 2.1.10. Alternative Study Designs

Studies of increasing drug dose to evaluate safety of higher doses are often performed in a stepwise fashion, such that one dose is studied and evaluated in a single group of patients before making a decision to enroll patients in the next-highest dose (a “dose-escalation study”). We have decided not to follow such a strategy for the following reasons:

1. We believe that doses of 20 mg/kg/day have already been demonstrated to be well-tolerated in man (see section 2.1.6) so that patients are not at risk for excessive occurrence of adverse events up to this dose.
2. The extremely poor clinical response to MDR-TB treatment and the high associated mortality with regimens containing lower doses of levofloxacin, combined with animal and in vitro data strongly suggesting that increased doses of levofloxacin could be more efficacious justifies the small potential for increased occurrence of adverse effects with doses up to 20 mg/kg/day.
3. The need to follow study subjects in each dose group for 6 months would mean that a study of 25 subjects at each of four doses would require 14 months per group (6 months to enroll 25 subjects+ 6 months to follow the last enrolled subject+2 months laboratory follow-up+2 months to decide on the next dose and begin enrollment), lengthening the study to at least 64 months.
4. Sequential evaluation of each dose would preclude randomization to dose. Without randomization to dose, each dose group would need to be substantially larger to achieve equivalent precision in the estimates of efficacy, tolerability and safety. We estimate that such a design would require approximately 50 subjects per group rather than 25.This would increase the number of study subjects exposed to higher doses and greatly increase the cost and duration of the study.

Therefore, we conclude that the minimal expectation of increased adverse events with doses up to 20 mg/kg is outweighed by the potential benefit of fewer exposed patients, shorter time to study results, decreased cost and the need for better treatment options for these patients.

## Scientific Rationale

Optimization of levofloxacin dose is important for two reasons. First, it is the least toxic of current “third-generation” fluoroquinolones; moxifloxacin, which is still under patent protection, is associated with substantial QT prolongation, while gatifloxacin induces disglycemia, may lead to diabetes, and has been removed from the market. New fluoroquinolones are still in preclinical development, and will not come to market for at least 8-10 years. Thus, levofloxacin is likely to be the most widely used fluoroquinolone for treatment of TB, especially MDR-TB, for the foreseeable future.

Second, optimization of levofloxacin dose minimizes the likelihood that FQ resistance will emerge during MDR-TB treatment. Hollow fiber studies of both ciprofloxacin and moxifloxacin have demonstrated that the target AUC for prevention of the emergence of resistance (Mutant Prevention Concentration, or MPC) in *M. tuberculosis* is substantially higher than that for bactericidal effect, and that unless this concentration is exceeded, resistant mutants are selected and replace the susceptible population.^57, 70^When used against gram-negative bacteria in an animal model, suboptimal concentrations of FQ not only lead to the emergence of FQ-resistant bacteria, but they also promote emergence of resistance to other antibiotics, through SOD-mediated mutagenesis.^71^ Thus, dosing strategies that result in high AUC/MIC ratios could be important in prevention of the emergence of resistance to FQ, and to other agents in *M. tuberculosis*.

By identifying target AUC and AUC/MIC ratios, and by increasing our understanding of their relationship to levofloxacin dose, this study will allow physicians to treat MDR-TB patients with the highest doses of levofloxacin that are associated with acceptable tolerability. However, Most patients with MDR-TB receive treatment in geographic areas where monitoring of serum drug concentrations and subsequent adjustment of doses is impractical. Therefore we propose development of an algorithm that will predict the mg/Kg dose most likely to achieve the AUC and AUC/MIC ratios. This is complicated by the fact that the levofloxacin AUC distribution in patients with TB can be very broad; more than 100-fold differences in AUC have been seen with the same dose in two separate studies ^72-73^ Therefore, optimizing levofloxacin dosing for TB treatment will both require a better understanding of the factors underlying this variability and a strategy for optimizing dosing despite this variability.

We hypothesize that the proposed examination of clinical predictors of AUC will provide result in easily implemented identification of patients whose AUC can be expected to be at the high and low ends of the distribution, allowing construction of an algorithm that will generate treatment guidelines to prevent under- or over-dosing such patients. Thus, physicians without access to levofloxacin concentrations will be able to make a reasonable estimate of the highest dose that the patient is likely to tolerate, thus achieving the greatest efficacy.

This study will also provide firm evidence for the efficacy of the highest tolerated dose of levofloxacin, up to 20mg/Kg. Currently, moxifloxacin is being used by some programs as an alternative to levofloxacin for treatment of MDR-TB. There is much enthusiasm for moxifloxacin, despite the lack of evidence that it is superior to levofloxacin; in fact, a recent EBA study demonstrated that levofloxacin 14 mg/Kg per day has activity against *M. tuberculosis* that is similar to that of moxifloxacin at the standard dose (400 mg per day).^46^ Moreover, levofloxacin will soon be off patent worldwide, while moxifloxacin will remain on patent for 5 more years. Therefore it is important to define the most effective dose of levofloxacin for TB treatment that is both safe and well-tolerated.

## Potential Risks and Benefits

### Potential Risks

#### 2.3.1.1 WARNINGS AND PRECAUTIONS

##### 2.3.1.1.1 Tendinopathy and Tendon Rupture

Fluoroquinolones, including levofloxacin, are associated with an increased risk of tendinitis and tendon rupture in all ages. This adverse reaction most frequently involves the Achilles tendon, and rupture of the Achilles tendon may require surgical repair. Tendinitis and tendon rupture in the rotator cuff (the shoulder), the hand, the biceps, the thumb, and other tendon sites have also been reported. The risk of developing fluoroquinolone-associated tendinitis and tendon rupture is further increased in older patients usually over 60 years of age, in those taking corticosteroid drugs, and in patients with kidney, heart or lung transplants. Factors, in addition to age and corticosteroid use, that may independently increase the risk of tendon rupture include strenuous physical activity, renal failure, and previous tendon disorders such as rheumatoid arthritis. Tendinitis and tendon rupture have been reported in patients taking fluoroquinolones who do not have the above risk factors. Tendon rupture can occur during or after completion of therapy; cases occurring up to several months after completion of therapy have been reported. levofloxacinshould be discontinued if the patient experiences pain, swelling, inflammation or rupture of a tendon. Patients should be advised to rest at the first sign of tendinitis or tendon rupture, and to contact their healthcare provider regarding changing to a non-quinolone antimicrobial drug.

##### 2.3.1.1.2 Hypersensitivity Reactions

Serious and occasionally fatal hypersensitivity and/or anaphylactic reactions have been reported in patients receiving therapy with fluoroquinolones, including levofloxacin. These reactions often occur following the first dose. Some reactions have been accompanied by cardiovascular collapse, hypotension/shock, seizure, loss of consciousness, tingling, angioedema (including tongue, laryngeal, throat, or facial edema/swelling), airway obstruction (including bronchospasm, shortness of breath, and acute respiratory distress), dyspnea, urticaria, itching, and other serious skin reactions. Levofloxacin should be discontinued immediately at the first appearance of a skin rash or any other sign of hypersensitivity. Serious acute hypersensitivity reactions may require treatment with epinephrine and other resuscitative measures, including oxygen, intravenous fluids, antihistamines, corticosteroids, pressor amines, and airway management, as clinically indicated*.*

##### 2.3.1.1.3 Other Serious and Sometimes Fatal Reactions

Other serious and sometimes fatal events, some due to hypersensitivity, and some due to uncertain etiology, have been reported rarely in patients receiving therapy with fluoroquinolones, including levofloxacin. These events may be severe and generally occur following the administration of multiple doses. Clinical manifestations may include one or more of the following:

- fever, rash, or severe dermatologic reactions (e.g., toxic epidermal necrolysis, Stevens-Johnson Syndrome);
- vasculitis; arthralgia; myalgia; serum sickness;
- allergic pneumonitis;
- interstitial nephritis; acute renal insufficiency or failure;
- hepatitis; jaundice; acute hepatic necrosis or failure;
- anemia, including hemolytic and aplastic; thrombocytopenia, including thrombotic thrombocytopenic purpura; leukopenia; agranulocytosis; pancytopenia; and/or other hematologic abnormalities.

The drug should be discontinued immediately at the first appearance of skin rash, jaundice, or any other sign of hypersensitivity and supportive measures instituted*.*

##### 2.3.1.1.4 Hepatotoxicity

Post-marketing reports of severe hepatotoxicity (including acute hepatitis and fatal events) have been received for patients treated with levofloxacin. No evidence of serious drug-associated hepatotoxicity was detected in clinical trials of over 7,000 patients. Severe hepatotoxicity generally occurred within 14 days of initiation of therapy and most cases occurred within 6 days. Most cases of severe hepatotoxicity were not associated with hypersensitivity. The majority of fatal hepatotoxicity reports occurred in patients 65 years of age or older and most were not associated with hypersensitivity. Levofloxacin should be discontinued immediately if the patient develops signs and symptoms of hepatitis*.*

##### 2.3.1.1.5 Central Nervous System Effects

Convulsions and toxic psychoses have been reported in patients receiving fluoroquinolones, including levofloxacin. Fluoroquinolones may also cause increased intracranial pressure and central nervous system stimulation which may lead to tremors, restlessness, anxiety, lightheadedness, confusion, hallucinations, paranoia, depression, nightmares, insomnia, and, rarely, suicidal thoughts or acts. These reactions may occur following the first dose. If these reactions occur in patients receiving levofloxacin, the drug should be discontinued and appropriate measures instituted. As with other fluoroquinolones, levofloxacin should be used with caution in patients with a known or suspected central nervous system (CNS) disorder that may predispose them to seizures or lower the seizure threshold (e.g., severe cerebral arteriosclerosis, epilepsy) or in the presence of other risk factors that may predispose them to seizures or lower the seizure threshold (e.g., certain drug therapy, renal dysfunction.).

##### 2.3.1.1.6 Clostridium difficile-Associated Diarrhea

*Clostridium difficile*-associated diarrhea (CDAD) has been reported with use of nearly all antibacterial agents, including levofloxacin, and may range in severity from mild diarrhea to fatal colitis. Treatment with antibacterial agents alters the normal flora of the colon leading to overgrowth of *C. difficile*.

*C. difficile* produces toxins A and B which contribute to the development of CDAD. Hypertoxin producing strains of *C. difficile* because increased morbidity and mortality, as these infections can be refractory to antimicrobial therapy and may require colectomy. CDAD must be considered in all patients who present with diarrhea following antibiotic use. Careful medical history is necessary since CDAD has been reported to occur over two months after the administration of antibacterial agents.

If CDAD is suspected or confirmed, ongoing antibiotic use not directed against *C. difficile* may need to be discontinued. Appropriate fluid and electrolyte management, protein supplementation, antibiotic treatment of *C. difficile,* and surgical evaluation should be instituted as clinically indicated.

##### 2.3.1.1.7 Peripheral Neuropathy

Rare cases of sensory or sensorimotor axonal polyneuropathy affecting small and/or large axons resulting in paresthesias, hypoesthesias, dysesthesias and weakness have been reported in patients receiving fluoroquinolones, including levofloxacin. Levofloxacin should be discontinued if the patient experiences symptoms of neuropathy including pain, burning, tingling, numbness, and/or weakness or other alterations of sensation including light touch, pain, temperature, position sense, and vibratory sensation in order to prevent the development of an irreversible condition*.*

##### 2.3.1.1.8 Prolongation of the QT Interval

Some fluoroquinolones, including levofloxacin, have been associated with prolongation of the QT interval on the electrocardiogram and infrequent cases of arrhythmia. Rare cases of torsade de pointes have been spontaneously reported during post-marketing surveillance in patients receiving fluoroquinolones, including levofloxacin. Levofloxacinshould be avoided in patients with known prolongation of the QT interval, patients with uncorrected hypokalemia, and patients receiving Class IA (quinidine, procainamide), or Class III (amiodarone, sotalol) antiarrhythmic agents. Elderly patients may be more susceptible to drug-associated effects on the QT interval*.*

##### 2.3.1.1.9 Musculoskeletal Disorders in Pediatric Patients and Arthropathic Effects in Animals

Levofloxacin is indicated in pediatric patients (≥6 months of age) only for the prevention of inhalational anthrax (post-exposure). An increased incidence of musculoskeletal disorders (arthralgia, arthritis, tendonopathy, and gait abnormality) compared to controls has been observed in pediatric patients receiving levofloxacin.

In immature rats and dogs, the oral and intravenous administration of levofloxacin resulted in increased osteochondrosis. Histopathological examination of the weight-bearing joints of immature dogs dosed with levofloxacin revealed persistent lesions of the cartilage. Other quinolones also produce similar erosions in the weight-bearing joints and other signs of arthropathy in immature animals of various species*.*

##### 2.3.1.1.10 Blood Glucose Disturbances

As with other fluoroquinolones, disturbances of blood glucose, including symptomatic hyper- and hypoglycemia, have been reported with levofloxacin, usually in diabetic patients receiving concomitant treatment with an oral hypoglycemic agent (e.g., glyburide) or with insulin. In these patients, careful monitoring of blood glucose is recommended. If a hypoglycemic reaction occurs in a patient being treated with levofloxacin, levofloxacin should be discontinued and appropriate therapy should be initiated immediately*.*

##### 2.3.1.1.11 Photosensitivity/Phototoxicity

Moderate to severe photosensitivity/phototoxicity reactions, the latter of which may manifest as exaggerated sunburn reactions (e.g., burning, erythema, exudation, vesicles, blistering, edema) involving areas exposed to light (typically the face, “V” area of the neck, extensor surfaces of the forearms, dorsa of the hands), can be associated with the use of fluoroquinolones after sun or UV light exposure. Therefore, excessive exposure to these sources of light should be avoided. Drug therapy should be discontinued if photosensitivity/phototoxicity occurs*.*

##### 2.3.1.1.12 Development of Drug Resistant Bacteria

Prescribing levofloxacinin the absence of a proven or strongly suspected bacterial infection or a prophylactic indication is unlikely to provide benefit to the patient and increases the risk of the development of drug-resistant bacteria*.*

#### 2.3.1.2 ADVERSE REACTIONS

##### 2.3.1.2.1 Serious and Otherwise Important Adverse Reactions

The following serious and otherwise important adverse drug reactions are discussed in greater detail in other sections of labeling:

- Tendon Effects
- Hypersensitivity Reactions
- Hepatotoxicity
- Central Nervous System Effects
- *Clostridium difficile*-Associated Diarrhea
- Peripheral Neuropathy
- Prolongation of the QT Interval
- Musculoskeletal Disorders in Pediatric Patients
- Blood Glucose Disturbances
- Photosensitivity/Phototoxicity
- Development of Drug Resistant Bacteria

Hypotension has been associated with rapid or bolus intravenous infusion of levofloxacin. Levofloxacin should be infused slowly over 60 to 90 minutes, depending on dosage*.*

Crystalluria and cylindruria have been reported with quinolones, including levofloxacin. Therefore, adequate hydration of patients receiving levofloxacin should be maintained to prevent the formation of highly concentrated urine*.*

##### 2.3.1.2.2 Clinical Trial Experience

Because clinical trials are conducted under widely varying conditions, adverse reaction rates observed in the clinical trials of a drug cannot be directly compared to rates in the clinical trials of another drug and may not reflect the rates observed in practice.

The data described below reflect exposure to levofloxacin in 7537 patients in 29 pooled Phase 3 clinical trials. The population studied had a mean age of 50 years (approximately 74% of the population was < 65 years of age), 50% were male, 71% were Caucasian, and 19% were Black. Patients were treated with levofloxacin for a wide variety of infectious diseases. Patients received levofloxacin doses of 750 mg once daily, 250 mg once daily, or 500 mg once or twice daily. Treatment duration was usually 3-14 days, and the mean number of days on therapy was 10 days.

The overall incidence, type and distribution of adverse reactions was similar in patients receiving levofloxacin doses of 750 mg once daily, 250 mg once daily, and 500 mg once or twice daily. Discontinuation of levofloxacin due to adverse drug reactions occurred in 4.3% of patients overall, 3.8% of patients treated with the 250 mg and 500 mg doses and 5.4% of patients treated with the 750 mg dose. The most common adverse drug reactions leading to discontinuation with the 250 and 500 mg doses were gastrointestinal (1.4%), primarily nausea (0.6%); vomiting (0.4%); dizziness (0.3%); and headache (0.2%). The most common adverse drug reactions leading to discontinuation with the 750 mg dose were gastrointestinal (1.2%), primarily nausea (0.6%), vomiting (0.5%); dizziness (0.3%); and headache (0.3%).

Adverse reactions occurring in ≥1% of levofloxacin-treated patients are shown in Table 2. The most common adverse drug reactions (≥3%) are nausea, headache, diarrhea, insomnia, constipation, and dizziness.

**Table 2: Common (≥1%) Adverse Reactions Reported in Clinical Trials with levofloxacin**

| **System/Organ Class** | **Adverse Reaction** | **% (N=7537)** |
| --- | --- | --- |
| **Infections and Infestations** | moniliasis | 1 |
| **Psychiatric Disorders** | Insomnia | 4 |
| **Nervous System Disorders** | Headache, dizziness | 6, 3 |
| **Respiratory, Thoracic and Mediastinal Disorders** | Dyspnea | 1 |
| **Gastrointestinal Disorders** | Nausea, diarrhea, constipation, abdominal pain, vomiting, dyspepsia | 7, 5, 3, 2, 2, 2 |
| **Skin and Subcutaneous Tissue Disorders** | Rash*[see Warnings and Precautions (5.2)]* Pruritus | 2 1 |
| **Reproductive System and Breast Disorders** | Vaginitis | 1b |
| **General Disorders and Administration Site Conditions** | Edema, injection site reaction, chest pain | 1 11 |

#### 2.3.1.3 Alternative data gathering procedures

In studies to determine the optimal dose of a new drug, dose-escalation is usually employed. In such a design, one assures that one dose is safe and well tolerated before increasing the dose in search of greater efficacy. However, levofloxacin is not a new drug and its safety and toxicity profile is well known. There is ample evidence that it is well tolerated by patients with TB for prolonged periods of time at doses of 11mg/kg and 14 mg/kg. In addition, there are a number of studies indicating that it is well tolerated at doses up to of 20 mg/kg, the highest dose proposed for this study (see section 2.1.6). The alternative to the proposed study design would be to first examine the safety and tolerability of 17 mg/kg and then proceed to adding the 20 mg/kg group. This would require a substantial alteration in the present study design, as the analysis relies on randomization to the four dose arms, and sequential addition of dose arms would preclude such randomization.

We believe that the likelihood of toxicity with the 17 mg/kg dose is so low that study of this dose alone is not necessary. Therefore, our design is, in effect, a dose escalation design that begins with the escalation from 14mg/kg to 20 mg/kg. Moreover, we have built in an unbIinded evaluation of the safety of the 20 mg/kg dose by the DSMB As noted in Section 9.6, “the DSMB will review safety and tolerability of the all doses three months after 40 patients have been enrolled and again three months after 60 patients have been enrolled. If more than 50% of patients in any dose arm have permanently discontinued treatment for any reason before completing 24 weeks of treatment, the DSMB will recommend that enrollment in that arm be terminated.

### Known Potential Benefits

Levofloxacin is known to have benefit for treatment of TB in persons with disease caused by susceptible isolates. Therefore, participation in this study will ensure that patients are being treated with an agent that is effective against the causal organism of their disease. In addition, patients in this study will have additional anti-tuberculosis agents selected to have activity against their organism, resulting in use of an optimized regimen. Thus patients may have more rapid treatment response, accelerated conversion, enabling them to return to work more quickly and pose less risk to their households and communities. In addition, study participation is likely to gain them quicker access to drug susceptibility testing and subsequent modification of treatment regimens and more careful follow-up than would be received by patients receiving standard TB care.

# Objectives

## Study Objectives

**Primary Objectives**

1. Determine the levofloxacin AUC/MIC that provides the shortest time to sputum culture conversion on solid medium.
2. Determine the highest levofloxacin AUC that is both safe and associated with fewer than 25% of patients discontinuing or reducing their dose of levofloxacin.
3. Develop a dosing algorithm to achieve the AUC associated with maximal efficacy and acceptable safety and tolerability.

**Secondary Objectives**

1. Determine the levofloxacin AUC/MIC that provides the shortest time to sputum culture conversion in liquid medium.
2. Determine if baseline PZA susceptibility is associated with shorter time to sputum culture conversion after controlling for levofloxacin AUC
3. Describe the relationship between assigned study dose (in mg/kg/day and time to sputum culture conversion, safety and ability to complete 24 months of assigned study dose.

## Study Outcome Measures

### Primary Outcome Measures

**Endpoint for Primary Objective 1. Efficacy** – The primary efficacy endpoint is the time to sputum culture conversion from positive to negative for *M. tuberculosis* growth on solid medium. This is defined as the time from initiation of study treatment to the first of two successive negative cultures one study visit apart that are not followed by a culture-positive specimen within 28 weeks of treatment initiation. To ensure that each subject will be evaluable for the primary endpoint, bi-weekly sputum cultures will be collected for 12 weeks, then every 4 weeks through 24 weeks of treatment. If the week 20 culture is positive (and the week 24 culture is pending or negative) the patient should provide a week 28 specimen for culture to allow the assessment of conversion at week 24. Subjects who are unable to produce specimens for sputum culture despite repeated attempts will be considered to have had a negative culture on that date. Sputum cultures that are overgrown by bacteria and/or yeast despite additional laboratory measures to recover mycobacteria (e.g., re-decontamination of the specimen or pellet) will be deemed un-evaluable.

**Endpoint for Primary Objective 2a. Safety** – The primary safety endpoint will be the number of grade 3 or greater adverse events (AEs) occurring up to and including the time on study drug plus four weeks post study drug completion.

**Endpoint for Primary Objective 2b. Tolerability** – The primary endpoint for the analysis of tolerability will be the ability to complete 24 weeks of treatment with the assigned levofloxacin dose (in mg/kg at enrollment). Subjects who continue the assigned study dose but change to twice-daily administration of the same total daily dose prior to completion of 24 weeks of therapy will be considered to be on the assigned dose and will not have met a study endpoint; subjects who change their assigned dose to levofloxacin at a reduced daily dose prior to protocol-defined completion of 24 weeks of therapy will be considered to have met the study endpoint of failing to complete the assigned levofloxacin dose. Completion of 24 weeks of levofloxacin treatment is defined as the receipt of 168 daily doses of assigned study drug dose within 200 days of initiation of study regimen.

**Endpoint for Primary Objective 3. Dosing algorithm –** The primary outcome for this objective is the development of a dosing algorithm that identifies the levofloxacin dose (in mg/Kg) for a given patient that can be expected to achieve the AUC associated with maximal efficacy and acceptable safety and tolerability.

### Secondary Outcome Measures

**Endpoint for Secondary Objective 1**. **Efficacy of levofloxacin** – The endpoint is the time to sputum culture conversion from positive to negative for *M. tuberculosis* growth on **liquid medium**.

**Endpoint for Secondary Objective 2. Efficacy of PZA**. – The endpoint for this analysis is time to sputum culture conversion in solid and liquid medium (2 analyses), after controlling for levofloxacin AUC/MIC.

**Endpoint for Secondary Objective 3. Relationship between dose and efficacy and toxicity.** - The endpoint is the time to sputum culture conversion from positive to negative for *M. tuberculosis* growth (in both solid and liquid medium). The endpoint for the analysis of tolerability will be the ability to complete 24 weeks of treatment with the assigned levofloxacin dose. The primary safety endpoint will be the number of grade 3, 4 and 5 adverse events (AEs), occurring up to and including the time on study drug plus four weeks post study drug completion.

# Study Design

In this Phase 2 study, we propose to determine the levofloxacin dose that achieves the greatest reduction in mycobacterial burden with acceptable tolerability by randomizing 100 patients with MDR-TB to the mg/kg equivalent of 750, 1000, 1250 and 1500 mg daily (in combination with Optimized Background Regimen, OBR) and observing the effect of the drug concentrations achieved on time to sputum culture conversion over the 6-month period. A second outcome of interest will be the ability to complete 6 months of treatment with the assigned levofloxacin dose. In addition, because there is substantial variability in levofloxacin drug concentration achieved within a given dose category, we will construct and algorithm to allow clinicians to select the levofloxacin dose most likely result in achieving the optimal levofloxacin AUC while minimizing toxicity.

## Study Population

The study will enroll 100 adults with pulmonary MDR-TB, sputum that is isoniazid and rifampin resistant by MTBDRplus and fluoroquinolone susceptible by MTBDRsl, HIV positive or negative (but not unknown), and with a weight at or greater than 40kg, at sites in Peru and South Africa. Enrollment is expected to occur over a 24-month period, with 6 months of follow-up for each study subject. This will be a blinded, controlled trial, but there is no placebo arm. See sections 5.1 and 5.2 for complete inclusion and exclusion criteria.

Patients will be randomized to one of four levofloxacin dose categories (see section 6.2 for details). The study will be blinded, but not placebo-controlled; all subjects will receive the same number of pills, with varying proportions of active drug and placebo. All patients will receive at least 11 mg/kg daily of levofloxacin, the current WHO recommended dose.

## Treatment delivery and duration

After randomization, subjects will have daily administration of study drug and companion drugs initiated by DOT. Study drug and companion drugs (OBR) will be given by DOT 7 days a week. However, DOT may be supervised by non-study staff (but not family members). The duration of the study regimen, while defined as “24 weeks”, will be determined by the number of doses ingested, not by calendar time. Patients will have completed Study regimen when they have ingested 168 directly-observed, daily doses. To be eligible for inclusion in the per-protocol analysis of culture conversion, study regimen must have been completed within no less than 168 calendar days and no more than 200 calendar days.

## Assessment of study objectives and measurement of endpoints

- Each subject will have intensive PK analysis performed over a 24-hour period between day 14 and day 28 of treatment.
- All baseline *M. tuberculosis* isolates will have MIC for levofloxacin and PZA susceptibility testing performed at the CDC Mycobacteriology laboratory.
- All subjects will have follow-up sputum cultures performed at pre-specified intervals (see appendix A) at the local mycobacteriology laboratory on 7H11S solid medium and in MGIT 960 liquid medium. Subjects who are unable to produce sputum after induction will be classified as “Sputum culture-negative”.

## Optimized Background Regimen (OBR)

**For this study “OBR” will mean optimized regimen, not including a quinolone. OBR will be selected at the discretion of the study investigator to conform with standards of care and local site guidelines (Table** 3**). In general, the OBR regimen should include at least 3 drugs (other than levofloxacin) to which the patient’s isolate is not expected to be resistant, with one of these being an injectable agent, at the usual recommended doses.^74^ If sensitivity cannot be confirmed, and/or there has been prior exposure to drugs included in the regimen, more than 3 drugs should be added (in addition to levofloxacin) to ensure that the regimen contains at least 4 likely effective drugs.^74^**

**Table 3. Customary MDR-TB OBR regimen, by site (in addition to fluoroquinolone)**

| ****Site**** | ****Injectable**** | ****Drug 2**** | ****Drug 3**** | ****Drug 4**** | ****Drug 5**** |
| --- | --- | --- | --- | --- | --- |
| ****Lima**** | **Kanamycin** | **PZA** | **Ethambutol** | **Ethionamide** | **Cycloserine** |
| ****Cape Town**** | **Kanamycin** | **PZA** | **Ethionamide** | **Terizidone** |  |

**OBR may be changed by the provider at any time during the study for one of the following reasons:**

**1) in vitro susceptibility test results become available which indicate resistance to one or more of the drugs in the OBR regimen;**

**2) the patient develops toxicity to one or more of the drugs in the OBR regimen necessitating discontinuation of the drug.**

Change of OBR will not be considered a study endpoint. However, patients who change levofloxacin dose because of inability to tolerate the assigned daily levofloxacin/placebo dose will be considered to have met a study endpoint.

# Study Enrollment and Withdrawal

Study entry is open to males and females, age 18 years or older, of any ethnic background, who meets study eligibility criteria.  Historically, approximately 60% of patients with tuberculosis at clinical sites are males. The recent multi-center TMC-207 trial reported their subjects were predominantly male (74%) and HIV-negative (87%), with a median age of 33 years (range, 18 to 57). We would expect similar distributions in the study population of MDR-TB patients, either newly diagnosed or previously treated. The gender, ethnicity, and socioeconomic background of study subjects are expected to mirror that of the populations served by the study sites, and that of the population most affected by multidrug-resistant tuberculosis. This study will include HIV infected MDR-TB patients, with no restriction on CD4 count concentration, and uninfected individuals with MDR-TB.

It is anticipated that enrollment will occur at the study sites as follows: Cape Town (projected enrollment: 50 subjects), North Lima (20 subjects), East Lima (30 subjects).

## Randomization Inclusion Criteria

1. Patients with pulmonary TB
2. Sputum that is isoniazid and rifampin resistant by MTBDRplus and fluoroquinolone susceptible by MTBDRsl
3. HIV seropositive or seronegative but not unknown HIV serostatus. If the last documented negative HIV test was more than 3 months prior to enrollment the current serostatus must be assessed.
4. Age ≥ 18 years.
5. Weight > 40 Kg
6. Karnofsky score of > 60 (see Appendix B)
7. Willingness by the patient to attend scheduled follow-up visits and undergo study assessments.
8. Women with child-bearing potential must agree to practice an adequate birth control^[[1]](#footnote-2)^ or to abstain from heterosexual intercourse during study regimen.
9. Laboratory parameters (performed within 14 days prior to enrollment):

- Estimated Serum creatinine clearance should be >50, using nomogram^78^
- Hemoglobin concentration ≥ 9.0 g/dL
- Platelet count of ≥ 80,000/mm3
- Absolute neutrophil count (ANC) > 1000/ mm3
- Negative pregnancy test (for women of childbearing potential) during randomization/baseline
- CD4 count if HIV infected (within 6 months)
- Serum ALT and total bilirubin <3 times upper limit of normal

1. Able to provide informed consent

## Randomization Exclusion Criteria

1. Currently breast-feeding or pregnant.
2. Known allergy or intolerance to or toxicity from fluoroquinolones or other medications utilized in this study.
3. In the judgment of the physician the patient is not expected to survive for 6 months
4. Anticipated surgical intervention for the treatment of pulmonary tuberculosis
5. Participation in another investigational drug trial within the past 30 days
6. Concurrent use of known QT-prolonging drugs: a list of such medications can be found at http://www.azcert.org/medical-pros/drug-lists/printable-drug-list.cfm
7. Poorly controlled diabetes, defined as HgB A1c >9%
8. Known glucose-6-phosphate dehydrogenase (G6PD) deficiency
9. Use of quinolone for 7 days within past 30 days
10. QT_c_ interval (Fridericia corrected) greater than 450 msec for men and women

## Treatment Assignment Procedures

### Randomization Procedures

Eligible patients giving consent to be enrolled in the trial will be randomized to one of the four treatment arms in the ratio 1:1:1:1. Randomization will be done using pre-prepared lists using blocks of varying sizes with separate lists prepared for each site. Since the sample size is small, randomization will only be stratified by site and, in South Africa, by HIV status. The randomization list will be prepared by a statistician independent to the study.

### Masking Procedures

Generalized unblinding of treatment allocation for all patients will not occur until all enrolled have finished study therapy (minimum 168 doses) plus four weeks of post-last dose of study therapy follow-up.  There is a potential situation that may lead to earlier unblinding of treatment on a patient by patient basis, when, in the opinion of the study investigator, and the concurrence with the protocol team, the definitive attribution of the adverse event to the study drug will benefit the management of the patient’s disease.  Investigators must petition the study protocol team, which will review and endorse or decline to endorse the request.  All unscheduled early unblinding episodes will be reported to the DSMB at the same time as the reporting of the related adverse event.

### Reasons for Withdrawal of Study Drug

Discontinuation of study drug is strongly advised for patients who experience a grade 3 adverse event or higher that is Definitely or Probably related to study drug (see section 9.2.1 for definitions). Given the severity and relatively poor prognosis of the treatment of MDR TB in the absence of a fluoroquinolone, patients, with the advice of their clinicians, may wish to continue treatment with open label levofloxacin 750 mg daily or other fluoroquinolone while being closely monitored.

Discontinuation of Study Drug is also strongly advised if the patient develops:

- ventricular arrhythmia
- prolongation of the heart rate-corrected QT interval (*QTc by Fridericia method)* where the QTc is above 500 ms (for either males or females)
- severe neuropsychiatric effect (e.g., psychosis, convulsions, syncope) that is not believed to be caused by other TB drugs (e.g. cycloserine, terizidone)
- *Clostridium difficille* colitis
- significant joint pain or tendon rupture
- pregnancy once treatment started
- symptomatic hypoglycemia
- patient’s initial isolate is determined by phenotypic resistance testing to be susceptible to isoniazid or rifampin or resistant to ofloxacin

Given the severity and relatively poor prognosis of the treatment of MDR TB in the absence of a fluoroquinolone, patients, with the advice of their clinicians and depending on the circumstance, may wish to continue treatment with levofloxacin 750 mg/day or other fluoroquinolone while being closely monitored.

### Handling of Patients whose Study Drug is Withdrawn

Patients who permanently discontinue study treatment prior to completion of the study, if possible, will be followed for 6 months of MDR treatment or 4 weeks past discontinuation of study drug depending on which option proves to be longer. Patients may be taken off study drug but continue to receive follow-up in the following circumstances:

- Drug-related toxicity (see section 6.3 Modification of Study Intervention/Investigational Product for a Participant )
- Requirement for prohibited concomitant medications
- Any reason deemed necessary by the investigator or treating physician
- If the patients requests to stop study treatment, but does not withdraw consent for follow-up

Patients who withdraw consent for further participation, including follow-up, prior to completion of the study will not undergo any further study procedures or data collection. Data collected prior to withdrawal will be analyzed with complete data sets as is appropriate (e.g., time to sputum negativity for a patient who withdraws after becoming AFB negative).

### Termination of Study

The study may be terminated by DMID, the PI, one of the IRBs, the US FDA or another regulatory agency. Termination could occur after the development of unacceptable toxicities or as a recommended outcome of a DSMB review.

# Study Intervention/Investigational Product

## Study Product Description

LEVOFLOXACIN is a quinolone antibiotic used to treat lung, sinus, skin, and urinary tract infections caused by bacteria. Chemically, levofloxacin, a chiral fluorinated carboxyquinolone, is the pure (-)-(S)-enantiomer of the racemic drug substance ofloxacin. The chemical name is (-)-(S)-9fluoro-2,3-dihydro-3-methyl-10-(4-methyl-1-piperazinyl)-7-oxo-7H-pyrido[1,2,3-de]-1,4benzoxazine-6-carboxylic acid hemihydrate.


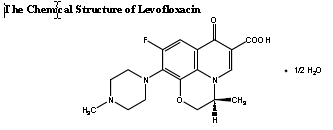


***Pharmacokinetic and pharmacodynamic parameters of levofloxacin***

The PK/PD parameter that corresponds to maximal bactericidal (or sterilizing activity) for fluoroquinolones in the treatment of TB has not been identified. Evidence from other serious bacterial infections has suggested that the AUC/MIC ratio correlates with bactericidal efficacy.^60^ This parameter is the best predictor in the mouse model of tuberculosis for the activity of fluoroquinolones.^47^ The relationship between antimycobacterial activity and AUC/MIC in patients with MDR-TB will be evaluated in the current proposal. If such an association is confirmed, it is likely to be generalizable to all drugs of the FQ class.

***Tolerability of levofloxacin***

Levofloxacin up to 750mg per day is well tolerated.^15, 30, 48^ Levofloxacin use at 1000 mg per day has recently been adopted by some TB programs, but there are few data on the efficacy and tolerability of this dose. Levofloxacin has been used in small numbers of patients with TB at doses up to 20 mg/kg without notable serious adverse events.^50, 75^

***Levofloxacin Drug-Drug Interactions***

LEVOFLOXACIN Tablets should be administered at least three hours before or three hours after antacids containing magnesium, aluminum, as well as sucralfate, metal cations such as iron, and multivitamin preparations with zinc or didanosine chewable/buffered tablets or the pediatric powder for oral solution. The potential for pharmacokinetic drug interactions between LEVOFLOXACIN and antacids warfarin, theophylline, cyclosporine, digoxin, probenecid, and cimetidine has been evaluated and dose adjustment is not required for concomitant use.

***Quinolone absorption and excretion***

Levofloxacin is absorbed from the intestine and excreted from the kidney. Dose adjustment is required for patients with significant renal insufficiency. If patient creatinine clearance falls below 50 ml/min, dose adjustment will be performed by unblinded pharmacist.

### Acquisition

Levofloxacin and matching placebo – which consist of the excipient contained in the levofloxacin tablets with no active drug - will be obtained from MacLeods Pharmaceuticals, Ltd., Mumbai India.

Study sites will supply all other antituberculosis drugs. Such drugs will be purchased from distributors/manufacturers distributing products which are approved by a stringent regulatory authority or which are pre-qualified by WHO Program on Essential Drugs. Inventory of other antituberculosis drugs will be monitored by individual sites, but will not be monitored by the study.

The investigator, or his/her designee, will acknowledge receipt of and keep an inventory of levofloxacin/placebo. Study staff has the responsibility to assure that study drugs are dispensed to patients in compliance with the protocol.

### Formulation, Packaging, and Labeling

Levofloxacin will be formulated as 250 mg tablets and packaged and labeled in a way that is consistent with FDA guidelines (package insert for Macleods levofloxacin has been developed as part of Macleods approval of its levofloxacin product by FDA).

### Product Storage and Stability

Levofloxacin should be stored in tightly closed containers, in a dry place, away from excessive heat (>40 degrees centigrade).

## Dosage, Preparation and Administration of Study Intervention/Investigational Product

**Administration of levofloxacin/placebo and other anti-tuberculosis drugs in the patient’s treatment regimen will be directly observed seven days a week.** However, DOT may be supervised by trained, non-study personnel 2 of the 7 days per week. Each dose should be recorded in the DOT log.

Levofloxacin dosing will be weight based at the time of enrollment, with two dosing bands as described in Table 4. To retain blinding, all participants will receive 6 tablets. The exact number of tablets containing active agent and the exact number containing placebo are shown in Table 4.

**Table 4. Dosing of LFX by treatment arm and weight at enrollment**

| ****Weight Band**** | ****11 mg/kg**** | | ****14 mg/kg**** | | ****17 mg/kg**** | | ****20 mg/kg**** | |
| --- | --- | --- | --- | --- | --- | --- | --- | --- |
| **L=250 mg LFX**  P=250 mg placebo | **# LFX & placebo** | **Total LFX (mg)** | **# LFX & placebo** | **Total LFX(mg)** | **# LFX & placebo** | **Total**  **LFX (mg)** | **# LFX & placebo** | **Total LFX (mg)** |
| **<60 kg** | **3L*+3P** | **750** | **3L+3P** | **750** | **4L+2P** | **1000** | **5L+1P** | **1250** |
| **>=60 kg** | **3L+3P** | **750** | **4L+2P** | **1000** | **5L+1P** | **1250** | **6L+0P** | **1500** |

***For subjects in the 11 mg/kg group who are 48-59 kg, a dose of 2 tablets (500 mg) would result in these subjects receiving less than the standard-of care levofloxacin dose of 11 mg/kg/day; therefore all subjects assigned to the 11 mg/kg group will receive 3 tablets (750 mg).**

**Levofloxacin will be administered in conjunction with the optimized background regimen. The optimized background regimen will be composed by study and collaborating physicians, using drugs procured through the program’s routine procurement mechanism. Regimens will contain levofloxacin plus at least 3 additional drugs which have documented or expected activity against the patient’s *M. tuberculosis* isolate. If sensitivity cannot be confirmed, and/or there has been prior exposure to drugs included in the regimen, more than 3 drugs should be added (in addition to levofloxacin) to ensure that the regimen contains at least 4 likely effective drugs.^74^**

Treatment with the OBR plus LFX 750 mg will continue after completion of the study period according to local protocol and the discretion of local physicians.

## Modification of Study Intervention/Investigational Product for a Participant

Subject-initiated, provider-initiated and protocol-mandated interruptions include both inadvertent and deliberate interruptions of study drug dose. For the study’s primary endpoint, 200 days (7 days a week) are allowed for completion of at least 168 doses of study medication. Guidelines for clinical evaluation of adverse events and attribution/non-attribution of these events to specific drugs are given in Appendix C. If a non-severe adverse reaction attributable to levofloxacin is suspected, clinicians may elect to give the study drug in two divided doses instead of once daily; if the adverse reaction persists, management should proceed as outlined in the algorithm. If twice daily dosing is requested, the unblinded study pharmacist will prepare the study drug as requested (please refer to MOP for additional details).

For any SAE that is related to the study agent (Definitely, Probably, Possibly; see section 9.2.1), the subject will be strongly advised to discontinue the study levofloxacin regimen.

## Accountability Procedures for the Study Intervention/Investigational Product(s)

The investigator will acknowledge receipt of and keep an inventory of study drug, companion drugs, and matching placebos. Study staff has the responsibility to assure that study drugs are dispensed to patients in compliance with the protocol. Details are available in the MOP and in the Pharmacy Plan.

## Assessment of Subject Compliance with Study Intervention/Investigational Product

Administration of all doses of study and companion drugs will be supervised by study staff and recorded in a log book (see Sections 6.2 and 8.1). Delivery of study medications may occur in health center, hospital, or a place convenient to the subject (home, place of work). The decision will be made jointly between subjects and treatment supervisors.

## Concomitant Medications/Treatments

Concomitant medications, including start dates, stop dates, and dose changes, will be recorded in the medical chart and CRF at each study visit. Study personnel will not be providing direct care for HIV infection in study patients. HIV care will be provided to patient by their primary care clinicians according to provincial and national guidelines. In recognition that HIV care can profoundly influence outcomes for persons infected with both HIV and TB, HIV infected patients will additionally be questioned at each study visit regarding their use of anti-retroviral agents and medicines to treat and prevent opportunistic infections.

Use of known QT-prolonging drugs is prohibited: albuterol, alfuzosin, amantadine, amiodarone, amitryptyline, amphetamine, arsenic trioxide, astemizole, atazanavir, atomoxetine, azithromycin, chloroquine, clomipramine, dolasetron, metaproterenol, phentermine, and phenylpropanolamine.

## OBR (Companion Drugs)

Optimized Background Regimen companion medications include three or more of the standard drugs used for antituberculous chemotherapy for documented drug-resistant tuberculosis. The doses taken will be reported in a DOT log at each visit. According to the 2008 WHO guidelines for the programmatic management of drug-resistant tuberculosis, the standard drugs are:^74^

**Table 5. Drugs for use in treating MDR-TB**

| Group | Description | Drug |
| --- | --- | --- |
| 1 | First-line oral antituberculosis drugs | Ethambutol  Pyrazinamide |
| 2 | Injectable antituberculosis drugs | Kanamycin  Amikacin  Capreomycin |
| 4 | Oral bacteriostatic second-line antituberculosis drugs | Ethionamide  Protionamide  Cycloserine  Terizidone  p-aminosalicylic acid |

As the OBR may include other second-line drugs, the expected adverse reactions and toxicities from these drugs are described below:

- Cycloserine
  - Psychosis
  - Convulsions
  - Depression
  - Headaches
  - Rash
  - Drug interactions
- Ethionamide
  - Gastrointestinal upset
  - Hepatotoxicity
  - Hypersensitivity
  - Metallic taste
  - Bloating
- P-Aminosalicylic acid
  - Gastrointestinal upset
  - Hepatotoxicity
  - Hypersensitivity
  - Sodium load
- Capreomycin
  - Toxicity: Auditory, vestibular, renal
- Kanamycin and amikacin
  - Toxicity: Auditory, vestibular, renal
- Streptomycin

Toxicity: Auditory, vestibular, renal

# Study Schedule

## Screening / Consenting Visit

Subjects with a prior diagnosis of pulmonary tuberculosis at the site of initial diagnosis or TB Clinic will be asked to review and sign the consent form. Subjects will then be screened for eligibility. The following will be performed:

- participants will be asked questions related to demographics, current and past medical history, comorbidities, HIV status, medications they are taking, alcohol, and TB risk factors, past treatment for TB and prior susceptibilities.
- a sputum sample (expectorated) will be obtained for smear and culture at the local site microbiology laboratory. Sputum will be tested for Isoniazid, rifampin, and ofloxacin susceptibility by MTBDRPlus and MTBDR-sl.
- participants will have height and weight measured
- blood will be drawn for a CBC with differential and platelet count, serum or plasma creatinine, ALT, total bilirubin, and blood glucose
- an HIV test, using any testing method approved by the U.S. Food and Drug Administration (or, for non U.S. study sites, approved by local authorities), will be obtained at this visit. unless the patient is known to have HIV infection (documented by a positive ELISA and Western Blot or an HIV-RNA concentration greater than 5000 copies/mL) or has written documentation of a negative HIV test within the past 3 months.
- a urine or serum pregnancy test (if patient is a woman of child-bearing potential)
- Review of concomitant medications.
- Review of previous TB treatment to assess whether subject meets study eligibility criteria.Targeted symptom assessment
- EKG
- Karnofsky score
- Physcial examination
- A posteroanterior chest radiograph will also be taken, unless a posteroanterior chest radiograph done within the previous 14 days is available for review. The chest radiograph must be read prior to study randomization.
- HIV-infected; most recent CD4 count (blood test for CD4 count if result of test done in previous 6 months is not available)

## Randomization/Baseline

Subjects: Patients with pulmonary TB, in whom a rapid test detects rifampin resistance and FQ susceptibility, HIV positive or negative (but not unknown), and weight >40kg will be invited to participate. Written informed consent, using IRB-approved consent forms, will be obtained by trained study personnel prior to performing any study-specific procedures. The informed consent process is described in detail in Section 14.3.

The following will be performed:

- participants will be asked questions related to interval since screening visit history, current and past medical history, comorbidities, HIV status, medications, alcohol, and TB treatment. They will be advised that they can decline to answer any question they consider too personal.
- a sputum sample (expectorated) will be obtained for smear and culture at the laboratory participants will have weight measured
- Blood will be drawn for a CBC with differential and platelet count, serum or plasma creatinine, ALT, total bilirubin, and blood glucose
- a urine or serum pregnancy test (if patient is a woman of child-bearing potential)
- review of medications since screening/consenting visit to assess whether subject continues to meet study eligibility criteria.
- review of interval TB treatment since screening/consenting visit to assess whether subject continues to meet study eligibility criteria.
- Targeted symptom assessment
- Focused clinical assessment – including vitals such as temperature, heart rate, resting rate, and blood pressure
- 10 cc of blood (BD 10 cc Serum Separator Tube (SST) per S29 Biomarker specimen protocol for Biomarker
- At least 20 cc of urine for Biomarker, freeze as soon as possible in -70 degree C.
- EKG
- Karnofsky score

The Enrollment form will be filled out and the data submitted to the study Data Center. The Data Center will then certify that the patient is eligible for randomization. Following this, the Randomization Center will assign the study levofloxacin dose (see section 6.2). Study drug treatment will be initiated during the enrollment visit for participants who meet eligibility criteria. The enrollment visit (and initiation of study drug treatment) should occur as soon as feasible after study treatment assignment. Instructions for administering study drugs are provided in the “Administration of Study Drugs” section. Participants who do not meet eligibility criteria will be referred to a local source of TB care. The study team will work in concert with the local source of TB care to provide timely, medically appropriate TB-related care.

A log will be maintained of persons who are screened, enrolled/refused and withdrawn.

## Follow-up

Visits listed below are protocol-specified study visits. Safety of study participants will be enhanced further by providing study medication doses by directly observed therapy (DOT). In this context, participants will be queried about signs/symptoms potentially related to drug toxicity at the time of each medication dose per standard TB treatment DOT practices. Should signs/symptoms potentially related to drug toxicity be recognized at any time, then further evaluation including laboratory testing may be performed at the discretion of the treating clinician.

Study Visits at:

*Week 1*

- Targeted symptom assessment
- Focused clinical assessment
- Adverse event assessment
- Concomitant medication assessment
- Measurement of weight
- Review DOT log

*Week 2*

- Targeted symptom assessment
- Focused clinical assessment
- Adverse event assessment
- Concomitant medication assessment
- Measurement of weight
- One sputum obtained for culture
- Complete blood count with differential and platelets
- Serum or plasma creatinine, alanine aminotransferase (ALT), total bilirubin
- Review DOT log

*Week 2-4 (one 24 period in this interval)*

- Dietary history based on recall (in-patient & out-patient)
- Seven blood samples for PK evaluation (see section 8.2.2 – may entail inpatient stay)

*Week 3*

- Targeted symptom assessment
- Focused clinical assessment
- Adverse event assessment
- Concomitant medication assessment
- Measurement of weight
- Review DOT log

*Week 4*

- Targeted symptom assessment
- Focused clinical assessment
- Adverse event assessment
- Concomitant medication assessment
- Measurement of weight
- One sputum obtained for culture
- Complete blood count with differential and platelets
- Serum or plasma creatinine, alanine aminotransferase (ALT), total bilirubin, blood glucose
- a urine or serum pregnancy test (if patient is a woman of child-bearing potential)
- EKG
- Review DOT log

*Week 5*

- Targeted symptom assessment
- Focused clinical assessment
- Adverse event assessment
- Concomitant medication assessment
- Measurement of weight
- Review DOT log

*Week 6*

- Targeted symptom assessment
- Focused clinical assessment
- Adverse event assessment
- Concomitant medication assessment
- Measurement of weight
- One sputum obtained for culture
- Complete blood count with differential and platelets
- Serum or plasma creatinine, alanine aminotransferase (ALT), total bilirubin
- Review DOT log

*Week 7*

- Targeted symptom assessment
- Focused clinical assessment
- Adverse event assessment
- Concomitant medication assessment
- Measurement of weight
- Review DOT log

*Week 8*

- Targeted symptom assessment
- Focused clinical assessment
- Adverse event assessment
- Concomitant medication assessment
- Measurement of weight
- One sputum obtained for culture
- Complete blood count with differential and platelets
- Serum or plasma creatinine, alanine aminotransferase (ALT), total bilirubin, blood glucose
- Serum and urine for biomarker studies
- a urine or serum pregnancy test (if patient is a woman of child-bearing potential)
- Review DOT log
- EKG

*Week 10*

- Targeted symptom assessment
- Focused clinical assessment
- Adverse event assessment
- Concomitant medication assessment
- Measurement of weight
- One sputum obtained for culture
- Review DOT log

*Week 12*

- Targeted symptom assessment
- Focused clinical assessment
- Adverse event assessment
- Concomitant medication assessment
- Measurement of weight
- One sputum obtained for culture
- Complete blood count with differential and platelets
- Serum or plasma creatinine, alanine aminotransferase (ALT), total bilirubin, blood glucose
- a urine or serum pregnancy test (if patient is a woman of child-bearing potential)
- Review DOT log
- EKG

*Week 16*

- Targeted symptom assessment
- Focused clinical assessment
- Adverse event assessment
- Concomitant medication assessment
- Measurement of weight
- One sputum obtained for culture
- Complete blood count with differential and platelets
- Serum or plasma creatinine, alanine aminotransferase (ALT), total bilirubin, blood glucose
- a urine or serum pregnancy test (if patient is a woman of child-bearing potential)
- Review DOT log
- EKG

*Week 20*

- Targeted symptom assessment
- Focused clinical assessment
- Adverse event assessment
- Concomitant medication assessment
- Measurement of weight
- One sputum obtained for culture
- Complete blood count with differential and platelets
- Serum or plasma creatinine, alanine aminotransferase (ALT), total bilirubin, blood glucose
- A urine or serum pregnancy test (if patient is a woman of child-bearing potential)
- Review DOT log
- EKG

*Week 24*

- Targeted symptom assessment
- Focused clinical assessment
- Adverse event assessment
- Concomitant medication assessment
- Measurement of weight
- One sputum obtained for culture
- Complete blood count with differential and platelets
- Serum or plasma creatinine, alanine aminotransferase (ALT), total bilirubin, blood glucose
- a urine or serum pregnancy test (if patient is a woman of child-bearing potential)
- Review DOT log
- EKG

**MDR-TB treatment following completion of study phase:** Following completion of the study, subjects will continue OBR according to local protocol, and the blinded levofloxacin dose will be replaced by the standard fluoroquinolone in use for MDR-TB treatment at that site at the standard recommended dose. The study will not provide OBR for either the study phase or the post-study phases of MDR-TB treatment, nor will it provide fluoroquinolone for the post-study phase of MDR-TB treatment.

## Final Study Visit

The final study visit will occur 4 weeks after completion of study phase therapy. Assessments at this visit will include:

- Targeted symptom assessment
- Focused clinical assessment
- Adverse event assessment
- Concomitant medication assessment
- Measurement of weight
- One sputum obtained for culture
- Complete blood count with differential and platelets
- Serum or plasma creatinine, alanine aminotransferase (ALT), total bilirubin

## Early Termination Visit

For study subjects who withdraw from the study treatment early, a final study visit will be performed 4 weeks after termination, with the same items as the Final study visit, unless the subject withdraws consent for all further participation.

## Unscheduled Visit

Unscheduled visits can be initiated by either patient or provider and will be recorded on the “Unscheduled visit” form.

# Study Procedures/Evaluations

Study subjects will be compensated for their time and travel to and from study visits. The form and amount of compensation will be in accordance with local guidelines and subject to local IRB approval.

## Clinical Evaluations

Enrolled patients will have directly observed therapy (DOT) every day for 24 weeks (168 doses). DOT will be observed by study staff or trained community observers and documented in a log book. Study visits will be weekly during the first 8 weeks, at 10 weeks, 12 weeks and then monthly at weeks16, 20, 24, and 4 weeks after the end of treatment with study drug. At each study visit, a focused clinical assessment – including vitals such as temperature, heart rate, resting rate, and blood pressure – will be conducted and evidence of any toxicities obtained by interview and examination. Patient height will be documented at enrollment and patient weight will be assessed at enrollment and at all study visits. Standardized questions on toxicity and tolerability will be posed to each patient at study visits and recorded in source documentation and on the Follow-up Visit Form.

**Review of symptoms since last visit:** Visits listed in Appendix A-Schedule of Events are protocol-specified study visits. At each study visit, a symptom assessment will be conducted. Patients will be queried about targeted symptoms potentially related to drug toxicity at each follow-up visit. Patients will be asked whether they have experienced any of the following since the last study visit: fevers, sweats, cough, rash, itching, phototoxicity, jaundice, nausea, vomiting, diarrhea, abdominal pain, loss of appetite, numbness/tingling of extremities, headache, dizziness, insomnia, joint pain, musculo-skeletal pain or tendon rupture, or new infections,. In addition, patients will be asked whether they have had other symptoms that are not listed above. The reported symptoms will be recorded on the Targeted Symptom Assessment page of the patient’s CRF. Should symptoms potentially related to drug toxicity be recognized at any follow-up visit, further evaluation including laboratory testing may be performed at the discretion of the treating clinician. Furthermore, the patients will be assessed for concomitant medications, including warfarin, theophylline, benzodiazepines, and phenylpropanolamine.

## Laboratory Evaluations

### Clinical Laboratory Evaluations

A pregnancy test will be done during screening, enrollment, and at weeks 4, 8, 12, 16, 20, and 24. In addition, HIV serology, and a chest X-ray will be undertaken at the enrollment visit. Enrollment and study visits during weeks 2, 4, 6, 8,12,16, 20 and 24 will include obtaining a complete blood count with differential and platelets, specified blood chemistries (serum or plasma creatinine, ALT, and total bilirubin) and a directed physical examination,. An expectorated spot sputum sample will be obtained at enrollment and weeks 2, 4, 6, 8, 10, 12, 16, 20, and 24. If a first negative culture is obtained at week 24, a specimen will also be collected at week 28 to assess conversion at week 24. EKG readings will be taken during weeks 4, 8, 12, 16, 20 and 24. In this study, we will also collect serum and urine from all participants at enrollment and at week 8, for storage as part of future biomarker discovery and qualification sub-studies.

**Obtaining sputum specimens:** A single spontaneously expectorated sputum will be collected at screening, enrollment (day 1), 2 weeks, 4 weeks, 6 weeks, 8 weeks, 12 weeks, and monthly thereafter through week 24, and at four weeks after the end of study regimen. A minimum of 5 ml of sputum must be collected up through week 4, and a minimum of 3 ml at subsequent time points. A sputum specimen will be defined as unobtainable (and negative for analytic purposes) if no sputum can be obtained.

**Laboratory aspects of sputum analysis:** For each study site, a single laboratory will be designated as the site’s study laboratory, and all study-related sputum specimens must be sent to that laboratory. Specimens should be delivered to the laboratory on the same day of collection. Specimens must be stored in the refrigerator and transported in a cool box if delivery to the laboratory is >2 hours from the time of collection. For details of processing of sputum specimens see study laboratory manual of procedures.

Sputum specimens will be used for AFB smears, solid culture on Middlebrook 7H11S

medium, and liquid culture using the MGIT® 960 system. The identification of *M. tuberculosis* (MTB) complex will be performed at the site laboratory for the first positive culture only. The time-to-positive (TTP) culture in the MGIT system will be determined for all MTB complex positive MGIT cultures. All microbiology tests will be done in accordance with Clinical Laboratory Standards Institute guidance for sputum processing, smear microscopy, culture techniques, and identification of mycobacteria (Guidance documents M22-A3, M24-A, and M48-P). AFB smears will be scored by the World Health Organization (WHO) standard scale (Negative, scanty positive, 1+, 2+, 3+).

**Drug Susceptibility Testing:** Susceptibility testing of first-line (INH, rifampin, ethambutol, pyrazinamide) and second-line drugs, including ofloxacin, will be done on *M. tuberculosis*- positive cultures of specimens collected at screening visit per local protocols. Isolates from screening visit and last positive culture (if after month 3) will be sent to CDC at the conclusion of the study. At CDC, MICs for levofloxacin on enrollment specimen and final isolate (for those subjects still culture positive at month 4 or later) will be performed by the method of Heifets, et al.^76^ CDC will also perform in vitro PZA susceptibility testing on all screening or baseline isolates.

### Special Assays or Procedures

**Pharmacokinetic sampling:** sampling will take place in one 24 hour period between the 14^th^ day through the 28^th^ day from the start of study regimen. Depending on the site, this may entail an overnight inpatient stay for study participants. On the day of PK sampling, study drugs will be administered in the morning at the time the patients usually take their dose. Study drugs will be swallowed with 200 cc of water after being NPO for 8 hours and no food will be ingested for the next two hours. A breakfast and light meal will be provided two and six hours after administration of study drugs. The timing and content of meals, snacks, and concurrent medications taken by each subject on the day of the pharmacokinetic study will be recorded.

The blood specimen will be obtained at enrollment prior to the ingestion of drugs. All blood samples will be collected in reference to a single directly observed dose of study phase drugs. The reference dose used for PK sampling should be preceded by two daily study drug doses given approximately 24 hours apart during the preceding 48 hours. Patients who experience emesis during the course of the pharmacokinetic sampling and vomit within 2 hours of dosing will be rescheduled for another PK sampling within two weeks. Patients should abstain from consuming any alcohol for 48 hours prior to the PK study period and until after the last PK sample is collected.

Five ml of venous blood for determination of levofloxacin plasma concentrations will be collected before dosing (time 0) and at 1, 2, 4, 8, 12, and 24 hours after administration of the morning dose of study drug treatment on one day between days 14 and 28 of treatment. Patients will have received a minimum of 3 consecutive daily doses prior to the blood collections.

Participants will be interviewed to obtain additional information about medical and social history, recent weight loss or weight gain, concomitant medications on the day prior to and the days of PK sampling, gastrointestinal symptoms, and timing of meals, snacks and medications relative to study drug dosing.

PK samples will be temporarily stored in the local laboratory before being shipped to the University of Florida for PK analysis.

**HIV testing:** Patients will have HIV testing performed at study entry unless such testing has been previously documented to be positive or has been performed and documented to be negative within the previous 3 months. HIV test results will be given only to the patient (and to those to whom it may be required by law) and kept with other study records in a secure place. With their permission, HIV-infected patients will be referred to local sources of HIV care, as appropriate. HIV-indeterminate patients will be given a follow-up appointment for re-testing.

HIV-infected study subjects who are eligible for antiretroviral regimen according to national guidelines pertaining to their study site should be referred to local sources of care for such regimen. The study is not performing CD4 counts as part of the study protocol except at the randomization visit. The study protocol allows the co-administration of antiretroviral agents when clinically indicated. Patients who have antiretroviral regimen initiated during the study phase should be observed closely for the possible occurrence of severe immune reconstitution syndromes.

**Management of subjects enrolled but subsequently determined to have isolates that are rifampin or isoniazid susceptible or ofloxacin-resistant based on culture and susceptibility results:** Because of the relatively slow growth of *M. tuberculosis* in culture, the results of sputum mycobacterial cultures and phenotypic drug susceptibility testing are rarely available at the time of initiation of MDR TB treatment. Therefore for this study we plan to enroll patients based on the result of the MTBDRplus and MTBDRsl tests. Patients whose baseline or screening cultures demonstrate isoniazid or rifampin susceptibility or ofloxacin resistance will be discontinued from the study treatment and referred to the local TB control program for treatment with a regimen appropriate for their disease. Similarly, patients whose screening and baseline cultures are both negative will be discontinued from the study treatment and referred to the local TB control program for treatment with a regimen appropriate for their disease. Such patients will not be included in the modified intention-to-treat study population. However, all such patients will have a study visit at 4 weeks after discontinuation of the study regimen for assessment of delayed toxicity and will be included in the safety database.

### Specimen Preparation, Handling, and Shipping

#### Instructions for Specimen Preparation, Handling, and Storage

These instructions can be found in the study MOP

#### Specimen Shipment

- Specimens for the Central Mycobacteriology Laboratory will be shipped to the CDC, Atlanta GA.
- Specimens for the Central Clinical Pharmacology laboratory will be shipped to the University of Florida in Gainesville FL
- Specimens for the Central Specimen Repository will be shipped to the University of California, San Francisco, CA, USA.

Detailed instructions can be found in the study MOP.

# Assessment of Safety

## Specification of Safety Parameters

**Primary Safety Endpoint** – the primary safety endpoint will be the number of grade 3, 4 and 5 adverse events (AEs), occurring up to and including the time on study drug plus four weeks post study drug completion.

## Methods and Timing for Assessing, Recording, and Analyzing Safety Parameters

### Adverse Events

**Adverse Event:** ICH E6 defines an AE as any untoward medical occurrence in a patient or clinical investigation subject administered a pharmaceutical product regardless of its causal relationship to the study treatment. An AE can therefore be any unfavorable and unintended sign (including an abnormal laboratory finding), symptom, or disease temporally associated with the use of medicinal (investigational) product. The occurrence of an AE may come to the attention of study personnel during study visits and interviews of a study recipient presenting for medical care, or upon review by a study monitor. If the primary care team suspects an adverse event between study visits, this will trigger an unscheduled study visit to assess the potential adverse event.

All AEs will be captured on the Adverse Event (AE) Case Report Form (CRF). Information to be collected includes event description, time of onset, clinician’s assessment of severity, relationship to study product (assessed only by those with the training and authority to make a diagnosis, which would include MD, PA, Nurse Practitioner, DO, or DDS), and time of resolution/stabilization of the event. All AEs occurring while on study must be documented appropriately regardless of relationship. All AEs will be followed to adequate resolution.

Any medical condition that is present at the time that the patient is screened should be considered as baseline and not reported as an AE. However, if it deteriorates at any time during the study, it should be recorded as an AE.

All AEs must be graded for severity and relationship to study product.

FDA defines an AE as any untoward medical occurrence associated with the use of a drug in humans, whether or not considered drug related.

A serious adverse event is defined as an AE meeting one of the following conditions:

- Death during the period of protocol defined surveillance
- Life threatening event (defined as a subject at immediate risk of death at the time of the event)
- An event requiring inpatient hospitalization or prolongation of existing hospitalization during the period of protocol defined surveillance
- Results in congenital anomaly or birth defect
- Results in a persistent or significant incapacity or substantial disruption of the ability to conduct normal life functions.

Any other important medical event that may not result in death, be life threatening, or require hospitalization, may be considered a serious adverse experience when, based upon appropriate medical judgment, the event may jeopardize the subject and may require medical or surgical intervention to prevent one of the outcomes listed above. Examples of such medical events include allergic bronchospasm requiring intensive treatment in an emergency room or at home, blood dyscrasias or convulsions that do not result in inpatient hospitalization, or the development of drug dependency or drug abuse.

All serious adverse events will be:

• Recorded in a timely and proper manner, and reported to the relevant entities.

• Followed through resolution or stabilization by a study clinician

• Reviewed and evaluated by a study clinician

**Severity of Event:** The severity of each adverse event will be determined using the Common Terminology Criteria for Adverse Events v3.0 (CTCAE) published August 9, 2006 which can be found at http//ctep.cancer.gov/forms/CTCAEv3.pdf. Any events that are not listed in this toxicity table will be graded by the local PI as follows:

| Grade 1 | Mild | Asymptomatic or mild symptoms; clinical or diagnostic observations only; intervention not indicated. |
| --- | --- | --- |
| Grade 2 | Moderate | Minimal, local or noninvasive intervention indicated; limits age-appropriate instrumental ADL. |
| Grade 3 | Severe | Medically significant but not immediately life-threatening; hospitalization or prolongation of hospitalization indicated; disabling; limits self-care ADL. |
| Grade 4 | Life-threatening | Consequences; urgent intervention indicated. |
| Grade 5 | Death | Related to AE. |

**Relationship to Study Products:** Any AE that occurs in a patient will be assessed for relationship to the study drug as outlined in Appendix C. A causal relationship means that the drug caused (or is reasonably likely to have caused) the AE. This usually implies a relationship in time between the drug/study agent and the AE⎯for example, the AE occurred shortly after the patient received the drug/study agent.

Whether to re-challenge the patient with study drug or not will be left to the discretion of study staff in combination with the primary providers for the patient in conjunction with the patient. Re-challenges will be documented by recording when patient resumed study drug and any subsequent AE’s. Experience from re-challenge may be used for design of a re-challenge protocol for a subsequent larger study, if it is warranted based on the results of this pilot study.

For all AEs, the principal investigator or his/her designee who examines and evaluates the patient will determine the adverse event’s causality based upon the temporal relationship to administration of the study agent(s), the pharmacology of the study agent(s), and his/her clinical judgment.

The following scale will be used to reflect the PI’s or his/her designee’s judgment as to the relationship between the study agent/intervention and the AE:

- Definitely Related: The AE is clearly related to the study agent – follows a reasonable temporal sequence from administration of the study agent, follows a known or expected response pattern to the study agent that is confirmed by improvement on stopping or worsening/reappearance of the event in repeated exposure and that could not be reasonably explained by the known characteristics of the patient’s clinical state.
- Probably Related: The adverse event and administration of study agent are reasonably related in time or follows a known pattern of response, and the adverse event is more likely explained by study agent than other causes.
- Possibly Related: AE follows a reasonable temporal sequence from administration of the study drug/intervention, follows a known or expected response pattern to the suspected drug, but that could readily have been produced by a number of other factors.
- Probably not Related: A potential relationship between study agent and the adverse event could exist (i.e., the possibility cannot be excluded), but the adverse event is most likely explained by causes other than the study agent (e.g., could readily have been produced by the patient’s clinical state or could have been due to environmental or other interventions)
- Not Related: AE is clearly not related to the study agent – another cause of the event is most plausible or a clinically plausible, temporal sequence is consistent with the onset of the event and the study medication administration or event is biologically implausible.

### Procedures to be Followed in the Event of Abnormal Laboratory Test Values or Abnormal Clinical Findings

Changes in laboratory values are reported as AEs if they are clinically significant (i.e., if some action or intervention is required or if the investigator judges the change to be beyond the range of normal physiological fluctuation) or if they are a change in grade as indicated in the Common Terminology Criteria for Adverse Events. Should a patient call a study clinician to report an adverse event, it will be determined at that time if an extra visit(s) will be scheduled, and appropriate medical advice will be provided. All actions taken by the investigator after observing an AE should be documented, including increased monitoring of the patient/study participant, suspension of the treatment, etc. Additionally, all calls will be documented in the patient/study participant’s study chart, and discussed with the principal investigator.

All serious and non-serious AEs reported in this study will be followed until resolution or until the investigator and the treating physician are in agreement that the AE has stabilized and no more follow-up is required. This requirement indicates that follow-up may be required for some events after the patient discontinues participation from the study.

In the event that the patient’s study medication is stopped due to an AE, it must be recorded on the CRF as such. The patient should be followed and treated by the investigator until the abnormal parameter or symptom has resolved or stabilized. It is up to the clinician to determine that the AE is either resolved, or that it has reached a stable state, after which no further follow-up is necessary. There should be documentation to support that determination.

## Reporting Procedures

Any AE, whether or not considered related to the study drug, or whether considered non-serious or unexpected, that occurs between the times a patient signs the informed consent form and the time s/he departs the study (for any reason) must be fully and completely documented on the Adverse Event (AE) Case Report Form (CRF) and in the patient/study participant’s clinical chart. The start date, the stop date, the severity of each reportable event, and the principal investigator’s or his/her designee’s judgment of the AEs relationship to the study agent/intervention will also be recorded on the Adverse Event CRF.

**Internal Reporting Requirements:** All deaths and life-threatening SAEs which occur during the course of this investigation, whether or not considered to be related to the study agent or considered to be an expected event associated with the study agent, will result in a preliminary report that is submitted the Independent Safety Monitor (ISM) with a copy to the DMID and study sponsor’s pharmacovigilance group **within 24 hours of awareness**. The ISM will provide an independent written assessment of the SAE to the DMID Medical Monitor and study sponsor’s pharmacovigilance group within 2 business days after receipt of this preliminary report. This reporting timeline has been determined to account for the time it may take the treating doctors working with the patients to report to the local study staff who in turn will report to DMID.

Adverse event reporting requirements for this protocol are as follows:

1. Local Investigators must provide a preliminary report to local IRB after becoming aware of a patient death or a potentially life-threatening serious adverse event. This preliminary report must be followed by a progress report within 8 days.
2. Investigators must provide a final report to the local IRB and regulatory authorities within 15 days after becoming aware of an inpatient hospitalization (other than elective), a persistent or significant disability/incapacity, or a congenital anomaly/birth defect.

### Serious Adverse Events

All SAEs which occur during the course of this investigation, whether or not considered to be related to the study agent or considered to be an expected event associated with the study agent, will result in a preliminary report that is submitted within 24 hours of site awareness to the ISM, Pharmacovigilance Group of Macleods Pharmaceuticals at [safety@macleodspharma.com](mailto:safety@macleodspharma.com) and the DMID Pharmacovigilance Group, at the following address:

DMID Pharmacovigilance Group

Clinical Research Operations and Management Support (CROMS)
6500 Rock Spring Dr. Suite 650

Bethesda, MD 20814, USA

SAE Hot Line: 1-800-537-9979 (US) or 1-301-897-1709 (outside US)

SAE FAX Phone Number: 1-800-275-7619 (US) or 1-301-897-1710 (outside US)

SAE Email Address: [PVG@dmidcroms.com](mailto:PVG@dmidcroms.com)

The DMID medical monitor and clinical protocol manager will be notified of the SAE by the DMID Pharmacovigilance Group. The DMID medical monitor will review and assess the SAE for regulatory reporting and potential impact on study subject safety and protocol conduct.

At any time after completion of the study, if the investigator becomes aware of an SAE that is suspected to be related to study product, the investigator will report the event to the DMID Pharmacovigilance Group.

Note: All AEs, including SAEs, occurring within the reporting period will be documented on the appropriate Case Report Forms (CRFs). For the purposes of adverse event reporting, hospitalization for the protocol-specified PK evaluation will not be a reportable SAE.

### Regulatory Reporting for Studies Not Conducted Under DMID‑Sponsored IND

This study will be conducted under an IND held by the product manufacturer, Macleods pharmaceuticals, Ltd. Any AE that meets the protocol-defined serious criterion must also be submitted within 24 hours of site awareness to MacLeods Pharmaceuticals, Ltd. At any time after completion of the study, if the investigator becomes aware of an SAE that was not previously reported, the investigator will also report this event to MacLeods Pharmaceuticals, Ltd.

### Reporting of Pregnancy

Pregnancy is not a contraindication to levofloxacin treatment for MDR-TB, but the safety of treatment with levofloxacin at investigational doses in pregnancy cannot be assured. Therefore, study subjects who become pregnant during the trial will have their study treatment discontinued and replaced with standard doses of levofloxacin. Subjects who become pregnant will be followed for the duration of the pregnancy and infants followed for 6 months after birth. This will be reported to the sponsor, study leadership, IRB, and regulatory agencies.

## Type and Duration of Follow-up of Subjects after Adverse Events

AEs will be followed until resolution or stabilization even if this extends beyond the study-reporting period. Resolution of an AE is defined as the return to pretreatment status or stabilization of the condition with the expectation that it will remain chronic.

## Guidance for Temporary Discontinuation of Study Drug

When it is unclear whether the AE is associated with levofloxacin, study levofloxacin/placebo may be discontinued while re-challenge with other drugs or study levofloxacin/placebo is performed (See Appendix C: Toxicity Management Algorithm). If it is determined that study levofloxacin/placebo was not the cause of the AE, it should be restarted. Restarting the daily study dose as half dose twice daily is acceptable and will be considered to be continuation of the protocol-assigned dose. Such temporary discontinuation will not be considered a study endpoint unless the duration of the discontinuation makes it impossible for the patient to complete the required 168 doses in 200 days.

## Safety Oversight

### Independent Safety Monitor (ISM)

An ISM will be appointed at each study site.

The ISM is a physician located near the investigator site with relevant expertise whose primary responsibility is to provide independent safety monitoring in a timely fashion. This ISM is identified by the investigator site and approved by the DMID. The ISM will review SAEs and other AEs as needed and provide an independent assessment to DMID. The study site will have an ISM with experience in infectious diseases and/or internal medicine.

### Data and Safety Monitoring Board (DSMB)

DMID will organize the DSMB. The Data Coordinating Center (DCC) will provide the appropriate reports for DSMB review. The DSMB will meet semiannually to assess safety and efficacy data on each arm of the study.

The DSMB will also review aggregate safety data for increased rate of occurrence of serious suspected adverse reactions. If more than 50% of patients in any dose arm have permanently discontinued treatment for any reason before completing 24 weeks of treatment at the time of review, the DSMB may recommend that enrollment in that arm be terminated. In addition to the semiannual meeting, the DSMB will review safety and tolerability of all doses three months after 40 patients have been enrolled and again three months after 60 patients have been enrolled. The DSMB will advise DMID of its recommendations after each meeting.

### Halting Rules

In addition to the above, subsequent review of serious, unexpected, and related AEs by the Medical Monitor, DSMB, IEC/IRB, the sponsor(s), or the FDA or relevant local regulatory authorities may result in suspension of further trial interventions/administration of study product at a site. The FDA and study sponsor(s) retain the authority to suspend additional enrollment and study interventions/administration of study product for the entire study, as applicable.

# Clinical Monitoring

## Site Monitoring Plan

Under the U01 mechanism, site monitoring is the responsibility of the investigators. To ensure that human subject protection, study procedures, laboratory procedures, study intervention administration, and data collection processes are of high quality and meet sponsor, CGP/ICH, and regulatory guidelines, and that the study is conducted in accordance with the protocol and sponsor SOPs, the study will be monitored by Westat, Inc.

In brief, site visits will be made at standard intervals as defined by the Site Monitoring Plan. Monitoring visits will include, but are not limited to, review of regulatory files, accountability records, case report forms, informed consent forms, medical and laboratory reports, and protocol compliance. Study monitors will meet with investigators to discuss any problems and actions to be taken and document visit findings and discussions. See detailed site monitoring plan for details. The details of the site monitoring process for this study are given in a separate document (Site Monitoring Plan)

# Statistical Considerations

## Study Hypotheses

Hypothesis 1a: Increasing AUC/MIC of levofloxacin will be associated with shorter time to sputum culture conversion

Hypothesis 2a: Increasing AUC of levofloxacin will be associated with increased proportion of Subjects with Grade 3 or 4 Adverse Events

Hypothesis 2b: Increasing AUC of levofloxacin will be associated with decreased frequency of completion at assigned dose.

Hypothesis 3. An algorithm including patient age, sex, race, height, weight, and creatinine will accurately predict serum levofloxacin AUC.

## Sample Size Considerations

*Hypothesis 1a. (Efficacy; continuous exposure, continuous outcome).*

The sample size should be sufficient to test the first hypothesis that there is an association between AUC/MIC and time-to-treatment-success. A total of 62 evaluable patients will allow a correlation of -0.40 to be detected with 90% power. Ρ=-0.40 is considerable to be moderate correlation.

This sample size calculation is based on the Pearson correlation coefficient (ρ) between two normally distributed variables (the ratio AUC/MIC and log time to sputum culture conversion) and the null hypothesis to be tested is H_0_: ρ=0. The calculations use the Fisher transformation of the correlation coefficient and the expected time to conversion with the lowest dose of levofloxacin as equal to that observed by Holtz.[8] In the Holtz study, 50% of patients with MDR-TB converted their sputum culture to negative on solid medium by 2 months and 75% had converted by 3 months; therefore, we have planned more frequent culture sampling during weeks 0-12 (every 2 weeks) and less frequent sampling during weeks 13-24 (every 4 weeks).

Table 6 shows the required evaluable patients to detect the specified Pearson correlation coefficient (ρ) with the specified power, based on a two-sided significance concentration of 5% (α=0.05).

**Table 6. Required number of evaluable patients to detect specified Pearson correlation coefficient with specified power**

| ρ | Power | | | |
| --- | --- | --- | --- | --- |
|  | 80% | 85% | 90% | 95% |
| -0.35 | 62 | 71 | 82 | 101 |
| -0.40 | 47 | 54 | 62 | 76 |
| -0.45 | 37 | 42 | 40 | 59 |
| -0.50 | 30 | 33 | 38 | 47 |

*Hypothesis 2a (Safety; continuous exposure, dichotomous outcome).*

The sample size calculation is based on the same methodology and estimates of mean AUC as for the tolerability endpoint below (Hypothesis 2b). It is informative to estimate what power will be available with 79 evaluable subjects to test whether there is a relationship between the AUC and whether a patient has experienced a grade 3, 4 or 5 adverse events (AEs), occurring up to and including the time on study drug plus four weeks post study drug completion. Table 7 shows the detectable odds ratio with 79 evaluable subjects under 80% and 90% power using a two-sided significance level of 5% (α=0.05). With 79 evaluable subjects, therefore, if the proportion with mean AUC having a grade 3, 4 or 5 AE is 30% (p1), there will be 80% power to detect an association if the proportion at mean AUC + 1 SD is 46% (p2) or 90% power if the proportion is 49%.

**Table 7. Detectable odds ratio with 79 evaluable subjects**

| Power | p1  Proportion with mean AUC having a grade 3, 4 or 5 AE | p­2  Proportion with mean AUC + 1 SD having a grade 3, 4 or 5 AE | Detectable Odds Ratio |
| --- | --- | --- | --- |
| 80% | 0.20 | 0.35 | 0.46 |
|  | 0.25 | 0.41 | 0.48 |
|  | 0.30 | 0.46 | 0.50 |
|  | 0.35 | 0.51 | 0.52 |
|  | 0.40 | 0.56 | 0.53 |
| 90% | 0.20 | 0.38 | 0.40 |
|  | 0.25 | 0.44 | 0.43 |
|  | 0.30 | 0.49 | 0.45 |
|  | 0.35 | 0.54 | 0.46 |
|  | 0.40 | 0.58 | 0.47 |

*Hypothesis 2b (Tolerability; continuous exposure, dichotomous outcome).*

The sample size should also be sufficient to test the second hypothesis that there is a relationship between the AUC and whether a patient has completed 6 months of treatment at the assigned dose. It is expected that the proportion of treatment completion at the mean will be 85% and 70% when the AUC increases by one standard deviation (SD). A total of 79 evaluable patients will therefore be required to detect this difference (Table 8).

The sample size calculation is based on the logistic regression of a binary response variable (whether the patient has completed 6 months of treatment with the assigned dose) on a continuous, normally distributed variable (AUC). The null hypothesis to be tested is H_0_:OR=1 where OR is the odds ratio of whether a patient has completed 6 months of treatment at different concentrations of AUC. The sample size calculation is based on the normal approximation formulas given in Hsieh FY, et al..^77^

Table 8 shows the required evaluable patients to detect the specified odds ratio under various conditions, with a one-sided significance concentration of 5% (α=0.05) and power of 80%.

Based on previously published data,^72-73^ the estimated mean AUC will be approximately 140 mg, with a standard deviation of about 65 mg. p_1_ corresponds to the proportion of those with mean AUC (140mg) completing treatment and p2 corresponds to the proportion of those with mean + 1 SD (205mg) completing treatment.

**Table 8. Required number of evaluable patients to detect specified odds ratio**

| p_1_  Proportion with mean AUC completing treatment | p­_2_  Proportion with mean AUC + 1 SD completing treatment | Detectable Odds Ratio | Evaluable patients |
| --- | --- | --- | --- |
| 0.50 | 0.35 | 0.538 | 82 |
| 0.50 | 0.40 | 0.667 | 191 |
| 0.60 | 0.45 | 0.545 | 90 |
| 0.60 | 0.50 | 0.667 | 199 |
| 0.75 | 0.60 | 0.500 | 88 |
| 0.75 | 0.65 | 0.619 | 183 |
| 0.85 | 0.70 | 0.412 | 79 |
| 0.85 | 0.75 | 0.529 | 153 |
| 0.95 | 0.80 | 0.211 | 69 |
| 0.95 | 0.85 | 0.298 | 113 |

Summary

It is expected that as many as 25% of enrolled subjects will be lost to follow-up with regard to the efficacy endpoint, based on experience in other MDR-TB clinical trials of this design (McNeeley, David, personal communication). For the tolerability endpoint, it is expected that a total of 20% (20) of the enrolled patients will be lost to follow-up, since subjects who stop their study drug dose because of intolerability will have met the endpoint for tolerability and will thus not be “lost” with regard to this endpoint. For endpoint 2, we will enroll 100 subjects, yielding 80 evaluable subjects. For endpoint 1, 100 patients with 25% Lost to follow-up will yield 75 evaluable subjects, greater than the 62 required. The sample size calculations are meant to take into account additional patients enrolled then excluded with fluoroquinolone-resistant TB. However, if the number of such patients is such that the lost to follow up proportion exceeds 25%, additional subjects will be enrolled to replace those who have fluoroquinolone-resistant TB that is misclassified by MTBDRsl. This sample size will give:

- 95% power to evaluate hypothesis 1 assuming a correlation of -0.4 or 88% power assuming a correlation of -0.35.
- 80% power to evaluate hypothesis 2a assuming that the proportion of Grade 3,4 or 5 AEs at the mean will be 30% and 54% when the AUC increases by one SD.
- 80% power to evaluate hypothesis 2b assuming that the proportion of treatment completion at the mean will be 85% and 70% when the AUC increases by one SD.

Aim 3

It is not straightforward to determine how many patients will be required to develop a dosing algorithm to achieve the AUC associated with maximal efficacy and acceptable safety and tolerability (Aim 3). Experience from other similar studies can, however, give information about likely sample sizes required.

A demographic prediction model linking demographic characteristics of patients taking a single dose of levofloxacin with pharmacokinetic parameters has been previously developed.^73^ In that study, the authors used data from 172 patients to develop the model and 100 patients to train the model. In the training data set of only 100 patients, the resulting model was relatively robust for predicting clearance from plasma for levofloxacin, a proxy for AUC, R^2^ = 0.40. Dose of treatment is likely to be a much stronger predictor of AUC than demographic characteristics and therefore it is likely that the 80 evaluable patients that will be available in the present study will be sufficient to develop a dosing algorithm for aim 3 including important covariates in the prediction model.

## Planned Interim Analyses (if applicable)

**Adaptive Design to Limit Intolerance**

The Data and Safety Monitoring Board (DSMB) will review tolerability of all doses three months after 40 patients have been enrolled and again three months after 60 patients have been enrolled. If more than 50% of patients in any dose arm have permanently discontinued treatment for any reason before completing 24 weeks of treatment, the DSMB will recommend that enrollment in that arm be terminated. The total sample size will remain the same, and patients subsequently enrolled into the trial will be randomized equally between the remaining study arms.

## Final Analysis Plan

**Analysis Plan of Primary objectives**

Patients who complete study drug treatment will be included in the per protocol analysis. The primary analyses will be conducted on all randomized patients that have received at least one dose of trial medication, applying the principle of intention to treat (ITT), as far as is practically possible, given any missing data. There will, however, be a small number of subjects lost post-randomization because of anticipated delays in availability of testing for isoniazid, rifampin and ofloxacin resistance and subjects with no positive culture for *M. tuberculosis* from samples taken at screening or randomization. Thus, a modified intention to treat (MITT) analysis will be undertaken with the MITT population defined as subjects randomized with a positive culture at screening or baseline, phenotypic resistance to isoniazid and rifampin, and phenotypic susceptibility to ofloxacin. This MITT population will be used for evaluation of both efficacy and tolerability (but not safety). The results will be adjusted for creatinine and HIV status.

*1. Efficacy* The primary efficacy outcome is time to culture conversion on solid media as defined in section 3.2.1. The primary efficacy exposure is the ratio of AUC/MIC. It is expected that time to culture conversion follows a log-normal distribution^8^ and therefore times will be log-transformed. The patient’s levofloxacin exposure (AUC) as assessed by pharmacokinetic measurements divided by the minimum inhibitory concentration of levofloxacin required to kill 90% of the patient’s isolate (AUC/MIC_90_) will be the exposure variable. Because there could still be as much as one tube dilution error in the assay, which could lead to wide variation in AUC/MIC ratios, we will perform the MIC assays in triplicate and average the findings, giving us increased precision and avoiding the misclassification introduced by rounding to the nearest power of 2. Log transformation will also be explored as a method for smoothing the exposure values. Linear regression will be used to calculate the Pearson correlation coefficient between AUC/MIC and log-transformed time to culture conversion. The relationship will be plotted on a scatter plot of AUC/MIC against time to culture conversion curve on the log scale. Missing times for patients that do not culture convert and missing AUC/MIC will be imputed in secondary sensitivity analyses to evaluate the effect of missing data. The primary objective is to determine whether there is sufficient evidence to reject the null hypothesis that the Pearson correlation coefficient is equal to zero.

A time-to event analysis will also be performed. The exposure variable will be the patient’s levofloxacin exposure (AUC) as assessed by pharmacokinetic measurements divided by the minimum inhibitory concentration of levofloxacin required to kill 90% of the patient’s isolate (AUC/MIC_90_), averaged across three assays. We will use time-to-event analysis to evaluate the relationship between the exposure variable and time to conversion; if the proportional hazards assumptions are met, Cox proportional hazards models will be employed to evaluate the crude and adjusted effects. Measured covariates will include site, HIV co-infection, baseline bacillary load (as measured by time to detection in liquid culture), previous MDR treatment and cavitation, and dosing schedule (single daily or split). Since we expect randomization of study subjects to lead to balanced distribution of OBR drugs—including less effective ones—across study arms, we do not expect to need to control for the composition of the OBR. If, however, the distribution is imbalanced, we will adjust for composition of the OBR. If Cox models are used, the effect estimate will be the hazard ratio, which can be interpreted as the change in rate of culture conversion as the AUC/MIC changes by one unit (and all other variables are held constant, in the adjusted analysis). Persons discontinuing levofloxacin due to adverse events will be censored in the time to event analysis of time to culture conversion; they will therefore contribute time until they are censored. This anticipated loss is accounted for in the sample size estimates.

We will also perform a comparison of the responses of persons who have an AUC/MIC o>100to those who have an AUC/MIC ratio of <100. We will first compare these two groups with regard to the time to sputum culture conversion as a continuous outcome using non-parametric tests. We will also dichotomize the outcome (culture conversion by week 12: yes/no) and evaluate the predictive value of a cutoff of 100 using receiver-operator curve analysis, constructing a model that adjusts for other predictors of this outcome, as described by Drusano and colleagues.^60^

*2a. Safety.* Exposure is AUC and the primary safety outcome is the number of grade 3, 4 and 5 adverse events (SAEs), occurring up to and including the time on study drug plus four weeks post study drug completion. The number of events will be plotted against the AUC of the individual in whom those events occurred. Correlation between AUC and number and severity of events will be assessed. The resulting relationship will be used to identify an AUC at which greater than 25% of patients would be expected to have grade 3, 4, or 5 adverse events.

Time to event methods will be used to compare the incidence of SAEs between dosing groups and to evaluate whether SAEs tend to accumulate earlier when higher doses of levofloxacin are taken.

*2b. Tolerability.* The primary tolerability outcome is completion of 24 weeks of study drug regimen as defined in section 3.2.1. The primary tolerability exposure is AUC.

Logistic regression will be used to model the association between these variables with AUC included as a continuous covariate. If the distribution of AUC is skewed then the AUC will be log transformed. Other transformations will be explored as appropriate. The primary objective is to determine whether there is sufficient evidence to reject the null hypothesis that the odds ratio of discontinuation for a unit increase in AUC is equal to 1. Model parameters will also be used to estimate the AUC associated with 25% intolerability.

*3.* Construction of Dosing Algorithms.

*Choice of target.* The target AUC/MIC that produces maximal efficacy and the highest AUC that is associated with acceptable safety and tolerability will be determined from the results of the studies outlined in primary Objectives 1 (efficacy), 2a (safety) and 2b (tolerability) above. The target choice will be accomplished by first examining the shape of the AUC/MIC to time-to culture conversion relationship to see if continued increases in AUC/MIC are associated with continued decreases in time to culture conversion, or if this relationship plateaus (or reverses direction), which would indicate that further increases in AUC would not be associated with increased efficacy. If the relationship shows that the effect of AUC/MIC plateaus, then the preliminary choice of target will be that AUC. If it does not plateau, then the preliminary choice will be the highest achievable AUC. This preliminary target will then be examined for safety and tolerability, defined above as not having a significantly increased proportion of grade 3,4 or 5 AEs and having at least 75% of patients able to tolerate the AUC for 6 months. If the maximal safe dose and the maximal tolerable dose exceed the preliminary target, then the preliminary target will be the primary final target. If not, then the lower of the three (preliminary target, maximal safe dose and maximal tolerable dose) will be the primary final target. If the maximal effective dose exceeds the tolerability threshold but not the safety threshold, then a second algorithm will be constructed to predict the dose likely to achieve the maximal effective threshold. This algorithm may be preferred in clinical situations where the willingness to tolerate side effects (and reduce levofloxacin dose as needed) is greater.

*Construction of algorithms.* We will use linear regression to develop dosing algorithms that will identify the highest safe and tolerable doses of levofloxacin (in mg/Kg) that a given patient should receive to achieve the AUC Target. Analyses by ANCOVA will identify covariates or factors that may affect levofloxacin exposure like drug dose, patient height, weight, age, sex and race. Demographic and clinical terms will be adjusted for drug dosage, and significant factors combined into a multivariable model with interactions of terms in the main effects model assessed for significance.^48^ Results will be adjusted for creatinine clearance, (calculated as previously described^78^ and HIV status and other demographic or clinical variables that are associated with AUC in univariate analyses. The tolerability algorithm will be constructed to yield a distribution of AUC values such that 95% of subjects would be expected to have an AUC equal to or less than the Target AUC. The efficacy algorithm will be constructed to yield a distribution of AUC values such that 95% of subjects would be expected to have an AUC equal to or greater than the Target AUC. Use of these dosing algorithms will facilitate optimization of levofloxacin dosing without the need to determine levofloxacin serum concentrations.

**Analysis Plan for Secondary Objectives**

*1.Determine which levofloxacin AUC/MIC provides the shortest time to sputum culture conversion in liquid medium.* Analyses will be performed as described under Primary analysis 1 above, but with liquid culture results substituted for solid medium culture results.

*2.Determine if baseline PZA susceptibility is associated with shorter time to sputum culture conversion after controlling for levofloxacin AUC.* Linear regression will be used to evaluate the relationship between PZA susceptibility at enrollment and log transferred time to sputum culture, adjusted for levofloxacin AUC and other predictors of time to sputum culture conversion.

*3. Describe the differences in safety and efficacy between assigned study dose groups.* The study is not powered to detect differences between the dosing groups in terms of efficacy or safety. This analysis will therefore be focused on description, rather than hypothesis testing. The median time to culture conversion with interquartile range will be presented by dosing group to describe differences in the primary efficacy endpoint. The number of grade 3, 4 and 5 adverse events (AEs) and the number of patients completing treatment will also be tabulated by dosing group to describe differences in safety and tolerability. Other secondary measures will also be summarized by randomized group to further describe any differences.

# Source Documents and Access to Source Data/Documents

Appropriate medical and research records will be maintained for this trial, in compliance with ICH E6 GCP, Section 4.9 and regulatory and institutional requirements for the protection of confidentiality of subjects.

The following individuals and groups will have access to study records:

 Members of the study team

 IRBs that review the study (including IRB members, staff, and legal counsel)

 Office of Human Research Protections

 Macleods Pharmaceuticals, Ltd.

 FDA

 DMID/NIAID/NIH

 Regulatory Agencies in Peru & South Africa

Authorized representatives of the sponsor(s), DMID, and regulatory agencies indicated above will be permitted to examine (and when required by applicable law, to copy) clinical records for the purposes of quality assurance reviews, audits and evaluation of the study safety and progress.

Documentation of source data is necessary for the reconstruction, evaluation, and validation of clinical findings, observations, and other activities during a clinical trial. Source documents are where participant information is first recorded. This includes medical records such as clinic progress notes, hospital charts, informed consent forms, and prescriptions, as well as laboratory reports and results of diagnostic testing, for which there may be additional primary source documentation within the relevant departments. Source documents also include any written and/or documented verbal communication between the clinical investigator and participant or clinical investigator and non-study health provider regarding the present and/or past medical history of the participant. Source documentation also includes tools developed for capturing study-specific data. (see MOP Section 6.4.1 for further details).

# Quality Control and Quality Assurance

The investigational site is responsible for conducting routine quality assurance (QA) and quality control (QC) activities to internally monitor study progress and protocol compliance. The Principal Investigator will provide direct access to all trial-related sites, source data/documents, and reports for the purpose of monitoring and auditing by the sponsor, and inspection by local and regulatory authorities. The Principal Investigator will ensure all study personnel are appropriately trained and applicable documentations are maintained on site.

Each site will prepare and follow a site-specific Quality Management Plan. This plan will ensure that data are accurately collected and that errors are promptly recognized and corrected. These plans are described in detail in the individual plans.

The clinical site monitors will verify that the clinical trial is conducted according to protocol, and that data are generated, documented (recorded), and reported in compliance with the protocol, Good Clinical Practice, and the applicable regulatory requirements.

Refer to protocol Section 10.1 for the Site Monitoring Plan and Section 15.1 for Data Management Responsibilities,

# Ethics/Protection of Human Subjects

The protection of human research subjects will be assured throughout the study execution and reporting.

## Ethical Standard

The investigators will ensure that this study is conducted in full conformity with the principles set forth in The Belmont Report: Ethical Principles and Guidelines for the Protection of Human Subjects of Research of the US National Commission for the Protection of Human Subjects of Biomedical and Behavioral Research (April 18, 1979) and codified in 45 CFR Part 46 and/or the ICH E6; 62 Federal Regulations 25691 (1997). In addition, the investigators will ensure that this study is conducted in full conformity with the current revision of the Declaration of Helsinki, or with the International Conference for Harmonizing Good Clinical Practice (ICH-GCP) regulations and guidelines, whichever affords the greater protection to the subject. In addition, this study will be conducted in accordance with South African and Peruvian standards.

## Institutional Review Board

Before they are placed into use, the study protocol and informed consent documents will be reviewed and approved by IRBs of all involved institutions (Boston University, CDC, Harvard University, MRC, University of Florida, University of Arkansas, University of California – San Francisco, South Africa and Peru) unless the institution decides that it is not engaged in human subjects research. Any amendments to the protocol or consent materials will be reviewed and approved by the IRBs engaged in human subjects research before they are placed into use. (See page 4 for a listing of IRBs)

## Informed Consent Process

Informed consent is a process that will be initiated prior to the individual’s agreeing to participate in the study and will continue throughout the individual’s study participation. Extensive discussion of risks and possible benefits of this therapy will be provided by study staff to potential subjects (and, with permission from potential subjects, their families). Potential subjects will receive counseling about study objectives and procedures, potential toxicities, and the informed consent process. Consent forms describing in detail the study interventions & products, study procedures and risks will be given to the subject and written documentation of informed consent will be required prior to starting intervention/administering study product. Consent forms will be IRB approved, will contain all the elements required by the ICH E6 Guideline for Good Clinical Practice and any additional elements required by local regulations. Forms for use in Peru will be written in Spanish; English versions will be available for IRB and sponsor review. The subject will be asked to read and review the document. Upon reviewing the document, the person obtaining consent will explain the research study to the subject and answer any questions that may arise. For subjects who speak and understand the language used in the consent document, but are unable to read or write, all of the information in the consent form will be communicated verbally, in the presence of an adult witness who is not a member of the study team; informed consent requires the signature or mark of the subject. The subject will sign or mark the informed consent document prior to any procedures being done specifically for the study. The subjects will have the opportunity to discuss the study with their surrogates or think about it prior to agreeing to participate. The subjects may withdraw consent at any time throughout the course of the trial. A copy of the signed and dated informed consent document will be given to the subjects for their records. The rights and welfare of the subjects will be protected by emphasizing, to subjects, that the quality of their medical care will not be adversely affected if they decline to participate in this study.

During the informed consent process, the subject will receive information about compensation for study participation. Specifically, subjects will not be paid for study participation, but their expenses and time will be reimbursed. Study subjects will receive vouchers for transportation to the health center/research facility, and monthly food baskets for the duration of time they are in the study. They will also receive meals while in the inpatient research facility for PK studies.

## Informed Consent/Assent Process (in Case of a Minor)

Not applicable. All study subjects will be ≥ 18 years of age.

## 14.5 Exclusion of Women, Minorities, and Children (Special Populations)

Children will be excluded from this study for three reasons. First, children under the age of 5 years have increased levofloxacin excretion, so that they will not achieve levofloxacin AUC similar to other study subjects in their dose category, increasing variability in the exposure variable and increasing sample size. Second, the smallest tablet form of levofloxacin available is 250 mg, and dosing persons under 40 kg is not practical with this formulation; therefore, all persons less than 40 kg have been excluded from the study because it is not be possible to preserve the blinding. Third, children with pulmonary TB often do not produce sputum; since following the decline of *M. tuberculosis* in sputum is a major outcome of the study, persons who do not produce sputum are also excluded.

Pregnant women will be excluded from this study because of the lack of evidence on the safety and tolerability of high-dose levofloxacin among fetuses.

## 14.6 Subject Confidentiality

This study will be performed in accordance with U.S., South African and Peruvian standards for protection of privacy of identifiable health information. All study records will be managed in a secure and confidential fashion. Study records will be maintained in locked cabinets, and computer records will be password protected. Access to study records will be restricted to specified team members. Methods for secure data handling are detailed below in Section 15.

The study monitor or other authorized representatives of the sponsor may inspect all documents and records required to be maintained by the Investigators, including but not limited to, case report forms and pharmacy records for the subjects in this study. The clinical study sites will permit access to such records.

## 14.7 Study Discontinuation

In the event that the study is discontinued (e.g. by the investigators and/or regulatory groups), subjects on study phase treatment will continue to receive TB treatment in accordance with national guidelines. Treatment will be offered free of charge, as guaranteed through the public health system in South Africa and Peru.

## Future Use of Stored Specimens

These types of specimens will be stored: *M. tuberculosis* isolates, serum and urine. Specimens collected for storage will be labeled with study identification number and collection date, and will not be labeled with participant name. Serum and urine will be collected at enrollment and at week 8 for storage as part of biomarker discovery and qualification studies. These sera will be stored at -70˚C and may be tested for biomarkers of tuberculosis treatment response.

*M. tuberculosis* isolates: baseline and treatment failure *M. tuberculosis* isolates will be shipped to the University of California, San Francisco laboratory and stored. Stored baseline and treatment failure *M. tuberculosis* isolates will be used for study-related drug susceptibility testing. In addition, stored *M. tuberculosis* isolates may be used for *M. tuberculosis* strain typing in order to assess whether persistently positive cultures (for an individual participant) are due to failure of TB treatment, laboratory contamination, or re-infection. Participant consent for stored specimens will be part of the study Informed Consent Form (ICF) which will be asked for during the Screening/Consenting visit.

# Data Handling and Record Keeping

Data handling and record keeping will be performed according to procedures that will be developed in a detailed Data Management Plan. The following types of forms will be used in this trial:

 Informed consent forms for enrollment, in English and translated into Spanish

 Eligibility screening forms

 Data collection forms that document enrollment, follow-up visits, adherence, and specimen collection

 Administrative forms that document participant deaths, termination from study drugs, and work up and treatment of AEs and treatment failure

A participant’s name will be collected only one time, and this information will be kept on a form that does not contain any test results, and that is filed separately from forms that do contain test results. Each participant will be assigned a unique study ID number. This number will be recorded on each data collection form and clinical specimen to facilitate linkage of data. The study ID number will be used on data collection forms; names and other obvious identifiers will not be used on data collection forms.

All data collection forms will be stored in locked files in a secure area. Access to study records and data files will be limited to study personnel, the NIH and its designees, the FDA, and local regulatory authorities.

All forms will be reviewed prior to data entry for accuracy, consistency, and completeness by designated study staff.

For banked specimens, the study ID number, date of collection, specimen volume, and freezer location will be recorded on the laboratory requisition forms and entered into the computer.

## 15.1 Data Management Responsibilities

The on-site principal investigator and the data manager will be responsible for the accuracy, completeness, and storage of source records and study data collection forms. The study team and data entry staff will review source documents and laboratory reports to ensure accuracy and completeness. The site staff will maintain logs to record dates of completed and upcoming clinic visits and specimen collections.

## 15.2 Data Capture Methods

All forms will be entered via Remote Data Capture (RDC) and verified according to procedures that will be developed in a Data Management Plan, SOPs, and Work Instructions.

## 15.3 Types of Data

Data for this study will include safety, laboratory (microbiology), and clinical data. Safety data will be recorded in the clinical database for this study.

## 15.4 Timing/Reports

The timing of reports will be detailed in the Data Management Plan. Briefly, the DSMB will review safety and tolerability of all doses three months after 40 patients have been enrolled and again three months after 60 patients have been enrolled; if any dose is found to exceed the safety or tolerability guidelines, the DSMB will recommend that enrollment in that study arm be terminated. Reports for the DSMB will be prepared for the DSMB according to a schedule determined at the first convened DSMB meeting. Data coding will occur at the time of data collection; ongoing logical data queries will be performed.

## 15.5 Study Records Retention

Within 2 years of completion of the study, identifiers excluding the study ID number will be deleted from computerized and paper data files. Study records will be maintained by the investigator for a minimum of 5 years following discontinuation of the study. The FDA and DMID will be notified prior to study records being destroyed.

## 15.6 Protocol Deviations

A protocol deviation is any noncompliance with the clinical trial protocol, Good Clinical Practice (GCP), or Manual of Procedures requirements. The noncompliance may be either on the part of the subject, the investigator, or the study site staff. As a result of deviations, corrective actions are to be developed by the site and implemented promptly.

It is the responsibility of the site to use continuous vigilance to identify and report deviations according to the guidelines of the IND sponsor. All deviations from the protocol must be addressed in study subject source documents. Protocol deviations must be sent to DMID and the local IRB in accordance with standard procedures.

# Publication Policy

Following completion of the study, the investigators plan to publish the results of this research in a scientific journal. The International Committee of Medical Journal Editors (ICMJE) member journals have adopted a trials-registration policy as a condition for publication. This trial will be registered in a public trials registry such as ClinicalTrials.gov before patient enrollment is initiated. It is anticipated that the results of this study will be submitted for publication in a peer-reviewed scientific journal. Authorship will be extended to the following individuals: study investigators, site PIs, and other individuals having major contribution to the study design, implementation, data analysis, and preparation of the written manuscript.

Prior to submission for presentation or publication, any materials derived wholly or in part from this study must be submitted to the study PI(s), site PI, and all co-authors for review.

# Literature References

**1. World Health Organization. Multidrug and extensively drug-resistant TB (M/CDR-TB): 2010 global report and surveillance response. 2010.**

**2. Jacobson KR, Tierney DB, Jeon CY, Mitnick CD, Murray MB. Treatment outcomes among patients with extensively drug-resistant tuberculosis: systematic review and meta-analysis. Clin Infect Dis 2010;51:6-14.**

**3. Dheda K, Shean K, Zumla A, et al. Early treatment outcomes and HIV status of patients with extensively drug-resistant tuberculosis in South Africa: a retrospective cohort study. Lancet 2010;375:1798-807.**

**4. Orenstein EW, Basu S, Shah NS, et al. Treatment outcomes among patients with multidrug-resistant tuberculosis: systematic review and meta-analysis. Lancet Infect Dis 2009;9:153-61.**

**5. Narita M, P A, M L, ES H, AE P, D A. Treatment experience of multidrug-resistant tuberculosis in Florida, 1994-1997. Chest 2001;120:343-8.**

**6. Park M, AL D, al e. Outcome of MDR-TB patients, 1983-1993: prolonged survival with appropriate therapy. American Journal of Respiratory and Critical Care Medicine 1996;153:317-24.**

**7. Department of Health and Human Services CfDC. Emergence of Mycobacterium tuberculosis with extensive resistance to second-line drugs--worldwide, 2000-2004. MMWR Morb Mortal Wkly Rep 2006;55:301-5.**

**8. Holtz T, M S, S K, et al. Time to Sputum Culture Conversion in Multidrug-Resistant Tuberculosis: Predictors and Relationship to Treatment Outcome. Annals of Internal Medicine 2006;144:650-9.**

**9. Park S, WC L, DH L, CD M, L H, KJ S. Self-administered, standardized regimens for multidrug-resistant tuberculosis in South Korea. Int J Tuberc Lung Dis 2004;8:361-8.**

**10. Chan E, V L, MJ S, et al. Treatment and Outcome Analysis of 205 Patients with Multidrug-resistant Tuberculosis. American Journal of Respiratory and Critical Care Medicine 2004;169:1103-9.**

**11. Leimane V, Riekstina V, Holtz TH, et al. Clinical outcome of individualised treatment of multidrug-resistant tuberculosis in Latvia: a retrospective cohort study. Lancet 2005;365:318-26.**

**12. Kim H, P H, SJ K, WJ L, EG L. Ambulatory treatment of multidrug-resistant pulmonary tuberculosis patients at a chest clinic. Int J Tuberc Lung Dis 2001;5:1129-36.**

**13. Tahaoğlu K, T T, T S, et al. The Treatment of Multidrug-Resistant Tuberculosis in Turkey. New England Journal of Medicine 2001;345:170-4.**

**14. Mitnick C, J B, E P, et al. Community-Based Therapy for Multidrug-Resistant Tuberculosis in Lima, Peru. New England Journal of Medicine 2003;348:119-28.**

**15. Yew WW, Chan CK, Chau CH, et al. Outcomes of patients with multidrug-resistant pulmonary tuberculosis treated with ofloxacin/levofloxacin-containing regimens. Chest 2000;117:744-51.**

**16. Goble M, Iseman M, al e. Treatment of 171 patients with pulmonary tuberculosis resistant to isoniazid and rifampin. N Engl J Med 1993;328:527-32.**

**17. World Health Organization. Global tuberculosis control - epidemiology, strategy, financing. WHO Report 2009. . 2009.**

**18. World Health Organization. Molecular line probe assays for rapid screening of patients at risk of multidrug-resistant tuberculosis (MDR-TB). Policy statement. . 2008.**

**19. Chan ED, Laurel V, Strand MJ, et al. Treatment and outcome analysis of 205 patients with multidrug-resistant tuberculosis. Am J Respir Crit Care Med 2004;169:1103-9.**

**20. Tahaoglu K, Torun T, Sevim T, et al. The treatment of multidrug-resistant tuberculosis in Turkey. N Engl J Med 2001;345:170-4.**

**21. Yew WW, Kwan SY, Ma WK, Khin MA, Chau PY. In-vitro activity of ofloxacin against Mycobacterium tuberculosis and its clinical efficacy in multiply resistant pulmonary tuberculosis. J Antimicrob Chemother 1990;26:227-36.**

**22. Sirgel FA, Botha FJ, Parkin DP, et al. The early bactericidal activity of ciprofloxacin in patients with pulmonary tuberculosis. Am J Respir Crit Care Med 1997;156:901-5.**

**23. Tsukamura M, Nakamura E, Yoshii S, Amano H. Therapeutic effect of a new antibacterial substance ofloxacin (DL8280) on pulmonary tuberculosis. Am Rev Respir Dis 1985;131:352-6.**

**24. Park S, WC L, DH L, CD M, L H, KJ S. Self-administered, standardized regimens for multidrug-resistant tuberculosis in South Korea. Int J Tuberc Lung Dis 2004;8:361-8.**

**25. Frieden TR, Sherman LF, Maw KL, et al. A multi-institutional outbreak of highly drug-resistant tuberculosis: epidemiology and clinical outcomes. JAMA 1996;276:1229-35.**

**26. Ginsburg AS, Grosset JH, Bishai WR. Fluoroquinolones, tuberculosis, and resistance. Lancet Infect Dis 2003;3:432-42.**

**27. Ji B, Lounis N, Maslo C, Truffot-Pernot C, Bonnafous P, Grosset J. In vitro and in vivo activities of moxifloxacin and clinafloxacin against Mycobacterium tuberculosis. Antimicrob Agents Chemother 1998;42:2066-9.**

**28. Ji B, Lounis N, Truffot-Pernot C, Grosset J. In Vitro and In Vivo Activities of Levofloxacin against Mycobacterium tuberculosis. Antimicrob Agents Chemother 1995:1341-4.**

**29. Iseman M. Treatment of multidrug-resistant tuberculosis. N Engl J Med 1993;329:784-91.**

**30. Marra F, Marra CA, Moadebi S, et al. Levofloxacin treatment of active tuberculosis and the risk of adverse events. Chest 2005;128:1406-13.**

**31. Yew WW, Chan CK, Leung CC, et al. Comparative roles of levofloxacin and ofloxacin in the treatment of multidrug-resistant tuberculosis: preliminary results of a retrospective study from Hong Kong. Chest 2003;124:1476-81.**

**32. Mitnick CD, Shin SS, Seung KJ, et al. Comprehensive treatment of extensively drug-resistant tuberculosis. N Engl J Med 2008;359:563-74.**

**33. Aubry A, Veziris N, Cambau E, Truffot-Pernot C, Jarlier V, Fisher LM. Novel gyrase mutations in quinolone-resistant and -hypersusceptible clinical isolates of Mycobacterium tuberculosis: functional analysis of mutant enzymes. Antimicrob Agents Chemother 2006;50:104-12.**

**34. Kam KM, Yip CW, Cheung TL, Tang HS, Leung OC, Chan MY. Stepwise decrease in moxifloxacin susceptibility amongst clinical isolates of multidrug-resistant Mycobacterium tuberculosis: correlation with ofloxacin susceptibility. Microb Drug Resist 2006;12:7-11.**

**35. Cox HS, Sibilia K, Feuerriegel S, et al. Emergence of extensive drug resistance during treatment for multidrug-resistant tuberculosis. N Engl J Med 2008;359:2398-400.**

**36. Diacon AH, Pym A, Grobusch M, et al. The diarylquinoline TMC207 for multidrug-resistant tuberculosis. N Engl J Med 2009;360:2397-405.**

**37. Diacon A. Oral Presentation at IUATLD Meeting, Berlin. 2010.**

**37a. Gler MT, Skripconoka V, Sanchez-Garavito E, et al. Delamanid for multidrug-resistant pulmonary tuberculosis. New England Journal of Medicine 2012;366:2151-60**

**38. van den Boogaard J, Kibiki GS, Kisanga ER, Boeree MJ, Aarnoutse RE. New drugs against tuberculosis: problems, progress, and evaluation of agents in clinical development. Antimicrob Agents Chemother 2009;53:849-62.**

**39. Lenaerts AJ, Bitting C, Woolhiser L, et al. Evaluation of a 2-pyridone, KRQ-10018, against Mycobacterium tuberculosis in vitro and in vivo. Antimicrob Agents Chemother 2008;52:1513-5.**

**40. Falzon D, Jaramillo E, Schunemann HJ, et al. WHO guidelines for the programmatic management of drug-resistant tuberculosis: 2011 update. Eur Respir J 2011.**

**41. Demolis JL, Kubitza D, Tenneze L, Funck-Brentano C. Effect of a single oral dose of moxifloxacin (400 mg and 800 mg) on ventricular repolarization in healthy subjects. Clin Pharmacol Ther 2000;68:658-66.**

**42. Noel GJ, Goodman DB, Chien S, Solanki B, Padmanabhan M, Natarajan J. Measuring the effects of supratherapeutic doses of levofloxacin on healthy volunteers using four methods of QT correction and periodic and continuous ECG recordings. J Clin Pharmacol 2004;44:464-73.**

**43. Van Deun A, Maug AK, Salim MA, et al. Short, highly effective, and inexpensive standardized treatment of multidrug-resistant tuberculosis. Am J Respir Crit Care Med 2010;182:684-92.**

**44. Blumberg HM, Burman WJ, Chaisson RE, et al. American Thoracic Society/Centers for Disease Control and Prevention/Infectious Diseases Society of America: treatment of tuberculosis. Am J Respir Crit Care Med 2003;167:603-62.**

**45. World Health Organization. Treatment of tuberculosis guidelines. 4th edition. . 2010.**

**46. Johnson JL, Hadad DJ, Boom WH, et al. Early and extended early bactericidal activity of levofloxacin, gatifloxacin and moxifloxacin in pulmonary tuberculosis. Int J Tuberc Lung Dis 2006;10:605-12.**

**47. Shandil RK, Jayaram R, Kaur P, et al. Moxifloxacin, ofloxacin, sparfloxacin, and ciprofloxacin against Mycobacterium tuberculosis: evaluation of in vitro and pharmacodynamic indices that best predict in vivo efficacy. Antimicrob Agents Chemother 2007;51:576-82.**

**48. Richeldi L, Covi M, Ferrara G, et al. Clinical use of Levofloxacin in the long-term treatment of drug resistant tuberculosis. Monaldi Arch Chest Dis 2002;57:39-43.**

**49. Thwaites GE, Bhavnani SM, Chau TT, et al. Randomized pharmacokinetic and pharmacodynamic comparison of fluoroquinolones for tuberculous meningitis. Antimicrob Agents Chemother 2011;55:3244-53.**

**50. Farrar J. personal communication. 2011.**

**51. Noel GJ, Blumer JL, Pichichero ME, et al. A randomized comparative study of levofloxacin versus amoxicillin/clavulanate for treatment of infants and young children with recurrent or persistent acute otitis media. The Pediatric infectious disease journal 2008;27:483-9.**

**52. Noel GJ, Bradley JS, Kauffman RE, et al. Comparative safety profile of levofloxacin in 2523 children with a focus on four specific musculoskeletal disorders. The Pediatric infectious disease journal 2007;26:879-91.**

**53. Grimaldo ER, Tupasi TE, Rivera AB, et al. Increased resistance to ciprofloxacin and ofloxacin in multidrug-resistant mycobacterium tuberculosis isolates from patients seen at a tertiary hospital in the Philippines. Int J Tuberc Lung Dis 2001;5:546-50.**

**54. Devasia RA, Blackman A, Gebretsadik T, et al. Fluoroquinolone resistance in Mycobacterium tuberculosis: the effect of duration and timing of fluoroquinolone exposure. Am J Respir Crit Care Med 2009;180:365-70.**

**55. Long R, Chong H, Hoeppner V, et al. Empirical treatment of community-acquired pneumonia and the development of fluoroquinolone-resistant tuberculosis. Clin Infect Dis 2009;48:1354-60.**

**56. Shin SS, Keshavjee S, Gelmanova IY, et al. Development of extensively drug-resistant tuberculosis during multidrug-resistant tuberculosis treatment. Am J Respir Crit Care Med 2010;182:426-32.**

**57. Hillemann D, Rusch-Gerdes S, Richter E. Feasibility of the GenoType MTBDRsl assay for fluoroquinolone, amikacin-capreomycin, and ethambutol resistance testing of Mycobacterium tuberculosis strains and clinical specimens. J Clin Microbiol 2009;47:1767-72.**

**58. Von Groll A, Martin A, Jureen P, et al. Fluoroquinolone resistance in Mycobacterium tuberculosis and mutations in gyrA and gyrB. Antimicrob Agents Chemother 2009;53:4498-500.**

**59. Gumbo T, Louie A, Deziel MR, Drusano GL. Pharmacodynamic evidence that ciprofloxacin failure against tuberculosis is not due to poor microbial kill but to rapid emergence of resistance. Antimicrob Agents Chemother 2005;49:3178-81.**

**60. Drusano GL, Preston SL, Fowler C, Corrado M, Weisinger B, Kahn J. Relationship between fluoroquinolone area under the curve: minimum inhibitory concentration ratio and the probability of eradication of the infecting pathogen, in patients with nosocomial pneumonia. J Infect Dis 2004;189:1590-7.**

**61. Holtz T, M S, S K, et al. Time to Sputum Culture Conversion in Multidrug-Resistant Tuberculosis: Predictors and Relationship to Treatment Outcome. Annals of Internal Medicine 2006;144:650-9.**

**62. Mitchison D. Assessment of new sterilizing drugs for treating pulmonary tuberculosis by culture at 2 months. Am Rev Respir Dis 2003;147:1062-3.**

**63. Weiner M, Prihoda TJ, Burman W, et al. Evaluation of time to detection of Mycobacterium tuberculosis in broth culture as a determinant for end points in treatment trials. J Clin Microbiol 2010;48:4370-6.**

**64. Davies G, R B, SH K, LJ A. Use of Nonlinear Mixed-Effects Analysis for Improved Precision of Early Pharmacodynamic Measures in Tuberculosis Treatment. Antimicrobial Agents and Chemotherapy 2006;50:3154-6.**

**65. Davies G, SH K, LJ A. Optimal sampling strategies for early pharmacodynamic measures in tuberculosis. Journal of Antimicrobial Chemotherapy 2006;58:594-600.**

**66. Esptein M, NW S, AL D, S B, WN R, B H. Time to detection of Mycobacterium tuberculosis in sputum culture correlates with outcome in patients receiving treatment for pulmonary tuberculosis. Chest 1998;113:379-86.**

**67. Joloba M, JL J, al e. Quantitative sputum bacillary load during rifampin-containing short course chemotherapy in human immunodeficiency virus-infected and non- infected adults with pulmonary tuberculosis. Int J Tuberc Lung Dis 2000;4:528-36.**

**68. Perrin F, MC L, TD M, SH G. Biomarkers of treatment response in clinical trials of novel antituberculosis agents. Lancet Infectious Diseases 2007;7:481-90.**

**69. Hesseling AC, Walzl G, Enarson DA, et al. Baseline sputum time to detect ion predicts month two culture conversion and relapse in non-HIV-infected patients. Int J Tuberc Lung Dis 2010;14:560-70.**

**70. Wispelwey B. Clinical implications of pharmacokinetics and pharmacodynamics of fluoroquinolones. Clin Infect Dis 2005;41 Suppl 2:S127-35.**

**71. Kohanski MA, Dwyer DJ, Hayete B, Lawrence CA, Collins JJ. A common mechanism of cellular death induced by bactericidal antibiotics. Cell 2007;130:797-810.**

**72. Peloquin CA, Hadad DJ, Molino LP, et al. Population pharmacokinetics of levofloxacin, gatifloxacin, and moxifloxacin in adults with pulmonary tuberculosis. Antimicrob Agents Chemother 2008;52:852-7.**

**73. Preston SL, Drusano GL, Berman AL, et al. Levofloxacin population pharmacokinetics and creation of a demographic model for prediction of individual drug clearance in patients with serious community-acquired infection. Antimicrob Agents Chemother 1998;42:1098-104.**

**74. World Health Organization. Guidelines for the programmatic management of drug-resistant tuberculosis. Emergency update 2008. 2008.**

**75. Thwaites GE, Bhavnani SM, Thi Hong Chau T, et al. A randomised pharmacokinetic and pharmacodynamic comparison of fluoroquinolones for tuberculous meningitis. Antimicrob Agents Chemother 2011.**

**76. Heifets LB, Iseman MD, Lindholm-Levy PJ. Ethambutol MICs and MBCs for Mycobacterium avium complex and Mycobacterium tuberculosis. Antimicrob Agents Chemother 1986;30:927-32.**

**77. Hsieh FY, Bloch DA, Larsen MD. A simple method of sample size calculation for linear and logistic regression. Stat Med 1998;17:1623-34.**

**78. Cockroft DW, Gault MH. Prediction of creatinine clearance from serum creatinine. Nephron 1976;16:31-41.**

# APPENDIX A: SCHEDULE OF EVENTS*

| Event | Screening/ Consenting Visit | Randomization/ Baseline | Wk  1 | Wk  2 | Wk  3 | Wk  4 | Wk  5 | Wk  6 | Wk  7 | Wk  8 | Wk 10 | Wk 12 | Wk 16 | Wk 20 | Wk 24 | Wk 28** |
| --- | --- | --- | --- | --- | --- | --- | --- | --- | --- | --- | --- | --- | --- | --- | --- | --- |
| ****Clinical**** |  |  |  |  |  |  |  |  |  |  |  |  |  |  |  |  |
| Informed consent^‡^ | X |  |  |  |  |  |  |  |  |  |  |  |  |  |  |  |
| History, demographics | X | X |  |  |  |  |  |  |  |  |  |  |  |  |  |  |
| Targeted symptom assessment | X | X | X | X | X | X | X | X | X | X | X | X | X | X | X | X |
| Focused clinical assessment | X | X | X | X | X | X | X | X | X | X | X | X | X | X | X | X |
| Karnofsky Score | X | X |  |  |  |  |  |  |  |  |  |  |  |  |  |  |
| Review of previous/interval TB treatment | X | X |  |  |  |  |  |  |  |  |  |  |  |  |  |  |
| Height | X |  |  |  |  |  |  |  |  |  |  |  |  |  |  |  |
| Weight | X | X | X | X | X | X | X | X | X | X | X | X | X | X | X | X |
| Eligibility | X | X |  |  |  |  |  |  |  |  |  |  |  |  |  |  |
| Concomitant medications | X | X | X | X | X | X | X | X | X | X | X | X | X | X | X | X |
| Adverse events |  | X | X | X | X | X | X | X | X | X | X | X | X | X | X | X |
| DOT Log Review |  |  | X | X | X | X | X | X | X | X | X | X | X | X | X |  |
| ****Laboratory**** |  |  |  |  |  |  |  |  |  |  |  |  |  |  |  |  |
| Sputum smear | X | X |  |  |  |  |  |  |  |  |  |  |  |  |  |  |
| Sputum culture | X | X |  | X |  | X |  | X |  | X | X | X | X | X | X | X |
| DNA-based susceptibility | X |  |  |  |  |  |  |  |  |  |  |  |  |  |  |  |
| Chemistry and hematology | X | X |  | X |  | X |  | X |  | X |  | X | X | X | X | X |
| Serum/urine for bank |  | X |  |  |  |  |  |  |  | X |  |  |  |  |  |  |
| Pregnancy Test | X | X |  |  |  | X |  |  |  | X |  | X | X | X | X |  |
| HIV serology / history | X |  |  |  |  |  |  |  |  |  |  |  |  |  |  |  |
| Chest X-ray | X |  |  |  |  |  |  |  |  |  |  |  |  |  |  |  |
| EKG | X | X |  |  |  | X |  |  |  | X |  | X | X | X | X |  |
| PK evaluation^+^ |  |  |  | X |  |  |  |  |  |  |  |  |  |  |  |  |

*****weekly visits to occur on the day 6,7,or 8 days after the expected date for the previous visit

** or 4 weeks after completion of medication

^+^at any time between week 2 and week 4

^‡^ Includes futures use permission for serum/urine bio-repository

# APPENDIX B: KARNOFSKY SCALE

| Able to carry on normal activity | 100 | Normal; no complaints; no evidence of disease |
| --- | --- | --- |
|  | 90 | Able to carry on normal activity; minor signs or symptoms of disease |
|  | 80 | Normal activity with effort; some signs or symptoms of disease |
| Unable to work; able to live at home and care for most personal needs; a varying amount of assistance is needed | 70 | Cares for self; unable to carry on normal activity or to do active work |
|  | 60 | Requires occasional assistance but is able to care for most of his/her needs |
|  | 50 | Requires considerable assistance and frequent medical care |
| Unable to care for self; requires equivalent of institutional or hospital care; disease may be progressing rapidly | 40 | Disabled; requires special care and assistance |
|  | 30 | Severely disabled; hospitalization is indicated although death is not imminent |
|  | 20 | Very sick; hospitalization necessary; active supportive treatment is necessary |
|  | 10 | Moribund; fatal process progressing rapidly |
|  | 0 | Dead |

# APPENDIX C: TOXICITY MANAGEMENT ALGORITHM

| Sign/symptom | Other Potential Causal Agent | Management Strategy  Grade 1 | Management Strategy  Grade 2 | Management Strategy Grade 3 |
| --- | --- | --- | --- | --- |
| Dizziness, restlessness, anxiety, insomnia or other sleep disorder | Efavirenz, Cycloserine, Terazadone | Stop Efavirenz (or reduce dose); If not on Efavirenz, stop Cycloserine/ Terazadone; if symptom does not subside in 3 days, stop levo; if symptom does not subside in 3 days, restart levo and look for other causes | Same as Grade 1 | Same as Grade 1 |
| Headache | Cycloserine, Terazadone | Stop Cycloserine/ Terazadone; attribute to levo if symptom does not subside in 3 days; if symptom does not subside in 3 days, restart levo and look for other causes; if symptom directly follows administration of meds, give levo in afternoon and keep rest of regimen in morning | Same as Grade 1 | Same as Grade 1 |
| Paranoia, depression, hallucinations, psychosis | Cycloserine, Terazadone | Stop Cycloserine/ Terazadone; attribute to levo if symptom does not subside in 3 days | Same as Grade 1 | Same as Grade 1 |
| Abdominal pain | Ethionamide, PAS, PZA | If symptom directly follows administration of meds, give levo in afternoon and keep rest of regimen in morning; if not, stop Ethionamide, then PAS, then PZA and then levo | Same as Grade 1 | Same as Grade 1 |
| Nausea, vomiting, diarrhea | Ethionamide, PAS, AZT | If symptom directly follows administration of meds, give levo in afternoon and keep rest of regimen in morning; if not, stop Ethionamide, then PAS and then levo | Evaluate for *C. difficile* colitis; manage as in Grade 1 | Evaluate for *C. difficile*colitis; manage as in Grade 1 |
| Peripheral neuropathy, paresthesias, weakness, tingling, numbness | INH, stavudine, didanosine, Linezolid | Stop stavudine or didanosine if patient taking these agents; then stop Linezolid if patient is on Linezolid; if not, and patient is on INH, then add or increase B_6_; if symptom does not resolve following this, then stop INH; attribute to levo if patient not on Linezolid and symptom does not subside in 7 days after discontinuation of INH | Same as Grade 1 | Same as Grade 1 |
| Hypersensitivity (rash, itching, urticaria) | Cycloserine, Terizidone, Ethionamide, PAS, INH, PZA, Nevirapine, abacavir,; less commonly efavirenz or etravirine | Give Benadryl and continue all meds | If on nevirapine or abacavir, stop these first. If not, stop INH first, then PZA, then Ethionamide, then PAS, then Cycloserine/Terizidone | Stop all meds and reintroduce sequentially, starting with levo after symptoms abate |
| Arthralgia, myalgia | PZA | Obtain uric acid level and hold PZA. If symptom does not resolve, treat with analgesics and continue meds | Same as Grade 1 | Obtain uric acid level and hold PZA. If symptom does not resolve, hold FQ for 3 days and determine if symptoms improve. |
| Elevated LFTs | Ethionamide, PAS, INH, PZA, nevirapine | Stop INH first, then PZA, then Ethionamide, then PAS, then levo | Stop all meds and reintroduce sequentially, starting with levo after LFTs normalize | Stop all meds and reintroduce sequentially, starting with levo after LFTs normalize |
| Elevated or decreased Blood Glucose |  | Obtain fasting Glucose; observe | Stop levo | Stop levo |
| Decreased WBC |  | Observe | Stop levo for 3 days and re-evaluate | Stop levo for 3 days and re-evaluate |
| Decreased Hemoglobin |  | Observe | Stop levo for 3 days and re-evaluate | Stop levo for 3 days and re-evaluate |

(all grade 4 toxicities will be study endpoints, whether judged related to levo or not)

1. The only birth control methods that work well enough to be safe while you are on this study are oral contraceptives (the pill), intrauterine devices (IUDs), contraceptive implants under the skin, contraceptive rings or patches or injections, diaphragms with spermicide and condoms with foam. You should not participate in this study if you are a woman who has sex with men and cannot use one of these birth control methods. [↑](#footnote-ref-2)
