# Supplementary material for: An optimized background regimen design to evaluate the contribution of levofloxacin to multidrug-resistant tuberculosis treatment regimens: study protocol for a randomized controlled trial
Source: Trials. 2017 Nov 25;18:563. doi: 10.1186/s13063-017-2292-x (PMC5702225; doi:10.1186/s13063-017-2292-x)
Supplement: Supplementary file 3 — Ethical approval reference numbers. This document contains a list of the specific names and reference numbers for all ethical bodies that approved the study in the various participating and recruiting centers involved. (PDF 207 kb) [file 13063_2017_2292_MOESM3_ESM.pdf]

IRB Institutions that approved Opti-Q protocol

IRB Institution	FWA number	Registration number
**Boston University Medical Campus**	00005790	IORG0000222
**Harvard Medical School**	00007071	IORG0000183
**University of Florida**	00005790	IORG0000203
**University of Arkansas**	00001119	IORG0000345
**University of Stellenbosch Human Research Ethics Committee**	00005239/00005240	IORG0004423
**ERB Universidad Peruana Cayetano Heredia**	00000525	IORG0000671
**Comite Instrucional de Etica en Investigacion del Hospital Nacional Hipolito Unanue**	00015265	IORG0005793
