# Supplementary material for: An optimized background regimen design to evaluate the contribution of levofloxacin to multidrug-resistant tuberculosis treatment regimens: study protocol for a randomized controlled trial
Source: Trials. 2017 Nov 25;18:563. doi: 10.1186/s13063-017-2292-x (PMC5702225; doi:10.1186/s13063-017-2292-x)
Supplement: Supplementary file 4 — OPTIQ Informed Consent Form. This document is the approved consent form used to enroll eligible patients into the OPTI-Q study. (PDF 260 kb) [file 13063_2017_2292_MOESM4_ESM.pdf]

RESEARCH CONSENT FORM (v6.0)

Patient Consent for Participation in the Research Study
For Persons 18 years of Age and Older

Title of Project: Prospective, Randomized, Blinded Phase 2 Pharmacokinetic/Pharmacodynamic Study of the Efficacy and Tolerability of Levofloxacin in Combination with Optimized Background Regimen (OBR) for the treatment of MDR-TB (DMID # 13-0057)

Principal Investigator: Dr. Charles Horsburgh

Background

You are being asked to be in this research study because you have tuberculosis (TB) that is resistant to the drugs normally used to treat TB. Boston University Medical Center and the United States of America Centers for Disease Control and Prevention (CDC), and the National Institutes for Health (NIH) are working together on this study to improve the current treatment of drug resistant TB.

Your doctor is also an investigator of this research study. As an investigator, your doctor is interested both in your clinical welfare and in the conduct of this study. Before entering this study or at any time during this research, you may want to ask for a second opinion about your care from another doctor who is not an investigator in this study. You do not have to participate in any research study offered by your doctor. Refusal to participate will involve no penalty or loss of benefits to which you are entitled. You may discontinue participation at any time without penalty or loss of these benefits.

Purpose

People who have drug resistant TB are treated with different drugs (for example antibiotics, vitamins) that have been to be shown to be effective. It may take four or more different antibiotics to treat the TB. One of most effective of these antibiotic drugs is levofloxacin. The most frequently prescribed dose of levofloxacin is 750 mg given daily as part of combination of several drugs to treat drug resistant TB. We want to find the dose of levofloxacin that works the best in treatment of drug resistant TB. We also want to see if any side effects are caused by higher doses of levofloxacin.

In this study, we will use higher doses of levofloxacin given every day as part of TB treatment. The amount of levofloxacin that we use depends on your weight. Levofloxacin is "experimental" in this research study because giving higher doses has not been investigated yet, although some studies suggest that higher doses are likely to work better. The four doses we will be prescribing are the equivalent for your weight of 750mg, 1000mg, 1250mg, and 1500mg daily.

BU/BMC Institutional Review Board
IRB NUMBER: H-32150
IRB APPROVAL DATE: 04/20/2016

RESEARCH CONSENT FORM (v6.0)

Patient Consent for Participation in the Research Study
For Persons 18 years of Age and Older

Title of Project: Prospective, Randomized, Blinded Phase 2 Pharmacokinetic/Pharmacodynamic Study of the Efficacy and Tolerability of Levofloxacin in Combination with Optimized Background Regimen (OBR) for the treatment of MDR-TB (DMID # 13-0057)

Principal Investigator: Dr. Charles Horsburgh

All of the other medicines that will be given to you for treatment of TB along with the levofloxacin are recommended by the World Health Organization (WHO) and *(Your Country's)* TB program and drug regulatory board for the treatment of TB. They will be prescribed by your TB doctor in the same way whether or not you decide to take part in this study.

<u>What Happens In This Research Study</u>

You will be one of approximately 240 subjects to be asked to participate at this location. A total of approximately 240 subjects at all institutions will be asked to participate in this study.

The research will take place at the following location(s): Cape Town, South Africa and Lima, Peru.

How many people will take part in this study?

About 120 people 18 years of age or older will be enrolled in this part of the study.

How long will I be in the study?

You will be in the study for the first 6 months (24 weeks) of the time you are receiving treatment for TB plus follow-up visit four weeks later (making a total of 28 weeks). Complete treatment of your TB will last for 18 to 24 months. You will see your local doctor to complete treatment after the study is over. The length of your treatment will depend on how bad your TB is to start with and how quickly you respond to the TB medicines.
You will be discontinued from the study if:

- Your first sputum tests for TB are negative (do not grow TB) or
- It is discovered that your TB can be treated with isoniazid or rifampin
- It is discovered that levofloxacin will not kill the type of TB you have
- If you choose at any time not to continue

BU/BMC Institutional Review Board
IRB NUMBER: H-32150
IRB APPROVAL DATE: 04/20/2016

RESEARCH CONSENT FORM (v6.0)

Patient Consent for Participation in the Research Study
For Persons 18 years of Age and Older

Title of Project: Prospective, Randomized, Blinded Phase 2 Pharmacokinetic/Pharmacodynamic Study of the Efficacy and Tolerability of Levofloxacin in Combination with Optimized Background Regimen (OBR) for the treatment of MDR-TB (DMID # 13-0057)

Principal Investigator: Dr. Charles Horsburgh

What is involved in the study?

In the treatment part of the study:

If you agree to be in this study, you will be assigned to 1 of 4 study treatments for the first 24 weeks of TB treatment. Your treatment will be chosen by chance. You have an equal chance of being assigned to any of the 4 TB treatments. You will get either:

750mg of Levofloxacin equivalent to your body weight plus the best combination of drugs for you (optimized Background Regimen [OBR]);

OR

1000mg of Levofloxacin equivalent to your body weight plus the best combination of drugs for you (OBR);

OR

1250mg of Levofloxacin equivalent to your body weight plus the best combination of drugs for you (OBR);

OR

1500mg of Levofloxacin equivalent to your body weight plus the best combination of drugs for you (OBR);

The Levoflaxin medication and OBR must be taken **every day**, including weekends and holidays. A healthcare worker will give you all doses of the medicines, Monday through Friday. The study team will work with you to develop a plan for taking the medicines on weekends.

Other medicines during study treatment:

In this study, you cannot take certain medicines during your participation in the study. You should tell the study nurse/coordinator or doctor about all the medicines you are taking. You should talk with them before you start any new medicine during your TB treatment. This includes over-the-counter medicines such as antacids, Tylenol, vitamins, or any other medicines and herbal or natural medicinal products. You must take your TB medicines at least 4 hours before or 8 hours after taking antacids, medicine for diarrhea, or medicine with iron or zinc because these other medicines may keep the TB medicines from working.

BU/BMC Institutional Review Board
IRB NUMBER: H-32150
IRB APPROVAL DATE: 04/20/2016

RESEARCH CONSENT FORM (v6.0)

Patient Consent for Participation in the Research Study
For Persons 18 years of Age and Older

Title of Project: Prospective, Randomized, Blinded Phase 2 Pharmacokinetic/Pharmacodynamic Study of the Efficacy and Tolerability of Levofloxacin in Combination with Optimized Background Regimen (OBR) for the treatment of MDR-TB (DMID # 13-0057)

Principal Investigator: Dr. Charles Horsburgh

You will have a screening visit to see if you are eligible for the study followed by a baseline visit. Below is a list of what will be done at each of the visits.

Screening / Consenting visit:

- Informed consent obtained
- Medical history and physical examination
- Chest x-ray (unless done within the last 14 days)
- Obtain four teaspoons of blood to perform the following tests:
  - A test for HIV (Human Immunodeficiency Virus, the virus that causes AIDS). This will not be done if you are HIV positive or if you've had a negative HIV test within the past 3 months and can provide documentation the test was negative. This is a recommended test for everyone with TB and is required for this study.
  - CD4 count if HIV infected (and within 6 mos)
  - Blood tests to check your liver, kidneys, and blood count (unless done within the last 14 days)
- Electrocardiogram (EKG or ECG)
- Urine or blood test for pregnancy, if you are a woman who can get pregnant
- Sputum that is tested for TB and for drug resistance by cultures and by rapid testing.

Randomization / Baseline visit

Before starting the study medicines, you will have the following done:

- Symptom assessment and physical examination
- Four (4) teaspoons of blood collected to perform the following tests:
  - Blood tests to check your liver, kidneys and blood count
  - Blood to be frozen and stored (if you agree to storage and use of your samples for future research). This blood will not have any information on it that directly identifies you. The blood may be tested if you have a reaction to the study medicines. It may also be used for future studies to improve our understanding of TB and how to best treat TB. No human genetic testing will be done.

BU/BMC Institutional Review Board
IRB NUMBER: H-32150
IRB APPROVAL DATE: 04/20/2016

- Urine collected (two (2) teaspoons) to be frozen and stored for future research about TB treatment (if you give permission). This urine will not have any information on it that directly identifies you. No human genetic testing will be done.
- Electrocardiogram (EKG or ECG)
- Urine or blood test for pregnancy, if you are a woman who can get pregnant. The test must be negative before you can start this study.
- Sputum that is tested for TB

During the study:

During the 24 weeks that you are taking the study treatment, you will meet with a study nurse/ coordinator or doctor. During these visits, there are study procedures that will be done. These visits will happen ONCE A WEEK for the first 8 weeks (i.e. weeks 1, 2, 3, 4, 5, 6, 7, 8), and again at week 10, and then monthly at weeks 12, 16, 20, 24 and 28 (or 4 weeks after you stop study medicines). The visits will take about 30 minutes each and are explained below:

Study Visits:

Weeks 1, 3, 5, 7
- Vital signs and weight
- Review of symptoms, other medicines you are taking including the Directly Observed Treatment (DOT) log, and a physical examination
- Adverse event assessment

Weeks 2, 6, 10, 28 (or 4 weeks after you stop study medicines)
- Vital signs and weight
- Review of symptoms, other medicines you are taking including DOT log, and a physical examination
- Adverse event assessment
- Sputum collection
- Blood tests to check your liver, kidneys, and blood count (approximately 2 teaspoons of blood)

Between Weeks 2-4 (one 24 –hour period in this interval)

- Dietary history
- Seven (7) blood samples will be collected over a 24-hour period that will help us to study how your body processes the levofloxacin that you take. (Each sample will be approximately 2 teaspoons of blood). You will have to fast, meaning go without food or water for 8 hours, before coming for this hospital visit in the morning. You will then be given the study drug with water and will not be able to eat for 2 more hours. This visit may require you to stay overnight in the hospital. This part of the study will help us understand how to prescribe the safe and effective amounts of levofloxacin.

Week 8

- Vital signs
- Review of symptoms, other medicines you are taking including DOT log, and a physical examination
- Adverse event assessment
- Sputum collection
- Blood tests to check your liver, kidneys, blood count and blood sugar
- Urine or blood pregnancy test if you are a woman who can get pregnant
- Electrocardiogram (EKG)
- Two (2) teaspoons of blood to be frozen and stored (if you give permission). This blood will not have any information on it that directly identifies you. The blood may be tested if you have a reaction to the study medicines. It may also be used for future studies to improve our understanding of TB and how to best treat TB. No human genetic testing will be done.
- Urine (two (2) teaspoons) to be frozen and stored for future research if you give permission. This urine will not have any information on it that directly identifies you. No human genetic testing will be done.

Weeks 4, 12, 16, 20, 24

- Vital signs
- Review of symptoms, other medicines you are taking including DOT log, and a physical examination
- Adverse event assessment

BU/BMC Institutional Review Board
IRB NUMBER: H-32150
IRB APPROVAL DATE: 04/20/2016

RESEARCH CONSENT FORM (v6.0)

Patient Consent for Participation in the Research Study
For Persons 18 years of Age and Older

Title of Project: Prospective, Randomized, Blinded Phase 2 Pharmacokinetic/Pharmacodynamic Study of the Efficacy and Tolerability of Levofloxacin in Combination with Optimized Background Regimen (OBR) for the treatment of MDR-TB (DMID # 13-0057)

Principal Investigator: Dr. Charles Horsburgh

- Sputum collection
- Blood tests to check your liver, kidneys, blood count and blood sugar Urine or blood pregnancy test if you are a woman who can get pregnant
- Electrocardiogram (EKG)

The first sputum and one follow-up sputum after 3 months (if available) will be tested for TB by standard lab methods and may also be tested by new lab methods to learn how to best detect TB.

Risks and Discomforts

All medicines have possible side-effects. The list below gives side-effects that are known to occur with levofloxacin. Other side effects that we do not know about could occur with higher doses or when the drug is taken for a longer time, as it is in treatment of TB.

Known possible side effects from levofloxacin

- Constipation, diarrhea (could be severe and persistent), gas, nausea, stomach ache
- Headache; lightheadedness, dizziness
- Fast or irregular heart beat
- Rash, hives, itching, difficulty breathing, chest tightness, fever, chills
- Unable to move or carry heavy weights, muscle pain or weakness, pain in muscle or tendon due to inflammation or rupture
- Tingling, pain, burning, numbness or weakness
- Nightmares, hallucinations, confusion, seizures Increased risk of sunburn
- Bacterial infection other than TB, injury to the liver, and food tasting funny
- Symptoms of diabetes
- The need for more frequent glucose monitoring if you have diabetes.

NOTE: You may experience a side effect even after stopping the study treatment. In case of severe side effects, study medications will be discontinued but your treatment for TB will continued by your doctors. We will ask you to come back for a visit four (4) weeks after you take your last dose to check for any side effects.

BU/BMC Institutional Review Board
IRB NUMBER: H-32150
IRB APPROVAL DATE: 04/20/2016

RESEARCH CONSENT FORM (v6.0)

Patient Consent for Participation in the Research Study
For Persons 18 years of Age and Older

Title of Project: Prospective, Randomized, Blinded Phase 2 Pharmacokinetic/Pharmacodynamic Study of the Efficacy and Tolerability of Levofloxacin in Combination with Optimized Background Regimen (OBR) for the treatment of MDR-TB (DMID # 13-0057)

Principal Investigator: Dr. Charles Horsburgh

As you will be taking other medicines (OBR) with levofloxacin, it is important to know that those other drugs may have possible side-effects that are similar to the list above. The exact side effects depend on the types of medicines that you will be taking.

Risks from an HIV test.
Some people who have an HIV test might feel anxious. If you feel this way, you can talk about it with *(Name of PI at your site)* by calling *(tel #).* You will be told your HIV test result. You will be told the meaning of the test result, whether it is positive or negative. Positive HIV results are reported to *(Institution: other site specific information about HIV reporting).* HIV tests will be kept private according to *(Your Country's)* laws.

Risks from drawing blood.
There are a few small risks from taking four teaspoons of blood. These include brief pain from the needle stick, bruising, bleeding, lightheadedness, and rarely, infection where the needle enters the vein.

Information for Women
We do not know enough about the safety of levofloxacin for pregnant women or for the babies of women who are breastfeeding. You will not be allowed to take part in this study if you:

- are now pregnant,
- plan to become pregnant during study treatment,
- or are breastfeeding.

You will be tested regularly to see if you are pregnant. If you become pregnant during the study, you must tell the study nurse/coordinator or doctor right away. The study treatment will then be stopped. Your doctor will decide what TB treatment is best for you, but we will continue to follow you until the end of your pregnancy and your baby 6 months after being born.

If you become pregnant while you are participating in this study, it could be dangerous for the baby. You must use birth control if you are a woman having sex with men while you are participating in this study and for three months afterward. The only birth control methods that

BU/BMC Institutional Review Board
IRB NUMBER: H-32150
IRB APPROVAL DATE: 04/20/2016

work well enough to be safe while you are on this study are oral contraceptives (the pill), intrauterine devices (IUDs), contraceptive implants under the skin, contraceptive rings or patches or injections, diaphragms with spermicide and condoms with foam. You should not participate in this study if you are a woman who has sex with men and cannot use one of these birth control methods.

There may be unknown risks/discomforts involved. Study staff will update you in a timely way on any new information that may affect your health, welfare, or decision to stay in this study.

Potential Benefits

There may be no direct benefits to you from being in this study. Although it is possible that a higher dose of the levofloxacin could be better in treating TB, this is not known and is the purpose of the study. You also could be in the group that gets the standard dose of levofloxacin. Taking part in this research will help others by finding out if higher doses of levofloxacin are better for treating drug resistant TB without more side effects.

Alternatives

If you do not take part in this study, you will be treated for TB with the standard medicines, which may include levofloxacin.

Subject Costs and Payments

There is no cost to you for being in the study. You will not have to pay for any medicine or tests that are part of this study. You will be reimbursed for expenses you have that are a result of you being in the study. *(add site-specific information on reimbursement)*

Your blood specimen will be used only for research and will not be sold. You will not be paid for allowing your leftover blood specimen to be used in research even though the research done with your blood specimen may help to develop new products in the future. Similarly, there will be no cost to you for storing your left over blood specimen.

BU/BMC Institutional Review Board
IRB NUMBER: H-32150
IRB APPROVAL DATE: 04/20/2016

RESEARCH CONSENT FORM (v6.0)

Patient Consent for Participation in the Research Study
For Persons 18 years of Age and Older

Title of Project: Prospective, Randomized, Blinded Phase 2 Pharmacokinetic/Pharmacodynamic Study of the Efficacy and Tolerability of Levofloxacin in Combination with Optimized Background Regimen (OBR) for the treatment of MDR-TB (DMID # 13-0057)

Principal Investigator: Dr. Charles Horsburgh

Confidentiality

We will check your medical records to get information for the study. We will not use your name in any speech or paper about the study. We will not send your name to representatives from agencies such as the U.S. NIH or U.S. CDC. The U.S. FDA and study monitors from Westat (a research monitoring company) may also check your records. We will keep all information from your medical records private as much as the law requires.

Information from this study and from your medical record may be reviewed and photocopied by the U.S. Food and Drug Administration (FDA) and/or state and federal regulatory agencies such as the U.S. Office of Human Research Protection as applicable, and the Institutional Review Board of Boston University Medical Center. Information from this study and from your medical record may be used for research purposes and may be published; however, your name will not be used in any publications.
A description of this clinical trial will be available on http://www.ClinicalTrials.gov, as required by U.S. Law. This Web site will not include information that can identify you. At most, the Web site will include a summary of the results. You can search this Web site at any time.

Subject's Rights

By consenting to participate in this study you do not waive any of your legal rights. Giving consent means that you have heard or read the information about this study and that you agree to participate. You will be given a copy of this form to keep.

If at any time you withdraw from this study you will not suffer any penalty or lose any benefits to which you are entitled.

You may obtain further information about your rights as a research subject by calling the Office of the Institutional Review Board of Boston University Medical Center at 617-638-7207. If this study is being done outside the United States, you can ask the investigator for contact information for the local Ethics Board.

The investigator or a member of the research team will try to answer all of your questions. If you

BU/BMC Institutional Review Board
IRB NUMBER: H-32150
IRB APPROVAL DATE: 04/20/2016

---

have questions or concerns at any time, or if you need to report an injury while participating in this research, contact Dr. Robert Horsburgh at +1 617 638 7775 during the day or after hours.

The authority to collect this information is under 42 USC 285f. This Federal code allows the sponsor, U.S. National Institute of Allergy and Infectious Diseases (NIAID), part of the National Institutes of Health (NIH), to conduct and support research.

Compensation for Research Related Injury

If you think that you have been injured by being in this study, please let the local investigator know right away *(Insert name and contact information of local investigator)* and ask the investigator where treatment for injury would be available locally. Boston University Medical Center and the sponsor do not offer a program to provide compensation for the cost of care for research related injury or other expenses such as lost wages, disability, pain, or discomfort. You will be sent a bill for the medical care you receive for research injury if your medical insurance does not pay for your medical care. You are not giving up any of your legal rights by signing this form.

Right to Refuse or Withdraw

Taking part in this study is voluntary. You have the right to refuse to take part in this study. If you decide to be in the study and then change your mind, you can withdraw from the research. Your participation is completely up to you. Your decision will not affect your being able to get health care at this institution or payment for your health care. It will not affect your enrollment in any health plan or benefits you can get.

If you choose to take part, you have the right to stop at any time. If there are any new findings during the study that may affect whether you want to continue to take part, you will be told about them as soon as possible.

The investigator may decide to discontinue your participation without your permission because he/she may decide that staying in the study will be bad for you, or the sponsor may stop the study.

BU/BMC Institutional Review Board
IRB NUMBER: H-32150
IRB APPROVAL DATE: 04/20/2016

We may stop the study medicine if:

- you have bad side effects,
- the study doctor decides it is best for you to stop the medicine, the study medicine is not working against your TB,
- you do not take the medicines like you should if you become pregnant, or
- the study ends

If the study medicines are stopped, we will see you again four (4) weeks later after you stop taking medications to make sure there are no additional side effects.

Future Use and Stored Specimens

REPOSITORY operations are conducted according to detailed standard operating procedures which protect patient confidentiality. Data, blood and urine will be stored at the University of California, San Francisco. Patient information is systematically de-identified before release to recipient investigators. All identifying information is stored in a password protected computer which is not connected to a network or in paper records stored in locked filing cabinets and will not be released to investigators using your specimens. Physical access to computer and paper records is restricted to repository personnel. Access to this information and these specimens will be limited to researchers who propose a research study to identify markers of a treatment response, and whose proposal is approved by an independent review panel.

The researchers would like to save 2 of your blood samples and 2 urine samples in order to see whether tests could show whether someone with TB was going to get better with treatment or not. The samples would be kept by the University of California, San Francisco in the United States and would only be used to do test about TB. The samples will be kept indefinitely in storage. This is called a "Repository" like a bank for blood and urine.

If you choose to be in the study, you can decide at any time that you do not want yours blood and urine samples to be stored any more. You must then contact the study nurse or doctor and let them know that you do not want your samples to be stored any more. Any samples that have been stored will be destroyed. You can still stay in the study if you choose not to have your blood and urine stored, or decide you do not want them stored any longer.

BU/BMC Institutional Review Board
IRB NUMBER: H-32150
IRB APPROVAL DATE: 04/20/2016

RESEARCH CONSENT FORM (v6.0)

Patient Consent for Participation in the Research Study
For Persons 18 years of Age and Older

Title of Project: Prospective, Randomized, Blinded Phase 2 Pharmacokinetic/Pharmacodynamic Study of the Efficacy and Tolerability of Levofloxacin in Combination with Optimized Background Regimen (OBR) for the treatment of MDR-TB (DMID # 13-0057)

Principal Investigator: Dr. Charles Horsburgh

Please indicate here if you will let the researchers keep your samples for future research:

________YES, you may store my blood and urine samples for future tests that will not include human genetic testing.

________NO, you may not use my blood and urine samples for other future research. Destroy my unused samples at the end of this study.

BU/BMC Institutional Review Board
IRB NUMBER: H-32150
IRB APPROVAL DATE: 04/20/2016

RESEARCH CONSENT FORM (v6.0)

Patient Consent for Participation in the Research Study
For Persons 18 years of Age and Older

Title of Project: Prospective, Randomized, Blinded Phase 2 Pharmacokinetic/Pharmacodynamic Study of the Efficacy and Tolerability of Levofloxacin in Combination with Optimized Background Regimen (OBR) for the treatment of MDR-TB (DMID # 13-0057)

Principal Investigator: Dr. Charles Horsburgh

Signing this consent form indicates that you have read this consent form (or have had it read to you), that your questions have been answered to your satisfaction, and that you voluntarily agree to participate in this research study. You will receive a copy of this signed consent form.

Persons To Contact:
If you have questions about this research study; If you have questions about your rights as a research subject; If you think you may be having a problem from any of the medicines used in this study or feel that you have been harmed by this study, PLEASE CONTACT:

(Insert name and contact information of local investigator).

Consent Statement. "My signature below indicates that I agree to be in this study. I was given a chance to ask questions. I feel that my questions have been answered. I know that being in this study is my choice. I know that after choosing to be in this study, I may withdraw at any time. I have been told that I will receive a signed copy of this consent."

Please sign and use the following date format: DDMMMYYYY (e.g. 11Feb2012)

Subject (Signature and Printed Name) **Date**

Person Obtaining Consent (Signature and Printed Name) **Date**

Investigator or Designee (Signature and Printed Name) **Date**

Witness (Signature and Printed Name) **Date**
(optional per local IRB)

BU/BMC Institutional Review Board
IRB NUMBER: H-32150
IRB APPROVAL DATE: 04/20/2016
